# Hypercapnia dissociates neuronal and hemodynamic responses impairing neurovascular coupling and functional brain connectivity

**DOI:** 10.1038/s41467-026-71742-z

**Published:** 2026-04-13

**Authors:** Irmak Gezginer, Yi Chen, Valerio Zerbi, Zhenyue Chen, Daniel Razansky

**Affiliations:** 1 Institute for Biomedical Engineering and Institute of Pharmacology and Toxicology, Faculty of Medicine, University of Zurich, Zurich, Switzerland; 2 Department of Information Technology and Electrical Engineering, Institute for Biomedical Engineering, ETH Zurich, Zurich, Switzerland; 3 Department of Psychiatry, Faculty of Medicine, University of Geneva, Geneva, Switzerland; 4 Department of Basic Neurosciences, Faculty of Medicine, University of Geneva, Geneva, Switzerland; 5 Institute of Precision Optical Engineering, School of Physics Science and Engineering, Tongji University, Shanghai, China

**Keywords:** Functional magnetic resonance imaging, Fluorescence imaging, Neuro-vascular interactions

## Abstract

Neurovascular coupling (NVC) underpins the interpretation of hemodynamic signals as proxies for neural activity, yet its response to metabolic perturbations remains poorly understood. Here, we leverage concurrent fluorescence calcium imaging and functional magnetic resonance imaging in mice expressing genetically encoded calcium indicators to dissect how elevated CO₂ levels reshape the interplay between neural and vascular responses. Our findings indicate that hypercapnia induces opposing trends in calcium and blood-oxygen-level-dependent (BOLD) responses, accompanied by global desynchronization of brain activity. Additionally, 5% CO₂ suppressed sensory-evoked BOLD and hemoglobin responses, while neuronal and astrocytic activity remained unaffected. Dynamic functional connectivity and co-activation pattern analyses further reveal a dissociation in the coordination between BOLD hemodynamic responses and their underlying neural dynamics under hypercapnic conditions. Notably, we observed that hemodynamic responses, normally driven by neuronal signaling, get attenuated with BOLD signals no longer reflecting neural activity patterns in the brain. These findings demonstrate that hypercapnia can override conventional NVC relationships, compelling a reassessment of how BOLD contrast is interpreted under hypercapnic stress in preclinical and clinical settings. This holds particular relevance for chronic hypercapnia-related conditions where a deeper understanding of NVC disruption may inform improved diagnostic and therapeutic strategies.

## Abstract (web summary)

In this study, simultaneous imaging of neural and hemodynamic signals in the mouse brain under hypercapnia reveals disrupted neurovascular coupling, weakened brain connectivity, and limited reliability of BOLD signals as markers of neural activity.

## Introduction

A key concept linking neural activity to vascular responses is neurovascular coupling (NVC), the mechanism by which active neurons receive the necessary supply of oxygenated blood through the dynamic regulation of local blood flow^1^. Impairment in NVC is implicated in numerous neurological conditions, including stroke^2,3^, Alzheimer’s disease^2,4,5^, and traumatic brain injury^6,7^. The precise mechanisms underlying NVC remain incompletely understood. The current view favors the hypothesis that glutamate-mediated signaling initiates the release of nitric oxide (NO) from neurons and arachidonic acid metabolites from astrocytes, driven by rising intracellular Ca²⁺ concentrations^8^. These vasoactive molecules engage various signaling pathways in vascular smooth muscle cells and pericytes to induce vasodilation and increase cerebral blood flow (CBF) in response to neuronal activity.

One valuable model for probing NVC is hypercapnia, characterized by elevated levels of CO₂ in the blood. Hypercapnia induces widespread vasodilation across the brain’s vasculature, leading to increased CBF and decreased pH levels due to the formation of carbonic acid^9,10^. However, under hypercapnic conditions, the normal coupling between neural activity and vascular responses can be disrupted^9^. The impact of hypercapnia on neural activity itself has been a subject of debate, although the majority of results point to reduced neuronal excitability^12–15^. Exogenous CO_2_ has been shown to occlude functional hyperemia, the increase in blood flow associated with sensory stimulation^9,10^. Conversely, it has recently been reported that hypercapnic hyperemia and functional hyperemia do not activate the same vasodilatory signaling pathway mediating cerebrovascular responses^11,16^. A thorough understanding of this atypical neurovascular environment is essential for accurate interpretation of the blood-oxygen-level-dependent (BOLD) signals in functional magnetic resonance imaging (fMRI), as hypercapnia can result from various medical disorders such as sleep apnea and chronic obstructive pulmonary disease, as well as from behaviors like breath holding^17–19^. Additionally, CO_2_ is deliberately elevated in clinical settings for neuroprotective measures and enhancing neuroimaging contrast through calibrated fMRI^20–22^.

Studying the key mediators of NVC remains an arduous task as no single imaging modality can fully capture the intricate complexity of brain dynamics^23,24^. Among the available neuroimaging techniques, fluorescence (FL) imaging and fMRI stand out as highly complementary methods. In models expressing genetically encoded calcium indicators, such as GCaMP proteins, FL imaging detects calcium-sensitive fluorescent signals that reflect neuronal excitability with high temporal resolution, making it ideal for capturing rapid, transient neural events^25,26^. In contrast, fMRI leverages the BOLD signal to infer neural activity indirectly through changes in blood oxygenation and CBF. Recent work on functional connectivity (FC) has demonstrated that while FL calcium imaging and fMRI both reveal overlapping aspects of cortical functional organization, each modality also captures distinct facets of brain activity that are inaccessible to the other^27^. By reading out these multifaceted connectivity patterns simultaneously and examining how they evolve over time may enable the tracking of dynamic brain states—periods characterized by transient synchrony or desynchrony between different brain regions.

Grounded in the hypothesis that spatiotemporal patterns of FC can reveal the coupling and/or uncoupling between hemodynamics and neural activity, we devised a hybrid fluorescence magnetic resonance imaging system and investigated the FC responses in mice during hypercapnia. By employing dynamic functional connectivity (dFC)^28–31^, we isolated FC patterns reflective of neural activity, while co-activation pattern analysis (CAP)^32–34^ was used to characterize hemodynamic responses driven by neural activity. This integrated approach has allowed studying FL and BOLD responses to hypercapnia with a focus on synchronized brain activity thus providing a multidimensional perspective on neurovascular alignment under physiological stress.

## Results

### Moderate hypercapnia elicits disparate hemodynamic and neural responses in the mouse brain

**Fig. 1:**
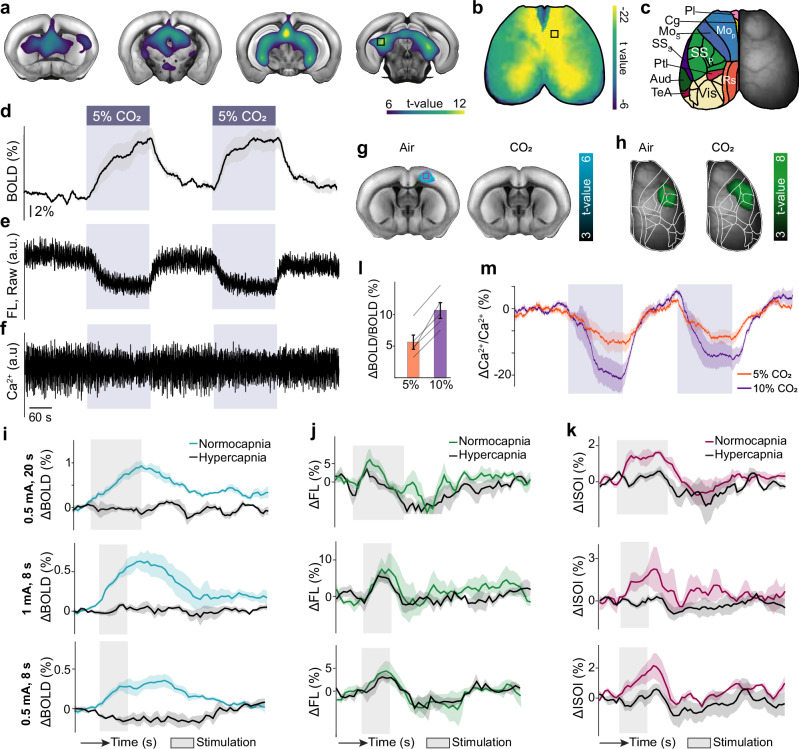
Group-level hypercapnia-evoked responses in simultaneous BOLD and FL recordings. **a** Multi-slice BOLD activation maps overlaid on the Allen mouse brain atlas. Hypercapnia induced widespread surge in the BOLD signal. **b** FL activations in response to hypercapnia. Significant reductions in the raw FL signal were detected throughout the visible cortical regions of the mouse brain. **c** FL mouse brain template generated by projecting the 3D Allen mouse brain atlas into 2D, focusing on cortical layers 1 to 4. **d** Group-averaged BOLD time courses from the region with the strongest hypercapnia-evoked responses (marked by a square in **a**), recorded during a 900 s paradigm alternating 180 s blocks of normocapnia and hypercapnia. **e** Raw FL signals exhibit a widespread decrease during hypercapnia due to increased hemoglobin concentration, pH-quenching of the GCaMP6f sensor and reduced neuronal Ca²⁺ transients. **f** Population Ca²⁺ transients recovered after regression of the concurrently recorded hemoglobin signal, band-pass filtering and transient amplitude scaling to correct for pH-quenching. BOLD (**g**) and FL (**h**) activation maps evoked by 0.5 mA, 20 s electrical forepaw stimulation under normocapnia (left) and 5% CO₂ hypercapnia (right), showing diminished BOLD response alongside preserved FL activation. Group-averaged (*n* = 4) BOLD (**i**), FL (**j**), and intrinsic optical signal (ISOI; 561 nm, hemoglobin-weighted) (**k**) responses to forepaw stimulation of varying durations and intensities during normocapnia and 5% CO₂, demonstrating attenuation of BOLD and ISOI responses under hypercapnia while FL responses remain unchanged. Pink squares in (**g**, **h**) denote the somatosensory cortex ROIs used for extracting the responses. **l** BOLD signal increase (mean ± SEM, *n* = 5) during 5% and 10% CO₂. **m** Ca²⁺ transient amplitude time-courses (*n* = 5) during 5% and 10% CO₂. Pl: prelimbic area; Cg: anterior cingulate area; Mo_p_: primary motor area; Mo_s_: secondary motor area; SS_p_: primary somatosensory area; SS_s_: secondary somatosensory area; Ptl: parietal area; Aud: auditory area; Rs: retrosplenial area; TeA: temporal association area; Vis: visual areas. Shaded areas represent SEM. Source data are provided as a Source Data file.

A hybrid magnetic resonance-epifluorescence imaging system (Supplementary Figs. S1 and S2) has been developed to obtain simultaneous BOLD and FL readouts from GCaMP mice exposed to moderate hypercapnia (5% CO₂: air/cO_2_ 950:50 mL/min). Fluorescence data were acquired from Thy1-GCaMP6f GP5.17 mice, in which GCaMP6f is expressed in excitatory pyramidal neurons (somata in layer V, apical dendrites in layers I–III)^35^. Wide-field epifluorescence thus predominantly reflected dendritic activity. Neuronal population signals were collected through an MRI-compatible fiberscope, synchronized with 9.4 T BOLD-fMRI. This multimodal setup enabled real-time, synchronized acquisition of neural and hemodynamic signals via concurrent BOLD, GCaMP and hemoglobin-weighted intrinsic recordings. Hypercapnia induced robust, bilateral responses in both modalities. The BOLD signal showed widespread increase across the brain spanning cortical and subcortical areas (Supplementary Fig. S3), consistent with previous findings under hypercapnic conditions^12,13^ (Fig. 1a). Meanwhile, the raw FL signal revealed a global decrease (Fig. 1b). To further dissect these effects, we parcellated the FL data using a 3D-to-2D projection of the Allen mouse brain atlas^36^, focusing on cortical layers 1 to 4 (Fig. 1c), as these layers are primary contributors to the signal detected in the epifluorescence channel^37^. This parcellation yielded 25 distinct cortical regions (see Supplementary Table 1). At the group level (*n* = 5), hypercapnia induced an 8.95 ± 0.51 % increase in the BOLD signal (Fig. 1d). It is important to note that the raw FL signal was contaminated by hemoglobin-induced and pH-quenching dynamics under hypercapnia (Fig. 1e). To isolate genuine population Ca²⁺ transients, we regressed out the concurrently recorded hemoglobin time course and applied a 0.2–4 Hz band-pass filter to remove low-frequency drifts and high-frequency noise. However, CO₂-driven acidification quenches GCaMP6f fluorescence, underestimating transient amplitude. Hence, we further scaled the filtered traces by the empirically determined pH-dependent attenuation factor (Fig. 1f, Supplementary Figs. S4 and S5, and Supplementary Note 1). Even after this correction, amplitudes of population Ca²⁺ transients (Supplementary Figs. S6 and S7), defined as the peaks of the analytic-signal envelope, remained significantly reduced during hypercapnia.

We next probed NVC by delivering electrical forepaw stimuli (0.5 mA, 20 s; 1 mA, 8 s; 0.5 mA, 8 s) under alternating normocapnia and 5% CO₂ (Fig. 1g–j). Under normocapnia, the stimulation evoked robust, graded increases in both BOLD and corrected Ca²⁺ signals across all paradigms. In contrast, hypercapnia suppressed the BOLD response while preserving neuronal calcium transients (Fig. 1g–j, and Supplementary Fig. S8). Intrinsic hemoglobin-sensitive (561-nm) responses were likewise attenuated under hypercapnia, consistent with the BOLD findings (Fig. 1k, and Supplementary Fig. S8). Because the 561-nm ISOI channel is not strictly isosbestic, its amplitude can be influenced by oxygenation shifts (i.e., changes in the ΔHbO/ΔHbR balance), which may be altered under hypercapnia. Accordingly, we interpret this signal as a hemoglobin-weighted hemodynamic readout rather than an oxygenation-independent HbT/CBV measure. To rule out potential attenuation of the BOLD response across repeated stimulations, we compared mean evoked BOLD amplitudes across successive normocapnia blocks and observed no decline, while BOLD remained suppressed throughout hypercapnia blocks (Supplementary Fig. S9). To test for vascular ceiling effects, we increased inhaled CO₂ to 10%, resulting in the BOLD amplitude rising further from 5.62 ± 1.12% to 10.64 ± 1.24% (two-sided paired *t*(4) = 6.56, *p* = 0.001) in primary somatosensory cortex, excluding saturation of vasodilation (Fig. 1l, and Supplementary Figs. S10 and S11). A comparable amplification was observed in the ISOI-derived absorbance signal when increasing CO₂ from 5% to 10%, and the across-animal changes closely tracked the corresponding BOLD increases (Supplementary Fig. S12). Conversely, neuronal Ca²⁺ transient amplitudes decreased by 12.18 ± 2.04% at 5% CO₂ and 24.59 ± 3.70% at 10% CO₂ (Fig. 1m), confirming that hypercapnia directly attenuates neuronal excitability despite preserved global vascular responsiveness to inhaled CO₂.

### Global desynchronization of brain activity occurs under hypercapnic stress

**Fig. 2:**
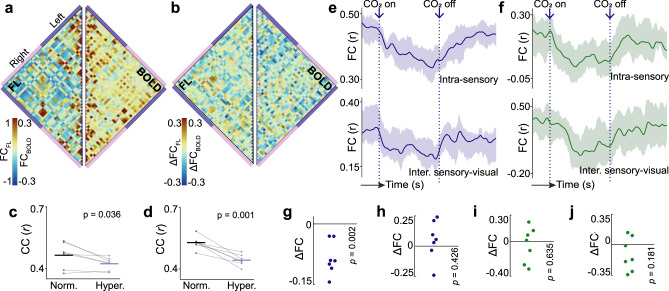
Hypercapnia-induced reductions in functional connectivity. **a** FL and BOLD functional connectivity (FC) under normocapnia, captured from simultaneous measurements. **b** FC changes to hypercapnia (hypercapnia minus normocapnia matrices). Correlation coefficient (CC) between BOLD and FL connectivity matrices (*n* = 7) for all the connections (**c**) and specific connections (*r* > 0.1) (**d**) during hypercapnia and normocapnia (two-sided paired *t*-test). Reduced intra-hemispheric connectivity within the somatosensory regions and inter-hemispheric connectivity between sensory and visual regions were detected by dynamic FC analysis in FL (**e**) and BOLD (**f**). Static FC analysis of the same regions in (**e**, **f**) of FL (**g**, **h**) and BOLD (**i**, **j**). Reduced intra-hemispheric (**g**, **i**) and inter-hemispheric (**h**, **j**) FC were not consistently detected by static measures. *p*-values of two-sided paired *t*-tests are shown on plots. Shaded areas represent SEM, *n* = 7 for all panels. Source data are provided as a Source Data file.

FC reflects the synchrony (or desynchrony) of low-frequency fluctuations between brain regions, providing measures of inter-regional communication. Building on our approach to characterize neurovascular dynamics by means of FC, we explored the impact of hypercapnia on FC across the brain. Region-wise FC was assessed by parcellating the brain using the 25-region template, considering identical regions in each modality. Both modalities exhibited strong inter- and intra-hemispheric synchrony under normocapnia, particularly within the somatomotor network, while weaker connectivity emerged between somatosensory and limbic areas (Fig. 2a). Hypercapnia disrupted these FC patterns, causing previously synchronized or opposing regions to become desynchronized, indicating a loss of inter-regional synchrony under metabolic stress (Fig. 2b). Further, the correlation between FC in the BOLD and FL channels decreased during hypercapnia (*p* = 0.036, paired *t*-test, Fig. 2c), particularly for specific correlations with Pearson’s r exceeding 0.1 (*p* = 0.001, paired *t*-test, Fig. 2d). To capture temporal variations in connectivity, we extended our analysis to dFC. This approach revealed a common reduction in intra-hemispheric synchrony within the somatosensory regions during hypercapnia, as well as weakened inter-hemispheric connectivity between sensory and visual regions in both modalities (Fig. 2e, f). These alterations were not consistently identified with static FC measures (Fig. 2g–j).

### Spatiotemporal patterns of multimodal cerebral fluctuations revealed through dFC and CAP analysis

We then set out to test the hypothesis that the temporal dynamics of brain connectivity undergo significant reorganization, particularly in response to conditions such as hypercapnia. Specifically, by examining these temporal dynamics, we aimed to uncover how metabolic stress reshapes connectivity patterns over time and to identify potential mechanisms underlying this network reconfiguration. To capture the transient and evolving shifts in neural and hemodynamic coordination, we employed dFC and CAP analyses. The dFC analysis was used in an attempt to isolate the component of brain signals that reflect the underlying neural activity and capture its alterations to hypercapnia (Supplementary Fig. S13). Time-resolved dFC matrices were generated using a sliding-window technique and clustered to identify recurring network configurations. Unlike dFC, which tracks continuous correlations, CAP analysis identifies discrete periods of global synchronization or desynchronization (Supplementary Fig. S13), thus providing a means to capture the spatiotemporal interaction of neurovascular components underlying the multimodal readings. Through these two approaches, we compared the neurovascular dynamics across modalities and metabolic conditions (i.e., normocapnia and hypercapnia).

### Hypercapnia disrupts neurovascular coupling and alters calcium dynamics

**Fig. 3:**
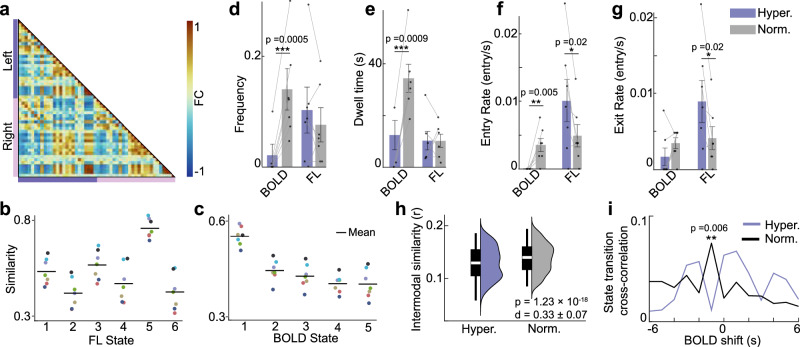
Multimodal dFC brain state alterations under hypercapnia. **a** FC matrix from fast (>3 Hz) FL transients, representing neural activity. Similarity of the resulting FL (**b**) and BOLD (**c**) dFC states, derived from low-frequency resting-state signals to the neural FC matrix (*n* = 7). Each subject is represented by a different color. **d**–**g** Frequency, dwell time (time spent in a state), entry rate (number of transitions into a state), and exit rate (number of transitions out of a state) of BOLD and FL state representing the underlying neural activity during normocapnic and hypercapnic conditions (*n* = 7). (*) *p* < 0.05, (**) *p* < 0.01, (***) *p* < 0.001; one-sided paired *t*-test. Error bars represent SEM. **h** Similarity of FL and BOLD dFC matrices in normocapnia (1668 dFC matrices from 7 mice) and hypercapnia (1201 dFC matrices from 7 mice). *p*, two-sample *t*-test; d, Cohen’s d effect size. The whiskers represent the minimum-to-maximum range, and the white line indicates the median value. **i** Cross-correlation under normocapnia and hypercapnia indicating similarity between the FL state transition vector and time-shifted versions of the BOLD state transition vector. (**) *p* < 0.01; *n* = 7, one-sided paired *t*-test. Source data are provided as a Source Data file.

Clustering the dFC matrices identified six distinct brain states in FL data and five in BOLD data, each characterized by a unique configuration of FC (Supplementary Figs. S14 and S15, see details in “Methods”). Connectivity matrices based on fast transients (>3 Hz) of FL data that reflect underlying calcium dynamics and thus neural population Ca²⁺ transients were further constructed (Fig. 3a). These neural dFC matrices exhibited very high temporal consistency, with correlations exceeding *r* > 0.99 across all time points (Supplementary Fig. S16). This contrasts with the variability observed in low-frequency FL (<0.1 Hz) and BOLD dFC matrices (Supplementary Fig. S17). FL state 5 and BOLD state 1 showed the strongest correspondence to neural FC patterns and with each other (Fig. 3b, c, and Supplementary Fig. S18). Thus, these states appear to reflect the underlying neural dynamics captured in the low-frequency FL and BOLD signals. However, these states did not consistently coincide (Supplementary Fig. S19). Remaining BOLD states showed lower alignment with neural patterns, suggesting they primarily reflect hemodynamic responses (Fig. 3c). Additionally, FL state 1 and BOLD state 5 exhibited high similarity to all states in the other modality—except for the states that reflect neural activity—suggesting that these baseline states capture broader hemodynamic changes or metabolic processes not directly tied to specific neural activity (Supplementary Fig. S18).

Hypercapnia led to profound changes within the FC dynamics associated with underlying neural activity. The frequency of neural activity-reflective BOLD state decreased under hypercapnia (*p* = 0.0005, paired *t*-test, *n* = 7) (Fig. 3d) and remained active for shorter periods of time (*p* = 0.0009, paired *t*-test) (Fig. 3e, and Supplementary Fig. S20). Interestingly, no transitions into the BOLD state reflecting neural activity occurred during hypercapnia (*p* = 0.005, paired *t*-test, Fig. 3f). In contrast, hypercapnia increased the rate of entries into the baseline state in BOLD readings (*p* = 0.02, paired *t*-test, Supplementary Fig. S21). FL data showed a contrasting effect, with heightened transitions into the neural-reflective mode (*p* = 0.02, paired *t*-test, Fig. 3f, g). The remaining states did not reveal significant alterations (Supplementary Fig. S22). The alignment of brain state transitions underscored the divergence between FL and BOLD readings as the inter-modal similarity decreased during hypercapnia (*p* = 1.23 × 10⁻¹⁸, two-sample *t*-test, Cohen’s *d* = 0.33 ± 0.07) (Fig. 3h). FL state transitions that precede BOLD state transitions by 1 s under normocapnia (*p* = 0.006, paired *t*-test) were further disrupted under hypercapnia with the temporal alignment of neural and vascular FC fluctuations reduced (Fig. 3i).

### CO₂ modulates spatiotemporal patterns of brain network coactivations and neural activity-driven hemodynamic responses

**Fig. 4:**
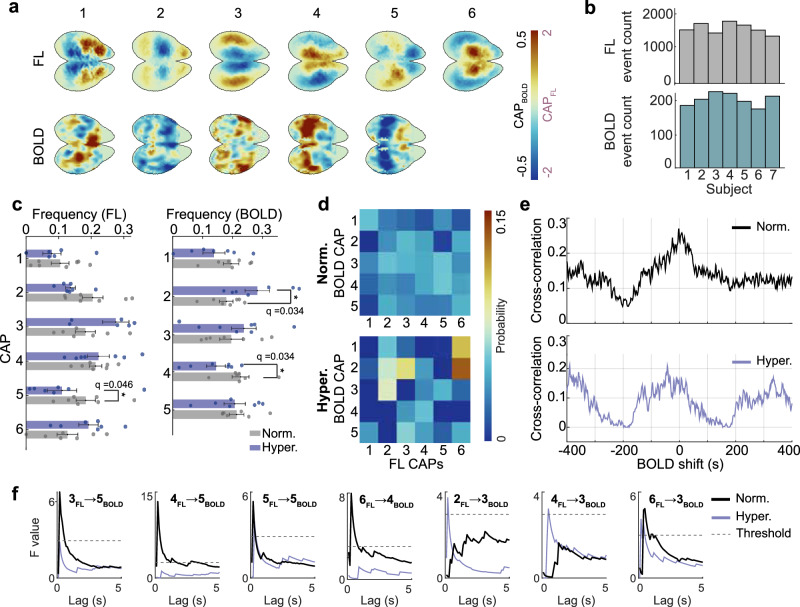
BOLD and FL brain CAPs under normocapnic and hypercapnic conditions. **a** CAP analysis identified distinct, recurring patterns of brain activity across FL and BOLD data, revealing six CAPs in FL and five in BOLD, capturing unique and overlapping co-activation dynamics in each modality. **b** Number of significant frames across animals. **c** Frequency of CAP occurrences (mean ± SEM, *n* = 7, one-sided paired *t*-test, FDR-corrected) in BOLD and FL data across normocapnic and hypercapnic conditions. (*) *q* < 0.05; paired *t*-test. **d** Co-occurrence of CAPs across FL and BOLD modalities under each condition. **e** Cross-correlation of FL and BOLD event vectors computed by shifting the BOLD vector relative to the FL vector and assessing their similarity at each shift. This approach indicates how closely the identified significant events align under normocapnia and hypercapnia. **f** Granger causality analysis of FL CAPs on BOLD CAPs capturing the directional influence across multiple BOLD time lags, with significance indicated by *F* ≥ 3. Source data are provided as a Source Data file.

Next, we applied CAP analysis to capture dynamic and discrete states characterized by global synchronization or desynchronization. Patterns of synchronized brain activity were clustered into six distinct CAPs in FL data and five in BOLD data (Fig. 4a, see details in “Methods”). The number of significant frames was consistent across animals (Fig. 4b). While certain CAPs were shared across FL and BOLD with high intermodal similarity, others remained modality-specific (Supplementary Fig. S23). Hypercapnia resulted in reduced occurrence of CAPs associated with somatosensory network activation (FL CAP 5, *q* = 0.047; BOLD CAP 4, *q* = 0.034, paired *t*-test, FDR-corrected at *α* = 0.05) (Fig. 4c). Conversely, a globally desynchronized cortical pattern (BOLD CAP 2) increased in frequency (*q* = 0.034, paired *t*-test, Fig. 4c) and entry rate (*q* = 0.026, paired *t*-test; Supplementary Fig. S24) under hypercapnic conditions. CAPs that were simultaneously present across the modalities were distributed relatively uniformly during normocapnia. Under hypercapnia, however, this uniformity was altered to a more selective co-occurrence pattern, as certain CAP pairs became more frequent while others diminished (Fig. 4d).

The temporal alignment of FL and BOLD CAPs was assessed by applying cross-correlation analysis to binary event vectors marking the presence of significant events. The strongest alignment during normocapnia was observed when the FL-captured neural events preceded the corresponding BOLD hemodynamic responses within 1–2 s, consistent with established NVC delays (Fig. 4e). Moreover, there was no periodic pattern linked to the breathing paradigm, indicating that the events were largely driven by endogenous brain activity. Under hypercapnia, the overall correlation decreased, revealing a rhythmic pattern synchronized with the timing of CO₂ inhalation cycles (Fig. 4e). This transition suggests that the global signals in FL and BOLD are becoming increasingly driven by the external CO₂ dynamics rather than spontaneous activity during hypercapnia.

To further characterize the causal relationship of neural and hemodynamic state transitions, we employed Granger causality analysis^38^. This method has enabled determining whether FL CAPs could predict subsequent BOLD CAPs. Under normocapnic conditions, FL CAPs 3, 4, and 5 triggered the occurrence of BOLD CAP 5 within a brief (~1 s) temporal window, consistent with established NVC delays (3_FL_ to 5_BOLD_, *p* = 0.001; 4_FL_ to 5_BOLD_, *p* = 1.64 × 10^−6^; 5_FL_ to 5_BOLD_, *p* = 0.005) (see Supplementary Table 2 for details on peak times and *F*-values) (Fig. 4f, and Supplementary Fig. S25). These predictive relationships were diminished under hypercapnia (3_FL_ to 5_BOLD_, *p* = 0.058; 4_FL_ to 5_BOLD_, *p* = 0.92; 5_FL_ to 5_BOLD_, *p* = 0.008). Conversely, FL CAPs 2, 4, and 6 predicted BOLD CAP 3 under hypercapnia (2_FL_ to 3_BOLD_, *p* = 0.024; 4_FL_ to 3_BOLD_, *p* = 0.006; 6_FL_ to 3_BOLD_, *p* = 0.011) whereas these predictive relationships were more variable under normocapnic conditions, often reduced in magnitude (2_FL_ to 3_BOLD_, *p* = 0.13; 4_FL_ to 3_BOLD_, *p* = 0.07; 6_FL_ to 3_BOLD_, *p* = 1.99 × 10^−4^).

### CO₂-induced neurovascular decoupling reveals cell-specific resilience

**Fig. 5:**
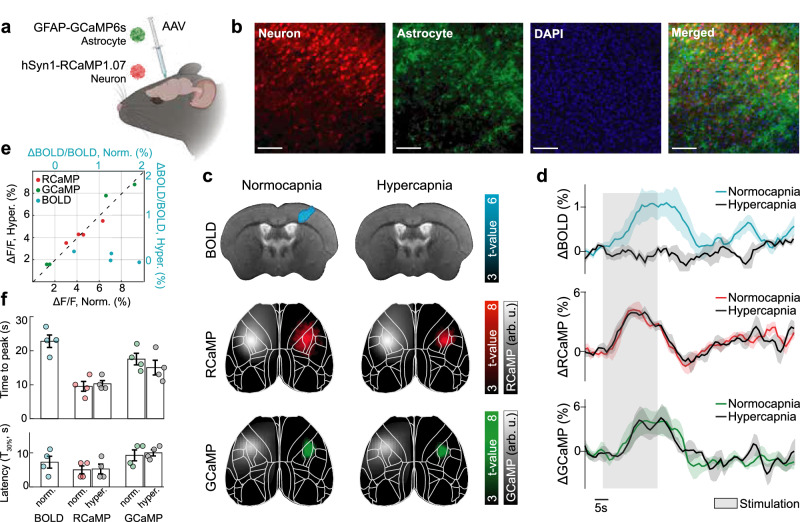
Concurrent imaging of neuronal, astrocytic and hemodynamic responses to forepaw stimulation under normocapnia and hypercapnia. **a** Viral co-transfection of GFAP–GCaMP6s and hSyn1–RCaMP1.07 into the forelimb subregion of primary somatosensory cortex for astrocyte- and neuron-specific Ca²⁺ imaging. Created in BioRender. Ge, I. (2026) https://BioRender.com/98hw24j. **b** Confocal ex vivo fluorescence images showing RCaMP1.07-labeled neurons (red), GCaMP6s-labeled astrocytes (green), and DAPI-stained nuclei (blue). Images shown are from one representative animal. Tissues were collected from 4 perfused animals. Scale bar, 100 µm. **c** Activation maps for BOLD, neuronal RCaMP1.07 and astrocytic GCaMP6s signals evoked by 0.5 mA, 20 s forepaw stimulation. Maps are shown for normocapnia (left) and 5% CO₂ hypercapnia (right), with BOLD overlaid on anatomical MRI and Ca²⁺ signals displayed next to the corresponding fluorescence images. Under hypercapnia, the BOLD response was abolished, whereas both neuronal and astrocytic Ca²⁺ signals remained robust. **d** Group-averaged (*n* = 4) time courses of BOLD, neuronal (RCaMP1.07) and astrocytic (GCaMP6s) responses to forepaw stimulation. Shaded areas represent SEM. **e** Percentage signal change for BOLD, RCaMP1.07 and GCaMP6s during normocapnia and hypercapnia (*n* = 4). **f** Time-to-peak and response latency values (mean ± SEM, *n* = 4) for each modality during normocapnia and hypercapnia. BOLD under hypercapnia is omitted due to undetectable activation. Source data are provided as a Source Data file.

The previous results highlight a clear discrepancy between the neuronal and hemodynamic responses under hypercapnic stress. To dissect the cellular basis of this neurovascular uncoupling under hypercapnia, in a cohort of C57BL/6 mice, we targeted astrocytes and neurons with AAV-mediated co-expression of GCaMP6s and RCaMP1.07, respectively, in the forelimb subregion of primary somatosensory cortex (Fig. 5a). Confocal ex vivo imaging confirmed robust, cell-type–specific reporter expression (Fig. 5b). Using our hybrid MRI–epifluorescence platform (Supplementary Fig. S2), we recorded simultaneous BOLD, astrocytic (GCaMP6s) and neuronal (RCaMP1.07) Ca²⁺ signals during 0.5 mA, 20 s forepaw stimulation under alternating normocapnia and 5% CO₂ hypercapnia (Supplementary Fig. S26).

Consistent with GCaMP6f recordings, hypercapnia completely abolished the stimulus-evoked BOLD response (Fig. 5c–e, and Supplementary Fig. S27), whereas both neuronal and astrocytic Ca²⁺ transients retained their amplitude and kinetics (Fig. 5c–f). Moreover, neither the time-to-peak nor the latency of the RCaMP1.07 or GCaMP6s responses were affected under hypercapnic conditions (Fig. 5f), indicating that moderate CO₂ selectively disrupts hemodynamic responsiveness without impairing neuronal or astrocytic calcium dynamics.

## Discussion

Stand-alone neuroimaging techniques offer only a fragmented view of the dynamic mechanisms underlying complex brain functions. We have shown that hybridization between FL and fMRI recordings mitigates these limitations by simultaneously capturing neural and hemodynamic activity. Although raw FL readings do not exclusively reflect neural Ca^2+^ transients owing to hemoglobin absorption and CO_2_-induced pH quenching, incorporating simultaneous hemoglobin readouts and pH-correction factors allowed for a reliable assessment of neurovascular alignment. The opposing trends observed in BOLD and Ca^2+^ signals highlight distinct responses to hypercapnia: while BOLD signal increase indicates hypercapnic hyperemia, reduction in GCaMP6f fluorescence transients reflects diminished neural excitability due to metabolic shifts associated with elevated CO_2_. Conversely, both modalities revealed a marked decline in inter-regional synchrony. The simultaneous reduction in both positive and negative correlations indicates a weakening of excitatory and inhibitory connectivity pathways, suggesting that elevated levels of CO_2_ disrupt the brain’s intrinsic ability to sustain coordinated activity across regions. As the system prioritizes CO_2_ clearance over localized neural demands, the balance between neural excitability and vascular support may get compromised, leading to diminished neural coherence across regions.

Our multiplexed imaging platform permitted concurrent readout of neuronal, astrocytic and hemodynamic signals during forepaw stimulation, overcoming drawbacks of single-modality NVC studies. Using this approach, we found that hypercapnia-induced neurovascular uncoupling also applies to evoked activity. Even mild hypercapnia completely suppressed the BOLD response, yet both neuronal and astrocytic Ca²⁺ transients retained full amplitude and timing. The persistence of glial Ca²⁺ signals confirms that upstream astrocyte activation remains unaltered, yet it no longer drives focal vasodilation, implying that a dominant CO_2_-mediated vasomotor tone overrides the normal astrocyte-to-vessel signaling cascade.

Since hypercapnia elevates baseline CBF/CBV and reduces baseline deoxyhemoglobin, prior work has proposed that BOLD’s dynamic range becomes compressed, such that stimulus-evoked changes in deoxyhemoglobin are very small even when neural drive persists^39,40^. In our data, concurrently acquired hemoglobin-weighted intrinsic signals (Hb-weighted ISOI) were likewise markedly attenuated during 5% CO₂, with only small residual responses in some cases. Accordingly, the parallel reduction of BOLD and hemoglobin-weighted responses despite preserved neuronal Ca²⁺ transients points to impaired NVC that cannot be attributed solely to baseline CBF/CBV shifts.

The integration of dFC and CAP analyses provided a multi-layered perspective on complex neurovascular alignment. dFC analysis captures temporal shifts in synchrony between regions, providing a more granular view of network interactions. This enabled the identification of operational modes that reflect the underlying neural dynamics inferred from multimodal data. Nonetheless, dFC’s reliance on sliding-window approaches may introduce variability by potentially omitting brief yet significant activities. In contrast, CAP analysis captures recurring, whole-brain snapshots of co-activation, offering a less temporally sensitive perspective on brain synchrony. Thus, we employed CAP analysis to elucidate how the relationship between calcium dynamics and hemodynamic responses is mediated under hypercapnic conditions. However, the lower temporal resolution of BOLD recordings imposes a floor on the temporal precision of FL–BOLD alignments. Thus, sub-second dynamics revealed in calcium imaging may therefore be partially obscured in our hemodynamic metrics. Note also that hypercapnia can induce non-linear vascular dynamics violating clustering and causality assumptions. Accordingly, our metrics should be interpreted as reflecting the primary linear interactions in the hypercapnic brain.

Unlike normocapnia, where both FL and BOLD showed coordinated transitions into neural-reflective states, hypercapnia hindered BOLD transitions and confined the readouts to a baseline vascular mode. This suggests that CO₂-driven vasodilation impairs NVC by reducing BOLD’s sensitivity to neural fluctuations. Consequently, while BOLD captures the broad vascular response to hypercapnia, it becomes less indicative of localized neural shifts. Interestingly, FL signals transitioned into neural states more frequently under hypercapnia, despite reduced intensity and coherence. This may indicate an adaptive shift in neural circuits, where localized bursts of activity are compensated to sustain essential functions despite decreased overall excitability. Overall, these findings suggest a convergence on common cellular pathways for both functional and hypercapnic hyperemia during prolonged stimulation. This implies that the observed resilience in NVC^11^ under hypercapnia may have resulted from submaximal NVC responses elicited by weak stimulation.

CBF dynamically adjusts to neural activity under normal NVC conditions. Accordingly, the temporal alignment of BOLD and FL responses mirrored typical neurovascular delays (~1–2 s) under normocapnia. Hypercapnia overrode this alignment by reducing the normocapnic FL-to-BOLD directional influences while introducing unique FL-to-BOLD pathways. Instead of uniformly suppressing or amplifying neural activity-driven responses, hypercapnia appears to selectively recalibrate connections, with FL-to-BOLD influences adjusting to facilitate CO₂ regulation. The concurrent reduction in sensory-related BOLD and FL CAPs during hypercapnia indicates a reallocation of resources from sensory processing circuits toward CO₂ regulation. This functional segregation may reflect the brain’s adaptive mechanisms for compartmentalizing vascular and cortical responses to better manage systemic challenges.

The present study’s findings, derived from an anesthetized, controlled hypercapnic model, may have limitations when generalizing to awake or freely behaving conditions. Anesthesia itself affects NVC and may amplify or alter the brain’s responses to hypercapnic stress relative to naturalistic conditions. Although the observed neurovascular dissociation was independent of sex and anesthesia regimen, future research may benefit from adapting multimodal imaging techniques to non-anesthetized models to better simulate clinical realities and improve the translational relevance of hypercapnia-induced neurovascular dynamics. Further, our wide-field epifluorescence in Thy1-GCaMP6f GP5.17 mice reports primarily the activity of superficial apical dendrites of layer-V pyramidal cells rather than deep somatic firing, which may influence the apparent amplitude and temporal dynamics of the calcium signals relative to BOLD. In addition, estimates of Ca²⁺ transient peak amplitudes under hypercapnia should be interpreted with caution as hemoglobin regression and pH scaling are approximate procedures that cannot guarantee full removal of vascular and acid–base influences. That said, our central conclusions pertaining to neural–hemodynamic uncoupling rely on correlation-based metrics that are insensitive to monotonic, multiplicative rescaling of the FL signal. Additionally, the projected fluorescence template restricted to superficial cortical layers allows for a close but not one-to-one spatial correspondence between modalities. While BOLD voxels in these superficial layers still integrate signal from capillaries and venules that drain deeper tissue, the wide-field fluorescence modality samples activity confined to the top ~150 µm of cortex and is blurred by photon scattering. Hence, a modest layer-integration mismatch remains.

Overall, our findings highlight critical issues in interpreting hemodynamic data under hypercapnic conditions. The observed decoupling of neural activity and vascular signals under hypercapnia complicates the interpretation of BOLD as an accurate representation of neural activity. Persistent neurovascular decoupling in hypercapnia-inducing conditions could additionally impair cortical synchrony, in turn impacting cognitive functions. Isolating brain states reflecting the underlying neural and vascular responses under metabolic stress could aid in assessing conditions such as stroke, Alzheimer’s or Parkinson’s disease, where neurovascular dysfunction serves as a key biomarker. The emergence of unique neurovascular pathways under stress may additionally inform improved therapeutic strategies aimed at restoring neurovascular balance, particularly for patients experiencing chronic hypercapnic conditions.

## Methods

### Hybrid magnetic resonance epifluorescence system

The hybrid system integrates a custom-made MRI-compatible fiberscope (Zibra Corporation, Westport, USA) for optical (epifluorescence) imaging into a 9.4 T high-field MRI scanner (BioSpec 94/20, Bruker BioSpin, Ettlingen, Germany) (Supplementary Figs. S1 and S2). The fiberscope features a 1.4 mm diameter optic image guide consisting of 100,000 single-mode fibers used for collecting the fluorescent responses surrounded by 19 multi-mode 600 μm core diameter fibers with 0.4 numerical aperture (NA) for optical excitation (Zibra Corporation, Westport, USA). Epifluorescence imaging was conducted by inserting the fiberscope’s tip into the aperture of a custom-made animal holder cover, aligned parallel to the static magnetic field of the MRI scanner. This setup was combined with a broadband dielectric mirror (BB1-E02, Thorlabs, USA) to redirect the illumination beam and the captured responses. At the output end of the image guide, two emission filters (FL514.5-10, Thorlabs, USA) were cascaded to isolate the fluorescence emission from GCaMP proteins, which was subsequently captured with an EMCCD camera (iXon Life, Andor, UK) at 40 fps. Fluorescence excitation was provided by a continuous wave 488 nm laser (Sapphire LPX 488–500, Coherent, USA). The captured fluorescence images in epi-illumination mode provided a 15 ×  15 mm^2^ field of view (FOV) with ~70 μm lateral resolution. A custom radiofrequency (RF) surface coil, integrated into the MRI setup, was positioned at the bottom of the 3D-printed animal holder, with the axis of the coil oriented perpendicularly to the static magnetic field. An external trigger device (Pulse Pal V2, Sanworks, USA) was used to synchronize the data acquisition of MRI and fluorescence imaging for precise temporal correlation.

For dual-wavelength experiments (simultaneous GCaMP/hemoglobin or GCaMP/RCaMP imaging), a second continuous-wave 561 nm laser (Sapphire LPX, Coherent, USA) was introduced (Supplementary Fig. S2)^41^. The same fiberscope was used for excitation light delivery and collection of optical signals. Emitted/returned light was split into two separate detection channels via a dichroic mirror (DM2, Di03-R532-t1-25×36, Semrock). The GCaMP emission, filtered by FF01-525/39-25 (Semrock), was captured by an EMCCD camera (Andor Luca, UK). The red channel was recorded by a CMOS camera at 40 fps (Basler Ace2, Germany) through a long-pass filter (FELH0600, Thorlabs). For neuronal Ca²⁺ imaging in the red channel (RCaMP1.07), the detection path was configured for fluorescence. For hemoglobin-weighted intrinsic-signal optical imaging (ISOI), the same 561 nm channel was operated in reflectance mode by removing the red-channel emission filter, so that changes in detected intensity were dominated by hemoglobin absorption.

At 561 nm, reflectance changes are strongly hemoglobin-weighted but are not strictly isosbestic; therefore, the signal reflects a wavelength-dependent combination of Δ[HbO] and Δ[HbR] rather than an oxygenation-independent measure of HbT/CBV. Accordingly, throughout the manuscript we treat the 561-nm intrinsic signal as a hemoglobin-weighted ISOI readout (Δ*R*/*R*), which is used to track stimulus-locked vascular dynamics and as a nuisance regressor for hemodynamic absorption cross-talk in the GCaMP channel, rather than as a quantitative HbT estimate.

### MRI data acquisition

MRI experiments were performed on a 9.4 T Bruker BioSpec 94/20 small-animal scanner equipped with custom-built RF coils, following an acquisition framework similar to that used in our previous work, with the parameters specified below^24^. T1-weighted anatomical reference images were acquired in the coronal plane using a FLASH sequence (FOV = 20 × 10 mm², matrix = 160 × 80, 11 slices, slice thickness  = 0.7 mm, TR = 500 ms, TE = 2.1366 ms, NA = 8). To support coregistration with the fluorescence data, MR angiography images were additionally collected using a 2D time-of-flight sequence (FOV = 20 × 20 mm², 20 slices, slice thickness = 0.3 mm, TR = 13 ms, TE = 1.8904 ms, FA = 80°, NA = 16). Before functional imaging, B0 field maps were used to optimize local magnetic field homogeneity. Functional data were then acquired with a gradient-echo echo-planar imaging sequence (FOV = 20  × 10 mm², matrix = 80 × 40, 11 slices, slice thickness = 0.7 mm, TR = 995 ms, TE = 12 ms, FA = 60°), resulting in a temporal resolution of 1 s over a total scan duration of 900 s.

### Animal models

GCaMP6f mice (C57BL/6J-Tg (Thy1-GCaMP6f) GP5.17Dkim/J, Charles River Laboratories, Germany, 24–52 weeks old, *n* = 13, 7 female, 6 male) and C57BL/6 mice (Charles River Laboratories, 10 weeks old, *n* = 4, female) were used in this study. Animals were housed in individually ventilated, temperature-controlled cages under a 12 h dark/light cycle. Pelleted food (3437PXL15, CARGILL) and water were provided ad-libitum. Mouse housing, handling, and experimentation were performed in accordance with the Swiss Federal Act on Animal Protection and were approved by the Cantonal Veterinary Office Zurich (ZH060/22).

### In vivo imaging

For a cohort of 13 animals (11 GCaMP6f and 2 C57BL/6 mice), anesthesia was induced by intraperitoneal administration of ketamine (100 mg kg^−1^ body weight, Pfizer) and xylazine (10 mg kg^−1^ body weight, Bayer). Supplemental anesthesia was provided intraperitoneally using ketamine (25 mg kg^−1^ body weight) and xylazine (1.25 mg kg^−1^ body weight). Twenty minutes after local administration of lidocaine (1%, 20 μL), the scalp was removed and bleeding was controlled using hemostatic sponges (Gelfoam, Pfizer Pharmaceutical). Functional imaging began 15 min after the first maintenance injection, when anesthesia had transitioned from the induction stage to a lighter and more physiologically stable plane.

For a second cohort of 4 animals (2 GCaMP6f and 2 C57BL/6 mice), anesthesia was initiated with 5% isoflurane in air and maintained below 1.5% during preparation. A bolus of medetomidine was then administered intravenously (0.05 mg/kg; Domitor, medetomidine hydrochloride; Pfizer Pharmaceuticals, Sandwich, UK). Five minutes later, isoflurane was reduced to 0.7%, and continuous medetomidine infusion (0.1 mg/kg/h) was started 10 min after the bolus. Functional imaging commenced 40 min after medetomidine administration and after completion of the structural MRI scan.

During imaging, mice were placed prone on a custom 3D-printed animal bed. Respiration and core temperature were continuously monitored using an MRI-compatible pneumatic pillow and rectal probe (SA Instruments, USA). Heart rate and peripheral oxygen saturation were recorded in real time with an MRI-compatible paw-mounted pulse oximeter (PhysioSuite, Kent Scientific Corporation, USA; Supplementary Fig. S28). Body temperature was maintained at approximately 37 °C using a temperature-controlled water-heating system. Concurrent fMRI and FL signals were acquired during a 15 min gas-challenge paradigm. The experiment began with 3 min of normocapnia using air (air/CO_2_ 1000:0 mL/min), followed by two cycles of hypercapnia and recovery, each consisting of 3 min of 5% or 10% CO_2_ inhalation (air/CO2 900–950:50–100 mL/min) and 3 min of normocapnia.

### Sensory stimulation

For sensory stimulation, unipolar rectangular electrical pulses (0.5 ms; 0.5 or 1 mA) were delivered to the left forepaw at 4 Hz. Each stimulation epoch lasted either 8 s or 20 s and recurred every 90 s (onset-to-onset). A schematic overview of the stimulation paradigm is provided in Supplementary Fig. S9. The electric signals were generated using a stimulus isolator device (Model A365R, World Precision Instruments, USA) fed by an external trigger (Pulse Pal V2, Sanworks, USA). During each hypercapnic and normocapnic period, two stimulation cycles were applied; no stimulation was delivered during the baseline normocapnia. All pulses were synchronized to both the camera and MRI acquisitions via the external trigger.

### Data preprocessing

For each subject, the first five BOLD volumes were discarded to account for scanner stabilization. Motion correction and smoothing with a Gaussian kernel (FWHM = 0.6 × 0.6 × 0.6 mm^3^) were performed using SPM12 (Wellcome Trust Centre for Neuroimaging, London, UK). The anatomical and functional MRI images were co-registered and spatially normalized to the Allen Brain Institute reference atlas, following previously described methods^36,42^. Each preprocessing output was visually inspected to ensure accuracy before proceeding with further analysis. The preprocessed BOLD data were analyzed for task-induced responses. For FC and CAP analyses, BOLD datasets underwent additional preprocessing steps, including band-pass filtering (0.01–0.1 Hz), detrending, and regression of motion parameters, ventricle signals, and white matter signals. The regression was shown to enhance the specificity of FC^43^. Dataset quality was assessed by examining the simultaneous presence of robust inter-hemispheric connectivity in sensory cortices (left S1_BF_ to right S1_BF_, r_FC_ > 0.1) and either weak connectivity or anti-correlation between sensory areas and the anterior cingulate areas (left S1_BF_ to ACA, r_FC_ < 0.1)^44^. Datasets meeting this criterion (7 out of 8) were included in further analyses.

Preprocessing of FL data was performed using SPM12 and custom MATLAB scripts. Raw FL images captured at 40 Hz were averaged to 10 Hz, and the first 5 s were discarded. Spatial smoothing with a Gaussian kernel (FWHM = 3 pixels) was applied. Data were corrected for motion and spatially normalized to the FL atlas (see “Template generation“). The output was visually inspected for accuracy. Data were then spatially resampled to a voxel size of 40 × 40 µm². To correct background fluorescence and ambient light interference, a background subtraction procedure was implemented. A 10 × 10-voxel ROI was manually selected from a non-fluorescent area of each image to represent the background signal. The calculated background signal time-course was subtracted from the fluorescence intensity of each voxel. The background-subtracted images were visually inspected to confirm the effectiveness of the subtraction process. FL images were then masked and analyzed for task-induced responses. For analysis of Ca²⁺ transients during hypercapnia, voxel-wise GCaMP6f signals were first coregistered, and concurrent hemoglobin fluctuations were regressed out using a general linear model (GLM). The residual GCaMP6f time series were then band-pass filtered between 0.2 and 4 Hz to remove low-frequency baseline drift and high-frequency noise. To correct for pH-dependent quenching of the GCaMP6f sensor, Ca²⁺ transients’ amplitudes during hypercapnic challenges were scaled by factors of 1.06 (10% CO₂) and 1.025 (5% CO₂) (see Supplementary Note 1). The envelope of the corrected Ca²⁺ transients was subsequently computed for further quantification. For forepaw stimulation, hemoglobin-corrected fluorescence signals were fed into a voxel-wise GLM to generate activation maps. Estimated motion parameters were included as nuisance regressors to minimize residual movement artifacts. For FC and CAP analyses, a different preprocessing pipeline was employed to focus on the low-frequency resting-state fluctuations in the FL data. For this purpose, FL datasets were detrended, band-pass filtered (0.01–0.1 Hz), and regressed for motion parameters and the global signal.

For resting-state analyses, population Ca²⁺ transient amplitude denotes the envelope peak of the hemoglobin-regressed, 0.2–4 Hz band-pass filtered, and (where indicated) pH-scaled fluorescence signal, namely, an ensemble measure rather than single-neuron action potentials (Fig. 1f, l). For evoked responses, fluorescence is reported as Δ*F*/*F* relative to a pre-stimulus baseline, whereas activation maps are estimated with a voxel-wise GLM. Traces shown in figures are temporally smoothed with a moving average filter (size: 1 s) for better visualization (Figs. 1j and 5d).

### Template generation

Region definitions of the BOLD template for the FC analyses were based on the Allen Common Coordinate Framework (CCFv3). To enhance the correspondence and comparability with FL signals, only layers 1 to 4 of the cortical areas representing the motor, somatosensory, visual, limbic, and association networks (25 distinct regions from each hemisphere) were included in the 3D BOLD template (see Supplementary Table 1 for the full list of regions). These layer definitions were chosen due to the direct involvement of the superficial cortical layers in the generation and propagation of neural signals detectable by FL imaging^37^. 3D to 2D projection of the BOLD template was performed to generate the FL template. Consequently, both BOLD and FL templates contain 25 identical functionally distinct regions from each hemisphere. For the remaining analyses, the images were masked, and ROI time-courses were calculated from the definitions based on the generated templates.

### Task-related activation analysis

Task-related responses to hypercapnia and forepaw stimulation were analyzed using MATLAB (R2023b) and SPM12. BOLD and FL task-induced responses were modeled using a GLM to obtain *t*-value maps^45^. The first-order canonical basis set was used, involving the convolution of the hemodynamic response function (HRF) with the stimulation paradigm as a regressor. The default HRF parameters in SPM12, optimized for human studies, were modified with parameters suitable for small animal BOLD responses^46^. To account for movement artifacts, six motion parameters calculated during the preprocessing step were included as covariates^47^. Activation maps for each animal were obtained by applying an initial threshold of uncorrected *p* < 0.001 to the *t*-value maps, and voxels were considered statistically significant after family-wise error correction at *p* < 0.05 using one-sided *t*-tests. The group-averaged activation maps were calculated by averaging the individual thresholded maps (*n* = 5). BOLD and FL group activation maps were overlaid on the Allen mouse brain atlas and the FL template, respectively. Exemplary responses were plotted from the regions with the strongest responses in the group activation map (0.4 × 0.4 mm² for FL, 0.4 × 0.4 × 0.4 mm³ for BOLD). The percentile changes at the group level were given as mean ± SEM.

### Static functional connectivity analysis

The static FC was assessed using the CONN toolbox for BOLD and FL data. The ROIs were defined by the generated BOLD and FL templates. The preprocessed time-series data were averaged within each ROI. For each subject, region-to-region connectivity matrices were constructed for hypercapnia and normocapnia conditions by computing region-wise Pearson’s correlation coefficients (*r*). Group-level matrices were computed by averaging the individual subject matrices (*n* = 7) for normocapnia. The group-level difference matrices (hypercapnia minus normocapnia) were computed. Pearson’s correlation coefficient (CC, MATLAB function corr) was used to assess the similarity of connectivity matrices for all connections or only the specific connections exceeding the 0.1 value. Neural correlation matrices were computed by considering high-frequency (>3 Hz) FL signals to capture FC specific to calcium activity.

### Dynamic functional connectivity analysis

Dynamic FC (dFC) was assessed using a sliding-window approach^48^. Pearson’s correlation coefficients were calculated between region pairs within a time window. The window length was set to 30 s, consistent with previous research^49^, corresponding to 30 frames for BOLD data and 300 frames for FL data. The window was then shifted forward by one TR_BOLD_ frame (1 s), resulting in 866 dFC matrices per animal. The temporal alterations in FC between ROI-pairs were plotted for FL and BOLD data. To identify recurring connectivity patterns (dFC states), *k*-means clustering was applied to the dFC matrices. The optimal number of clusters was determined by (1) the Silhouette method (Supplementary Fig. S29)^50^, (2) ensuring that each state was consistently identifiable in at least 6 out of 7 subjects, and (3) confirming that the identified states were sufficiently distinct from one another (with inter-state correlations of *r* < 0.9). Clustering was performed independently for each modality with 500 iterations and 10 replicates (MATLAB function kmeans). The dFC matrices were grouped into 5 clusters for BOLD and 6 for FL. Neural FC matrices were constructed for each time window using high-frequency FL data. The similarity between multimodal and neural states was estimated by calculating correlations. To assess the temporal stability, we calculated the correlation across the dFC matrices for neural states and FL/BOLD states. The cross- and within-modality similarities of the states were similarly estimated by Pearson’s correlation.

Several temporal metrics were calculated for each subject during hypercapnia and normocapnia, including the frequency of occurrence (the proportion of presence of each state), dwell time (the average presence duration time of each state), entry rate (how often the brain transitions into each state), and exit rate (how often the brain transitions out of each state). Paired *t*-tests were used to assess differences between hypercapnia and normocapnia. Transition probability matrices of each animal were constructed by normalizing the transition counts observed during hypercapnia and normocapnia periods. Significant differences between conditions were estimated using paired *t*-tests. The intermodal similarity was calculated for each dFC window by calculating the Pearson’s correlation between the FL and BOLD dFC matrices. The difference between normocapnia and hypercapnia was assessed by two-sample *t*-test and Cohen’s *d*. The temporal alignment between the state transitions across the modalities was analyzed by computing the cross-correlation (maximum lag = 6 s) across the binary state transition vectors. One-sample *t*-tests were conducted to determine the significant lags for hypercapnia and normocapnia conditions.

### Co-activation pattern (CAP) analysis

CAP analysis was employed to identify and characterize transient, whole-brain patterns of simultaneous activation and deactivation across multiple brain regions^33^. This approach enables the association of resting-state activity with discrete events, capturing information from relevant time points that would otherwise be spread across a conventional full-time-course analysis^34^. BOLD and FL data were clustered independently using the same analytical framework. For each subject, significant activation and deactivation were identified at the voxel level by comparing the time-point data against subject-specific thresholds, defined as two standard deviations above or below the mean for both BOLD and FL data. This was conducted for the voxels within BOLD and FL templates. A frame was classified as a significant event if at least 5% of the voxels exhibited significant activation or deactivation at that time-point. The selected frames were then organized into *TxN* dimensional vectors, where *T* represents the total number of selected frames across subjects and *N* corresponds to the number of voxels within the mask. These frames were clustered using *k*-means (500 iterations, 10 replicates, MATLAB function kmeans), generating centroids representing the average activity patterns for each CAP. The centroids were subsequently reshaped into their original spatial dimensions to create CAPs (Fig. 4a). For the BOLD CAPs, maximum intensity projection (MIP) was used to visualize the patterns. Spatial correlation (Pearson’s r) matrices were calculated between 2D FL and MIP BOLD CAPs to assess the similarity between and within the CAPs identified in the BOLD and FL data. The optimal number of clusters was determined using (1) the Silhouette method (Supplementary Fig. S30), ensuring bilateral spatial maps were obtained, and (3) verifying that all CAPs were identifiable in at least 6 out of 7 subjects. The dynamics of the CAPs from the selected number of clusters (*k*_BOLD_ = 5, *k*_FL_ = 6) were further analyzed.

The frequency of occurrence for each CAP was calculated separately for each subject during hypercapnia and normocapnia periods (Fig. 4c). Paired *t*-tests were conducted to determine whether these frequencies differed between the two conditions with multiple comparisons corrected using the Benjamini–Hochberg method to control the false discovery rate (FDR) at a level of *α* = 0.05. For each CAP, the entry/exit rates were calculated by counting the transitions from any other CAP to the specific CAP during the relevant period (hypercapnia or normocapnia) and normalizing by the total duration of each period. Exit rates were similarly calculated. Significant differences in entry and exit rates between conditions were identified using paired *t*-tests with FDR-corrected at *α* = 0.05. Simultaneously occurring CAP-pairs were identified by upsampling the BOLD CAP vector to match the FL temporal resolution. This yielded co-occurrence matrices for hypercapnia and normocapnia conditions (each sized *k*_FL_×*k*_BOLD_), normalized by the total concurrent events for each condition (Fig. 4d). Temporal synchrony of BOLD and FL event vectors was assessed through cross-correlation analysis. Binary event vectors were generated for each modality and condition. To check the temporal correspondence between the identified BOLD and FL events, the normalized cross-correlation of BOLD and FL event vectors was computed (maximum lag = 400 s) (Fig. 4e).

Granger causality analysis was conducted to assess whether the occurrence of an FL CAP could predict a subsequent BOLD CAP across various time lags^38^. For this process, the BOLD event vector was upsampled tenfold by replicating each 1 s sample across ten 100 ms bins to match the 100-ms resolution of the fluorescence data. For each FL-BOLD CAP pair, two models were estimated: a restricted model, where the BOLD CAP occurrence vector was regressed only on its own lagged values, and an unrestricted model, where it was regressed on both its own and the lagged values of the FL CAP vector. The residual sums of squares for each model were then compared to calculate an F-statistic that indicates whether including the FL CAP as a predictor significantly improved the BOLD CAP prediction. F-statistics above 3 were deemed significant, suggesting that the FL CAP provided predictive information about the BOLD CAP. This analysis, performed across lags up to 5 s, identified the temporal window where directional influence was strongest to identify the causal pathways between FL and BOLD global synchronization events (Fig. 4f).

### Induction of calcium indicator expression

Adeno-associated viral (AAV) vectors encoding GCaMP6s (AAV-9/2-hGFAP-hHBbI/E-GCaMP6s) and RCaMP1.07 (AAV-9/2-hSYN1-ch1-RCaMP1.07) were mixed 1:1 (v/v) immediately prior to injection, yielding final physical titers of 1.0 × 10¹³ vg/mL for GCaMP6s and 4.3 × 10¹² vg/mL for RCaMP1.07. Three weeks before in vivo imaging, the mice were anesthetized with isoflurane (5% induction, 1–2% maintenance) and secured in a stereotaxic frame. Two 0.5 mm burr holes were drilled bilaterally at Bregma +0 mm, lateral ±1.8 mm, and a 33-gauge beveled needle (NF33BV, World Precision Instruments) mounted on a NanoFil 10 μL syringe (World Precision Instruments) was lowered to a depth of 300 μm in the somatosensory (forepaw) cortex. Using a programmable hydraulic pump, 500 nL of the virus mixture was delivered per site at 50 nL/min. Throughout the injection, core body temperature was maintained at 37 °C, and physiological parameters were continuously monitored. After infusion, the needle remained in place for 5 min before slow retraction. The scalp was then sutured, and mice received buprenorphine (0.1 mg/kg, s.c.) for 24 h post operatively. Body weight was recorded daily for 3 days, then at least thrice weekly until the terminal experiment.

### Immunohistochemistry

Mice were anesthetized with ketamine (100 mg/kg), xylazine (10 mg/kg), and acepromazine maleate (2–3 mg/kg) (total volume 100 µL), then transcardially perfused, first with phosphate-buffered saline (PBS) and subsequently with 4% paraformaldehyde (PFA) in PBS. Extracted brains were post-fixed in 4% PFA for 3 h at 4 °C, followed by cryoprotection in 30% sucrose in PBS for 3 days. Specimens were embedded in optimal cutting temperature (OCT) compound, sectioned into 50-μm-thick slices using a cryotome (CM3050S, Leica, Germany), and counterstained with DAPI (4′,6-diamidino-2-phenylindole) for 5 min. Fluorescent images were captured using a Zeiss LSM 900 confocal microscope.

### Statistics & reproducibility

All quantitative results are reported as mean ± s.e.m. unless otherwise stated. Sample sizes for each experiment are indicated in the corresponding Results, Methods and figure legends. Sample sizes were chosen on the basis of previous experience with these multimodal imaging paradigms, consistency with prior related studies, and practical and ethical considerations for small-animal imaging experiments. No statistical method was used to predetermine sample size. All measures were compared across conditions using paired or two-sample *t*-tests as specified in the main text, figure legends, and methods. Multiple comparisons were controlled where indicated using the Benjamini–Hochberg FDR correction at *α* = 0.05. Granger causality analysis was implemented as an *F*-test comparing restricted and unrestricted autoregressive models, as described in the methods. Exact *p* values, *q* values, test directionality, and correction procedures are provided in the corresponding figures, legends, or supplementary tables. Resting-state dataset quality was assessed using predefined functional-connectivity criteria based on expected interhemispheric sensory connectivity and sensory-to-cingulate connectivity. One of eight resting-state datasets did not meet these criteria and was excluded from further resting-state analyses; thus, resting-state analyses were performed on 7 animals. No other datasets were excluded.

### Reporting summary

Further information on research design is available in the Nature Portfolio Reporting Summary linked to this article.

## Supplementary information

Supplementary Information

Reporting Summary

Transparent Peer Review file

## Source data

Source Data

## Data Availability

The data that support the findings of this study are available from the corresponding author. All data supporting the findings of this study are found within the paper and its Supplementary Information. Source data are provided with this paper.

## Code availability

Examples of custom codes used in this manuscript are deposited and publicly available on Zenodo at 10.5281/zenodo.19099592.
