# Supplementary material for: Hypercapnia dissociates neuronal and hemodynamic responses impairing neurovascular coupling and functional brain connectivity
Source: Nat Commun. 2026 Apr 13;17:5158. doi: 10.1038/s41467-026-71742-z (PMC13249851; doi:10.1038/s41467-026-71742-z)
Supplement: Supplementary file 1 — Supplementary Information [file 41467_2026_71742_MOESM1_ESM.pdf]

# Supplementary Material

# Hypercapnia dissociates neuronal and hemodynamic responses impairing neurovascular coupling and functional brain connectivity

Irmak Gezginer[1,2], Yi Chen[1,2], Valerio Zerbi[3,4], Zhenyue Chen[5,*], and Daniel Razansky[1,2,*]

[1] Institute for Biomedical Engineering and Institute of Pharmacology and Toxicology, Faculty of Medicine, University of Zurich, Switzerland

[2] Institute for Biomedical Engineering, Department of Information Technology and Electrical Engineering, ETH Zurich, Switzerland

[3] Department of Psychiatry, Faculty of Medicine, University of Geneva, Switzerland

[4] Department of Basic Neurosciences, Faculty of Medicine, University of Geneva, Switzerland

[5] Institute of Precision Optical Engineering, School of Physics Science and Engineering, Tongji University

[*] Correspondence

Zhenyue Chen, Institute of Precision Optical Engineering, Tongji University, 1239 Siping Road, Yangpu District, 200092 Shanghai, China

Email: zhenyue_chen@tongji.edu.cn

Daniel Razansky, Institute for Biomedical Engineering, Wolfgang-Pauli-Str. 27, 8093 Zurich, Switzerland

Email: daniel.razansky@uzh.ch

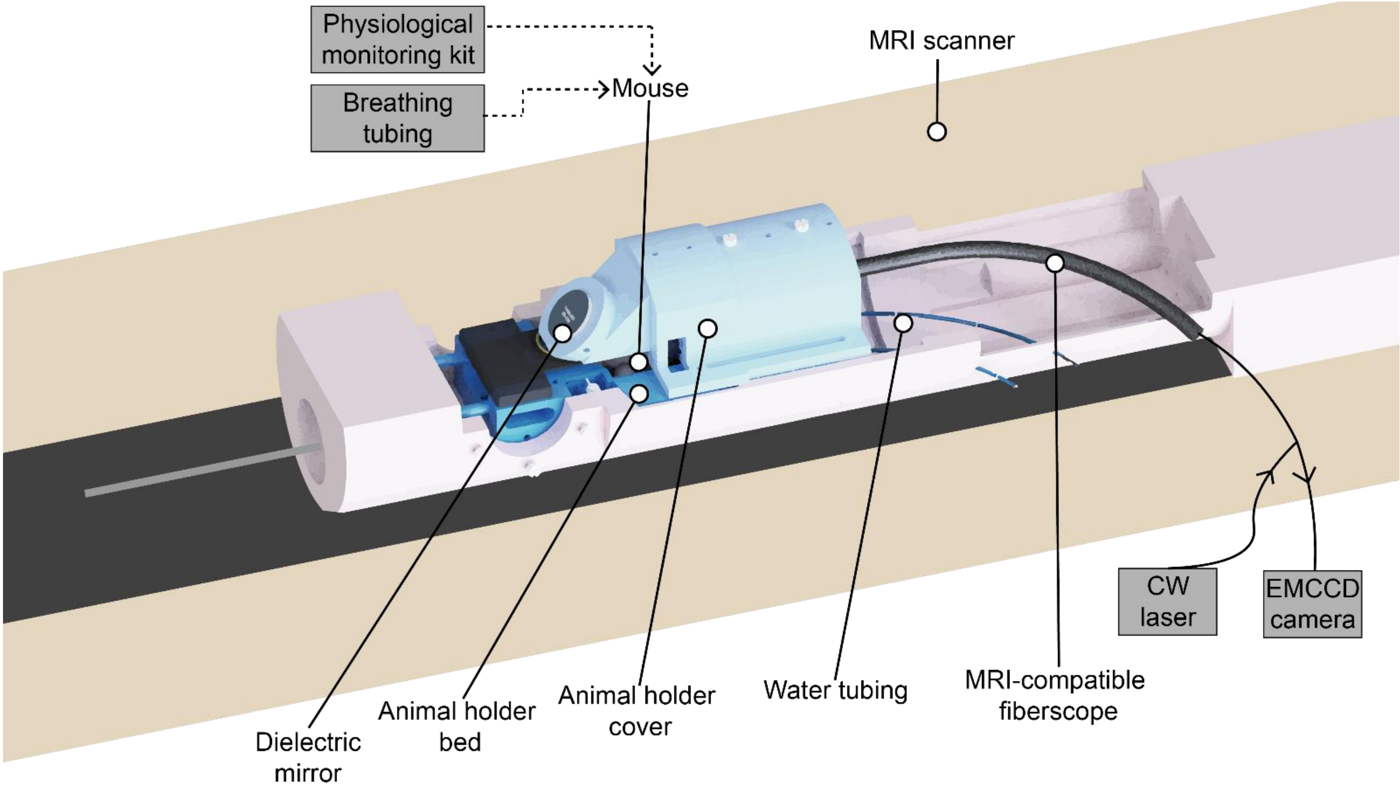


**Supplementary Figure S1** Layout of the hybrid system for concurrent FL and fMRI recordings. This hybrid system integrates an MRI-compatible fiberscope with an optic image guide and an EMCCD camera to capture GCaMP fluorescence images, combined with a 9.4 T MRI scanner for fMRI. Components include a continuous-wave (CW) laser for fluorescence excitation, a dielectric mirror for illumination redirection, and an MRI RF surface coil located on the animal holder bed. An external trigger synchronizes FL and fMRI acquisition for precise temporal alignment.

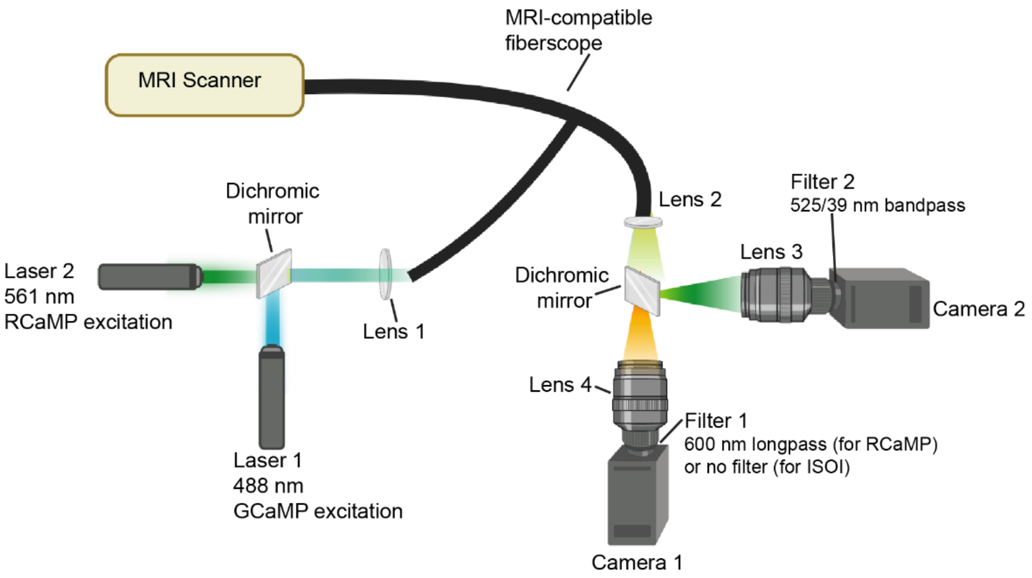


**Supplementary Figure S2.** Schematic of the hybrid fluorescence–MRI platform enabling dual-wavelength recordings. Two continuous-wave lasers (488 nm for GCaMP excitation; 561 nm for hemoglobin monitoring or RCaMP excitation) are combined and delivered via a custom, MRI-compatible fiberscope. Emitted fluorescence is collected through the same fiberscope, separated by a dichroic mirror into GCaMP and RCaMP (or hemoglobin) channels to prevent crosstalk, and recorded by two dedicated cameras at 40 Hz. By removing the emission filters, the same optical path can be used for intrinsic signal optical imaging (ISOI) mode. Created in BioRender. Ge, I. (2026) https://BioRender.com/pq80ck9.

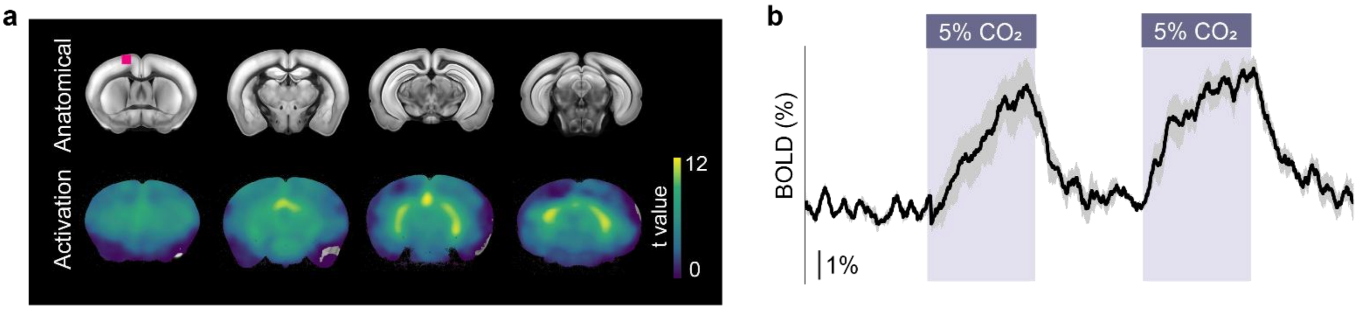


**Supplementary Figure S3. a** Unthresholded group-level (N = 4) BOLD activation map for the hypercapnia > normocapnia contrast, identical to the data in Fig. 1a. Top row, anatomical reference; bottom row, unthresholded t-map revealing widespread positive responses across cortex in addition to subcortical regions. **b** Group-averaged BOLD time course (mean ± SEM; N = 4) extracted from the cortical ROI indicated by the pink square in panel a, matching the ROI used for the FL responses in Fig. 1b.

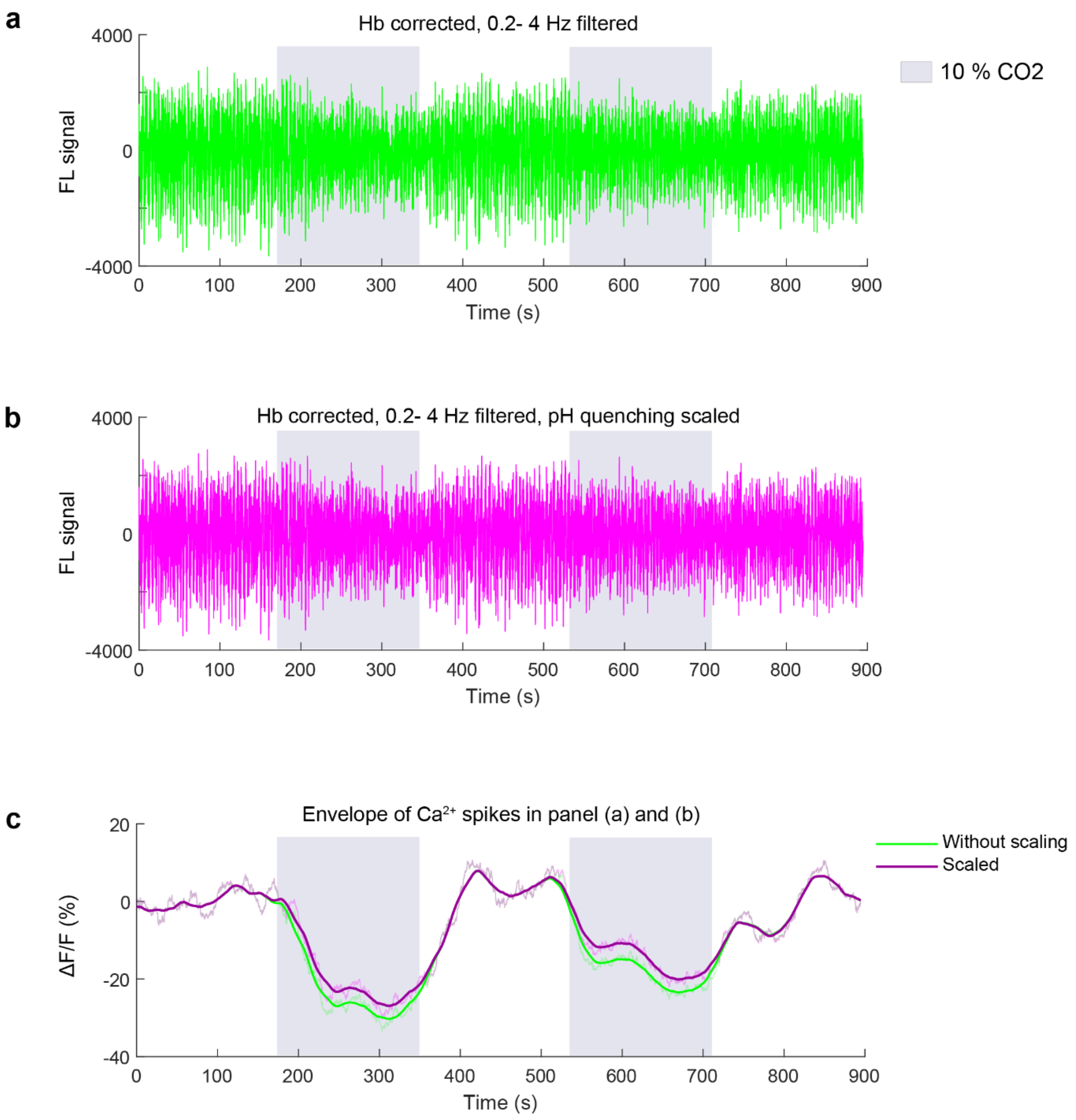


**Supplementary Figure S4.** Workflow for correcting fluorescence signals for hemodynamic and pH-quenching artifacts during hypercapnia. **a** Raw fluorescence time course was regressed against the concurrently recorded hemoglobin signal to remove blood-flow and volume–related artifacts, then band-pass filtered (0.2–4 Hz) to eliminate low-frequency drift and high-frequency noise. **b** Hemoglobin-corrected trace scaled by the $CO_2$-induced fluorescence reduction factor measured at 10 % $CO_2$ (see Supplementary Note 1) to compensate for pH-quenching of the calcium sensor. **c** Overlaid envelopes of calcium transient amplitudes before (green) and after (pink) pH-quenching scaling.

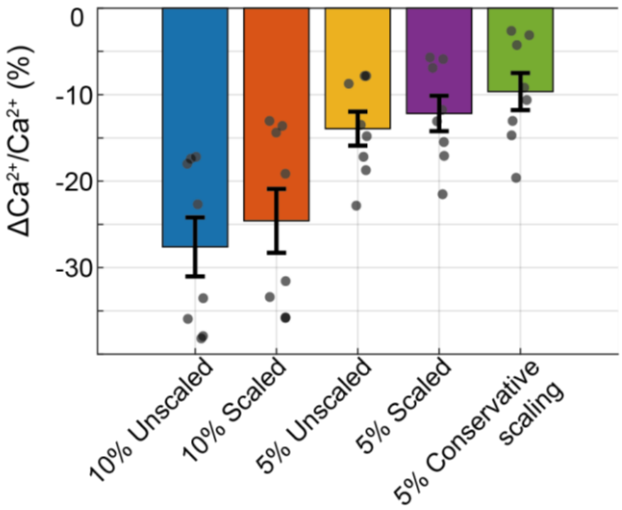


**Supplementary Figure S5**. Group-level (n = 4) $Ca^{2+}$ transient amplitude reduction during 5 % and 10 % $CO_2$ hypercapnia after pH-quenching scaling (S = 1.025 for 5 %, S = 1.06 for 10 %; see Supplementary Note 1), with a conservative test applying the 10 % scaling factor to the 5 % cohort (2.2× correction) still yielding a 9.6 % reduction in amplitude of $Ca^{2+}$ transients.

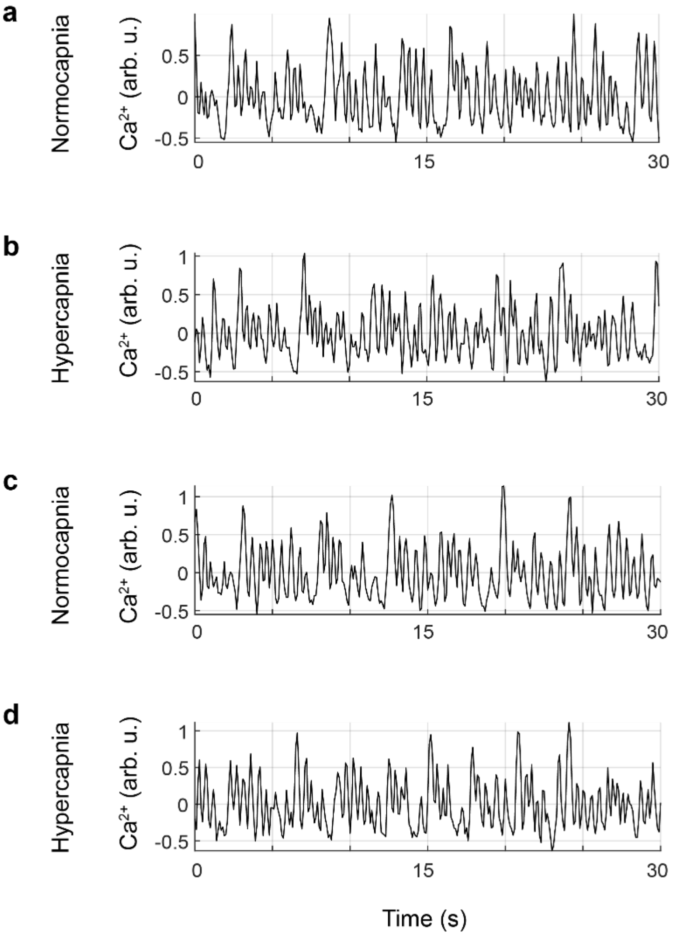


**Supplementary Figure S6**. Representative fast population calcium transients across normocapnia and hypercapnia. **a-d** Expanded 30-s segments of the hemoglobin-regressed, band-pass filtered GCaMP signal (0.2–4 Hz) shown for the first normocapnia block (a), first hypercapnia block (b), second normocapnia block (c), and second hypercapnia block (d). The fast calcium transients appear as structured, event-like fluctuations with a rapid rise followed by a slower decay and are present in both normocapnic and hypercapnic periods. A schematic of the stimulation timing and block structure is provided in Supplementary Fig. S9.

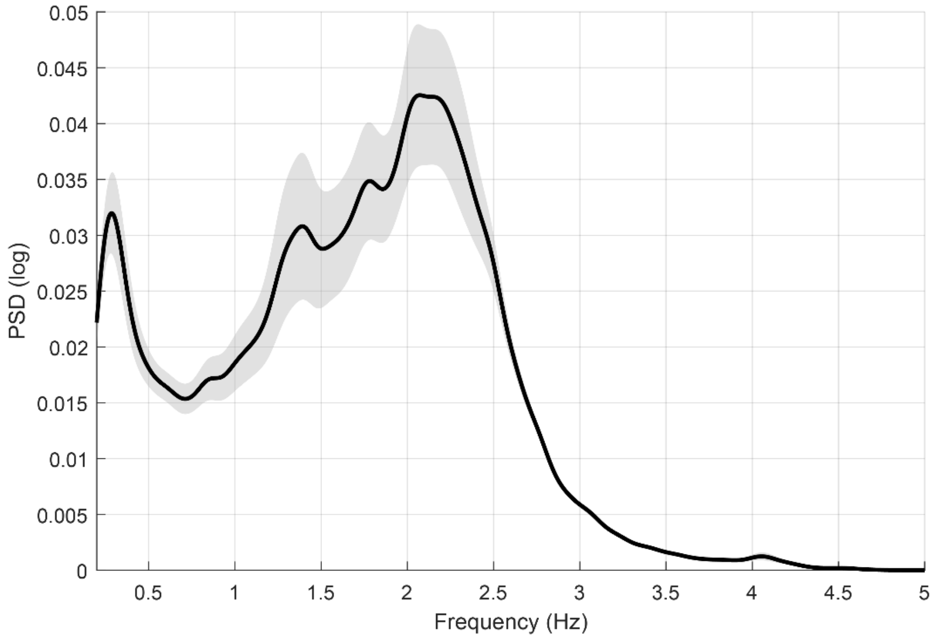


**Supplementary Figure S7**. Power spectral density (PSD; mean ± SEM) of the hemoglobin-regressed, band-pass filtered GCaMP signal used to visualize fast calcium transients in Fig. 1f, demonstrating structured power within the passband. Source data are provided as a Source Data file.

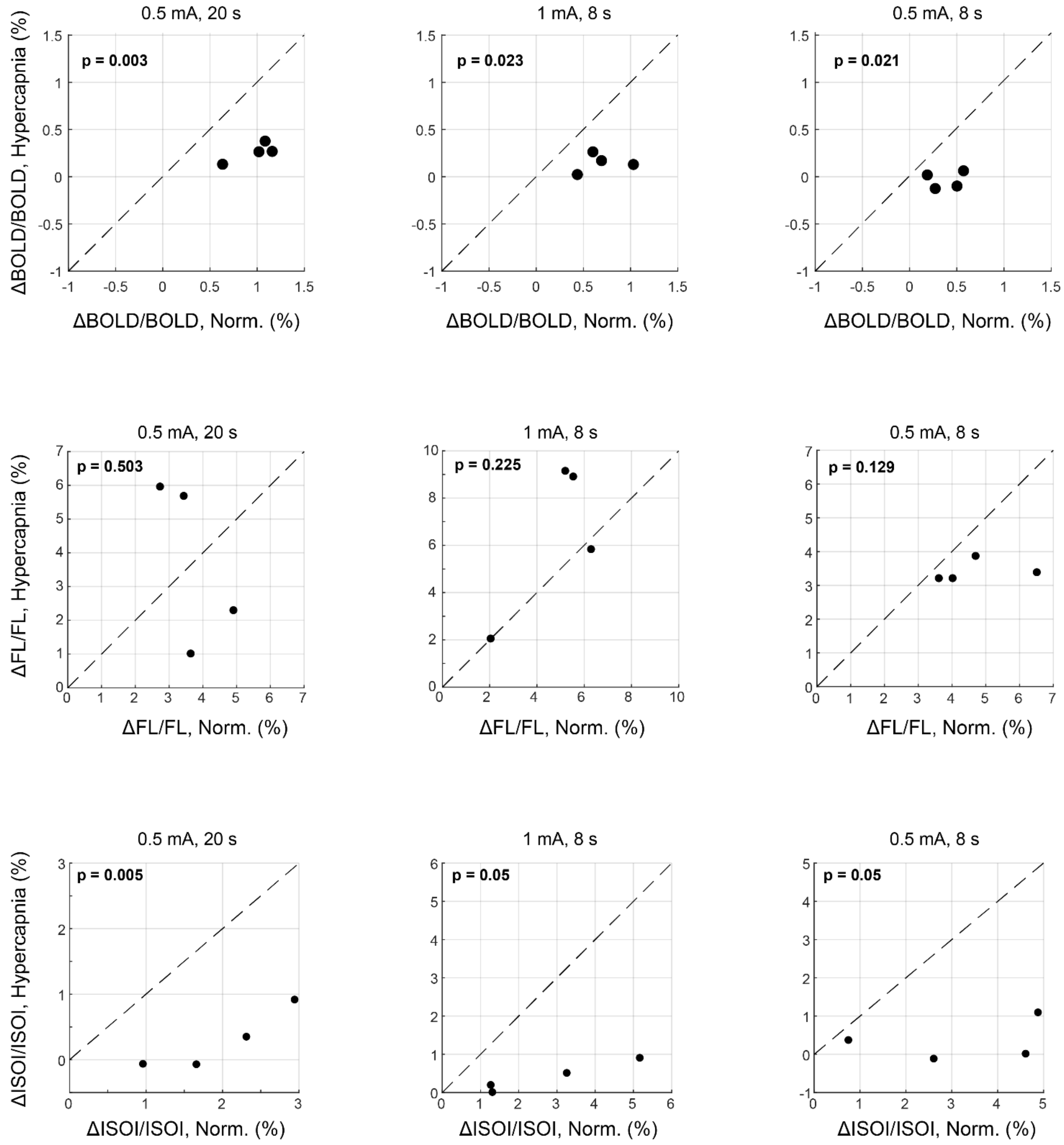


**Supplementary Figure S8**. Individual-animal BOLD (top row), FL (middle row), and ISOI/hemoglobin-weighted (bottom row) percentage signal changes to three forepaw stimulations (0.5 mA, 20 s, left; 1 mA, 8 s, middle; 0.5 mA, 8 s, right) under normocapnia and 5 % $CO_2$ hypercapnia.

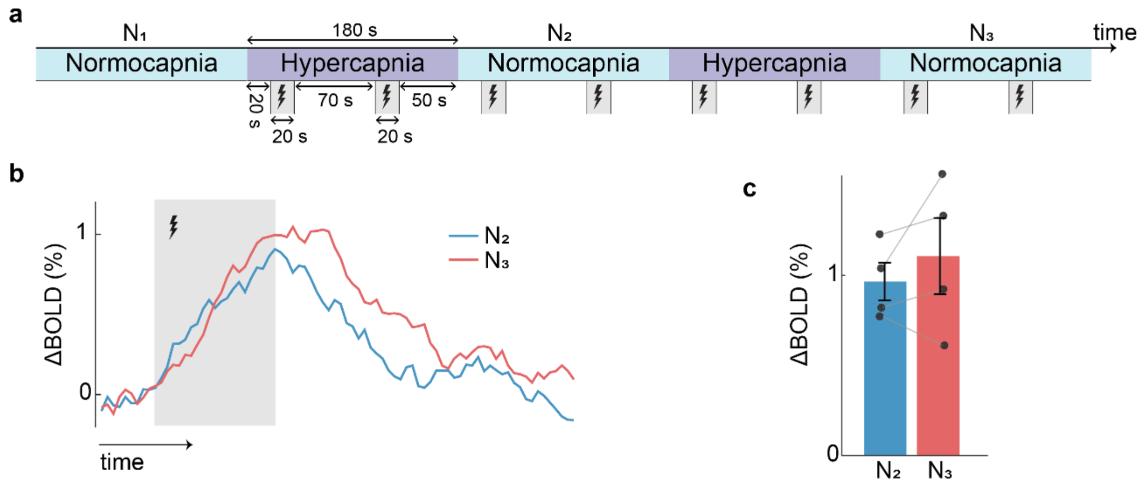


**Supplementary Figure S9**. **a** Timeline of the forepaw-stimulation paradigm across alternating breathing blocks. Electrical stimuli (0.5 mA, 20 s) began in the first hypercapnia block and were delivered 20 s after block onset. Two stimuli were given per block with 90 s onset-to-onset spacing. For the 8 s protocols, stimulus onsets were identical to the 20 s case. Consequently, the offset-to-next-onset interval increased from 70 s (20 s stimuli) to 82 s (8 s stimuli). **b** Group-averaged (N = 4) BOLD responses during the second and third normocapnia blocks (i.e., the first and second normocapnia blocks in which stimulation was delivered). **c** Peak BOLD increases to forepaw stimulation across successive normocapnia blocks show no amplitude decline, indicating no rundown with repeated stimulation.

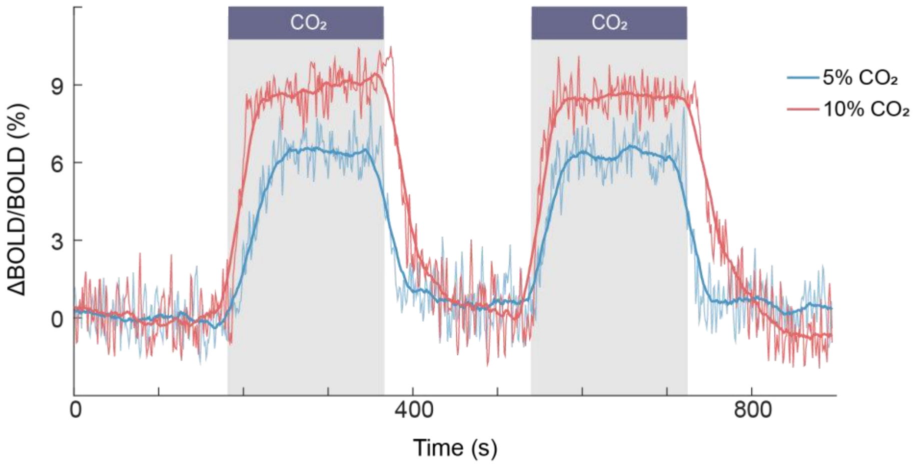


**Supplementary Figure S10.** BOLD signal time courses from the same mouse during inhalation of 5 % (blue) and 10 % (red) $CO_2$, plotted as percentage change relative to baseline normocapnia.

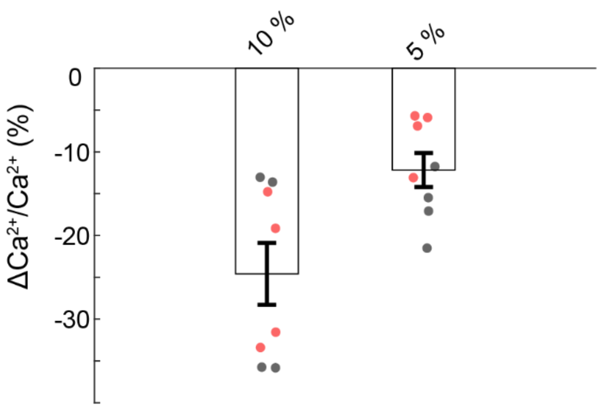


**Supplementary Fig. S11.** Anesthesia-independence of $Ca^{2+}$ transient amplitude reduction under hypercapnia. Red and black symbols denote ketamine–xylazine and isoflurane–medetomidine regimens, respectively. In both anesthetic conditions, $Ca^{2+}$ transient amplitudes are reduced to a similar extent during 5 % and 10 % $CO_2$ inhalation, demonstrating that the hypercapnia-induced attenuation of neuronal activity is independent of the anesthesia protocol.

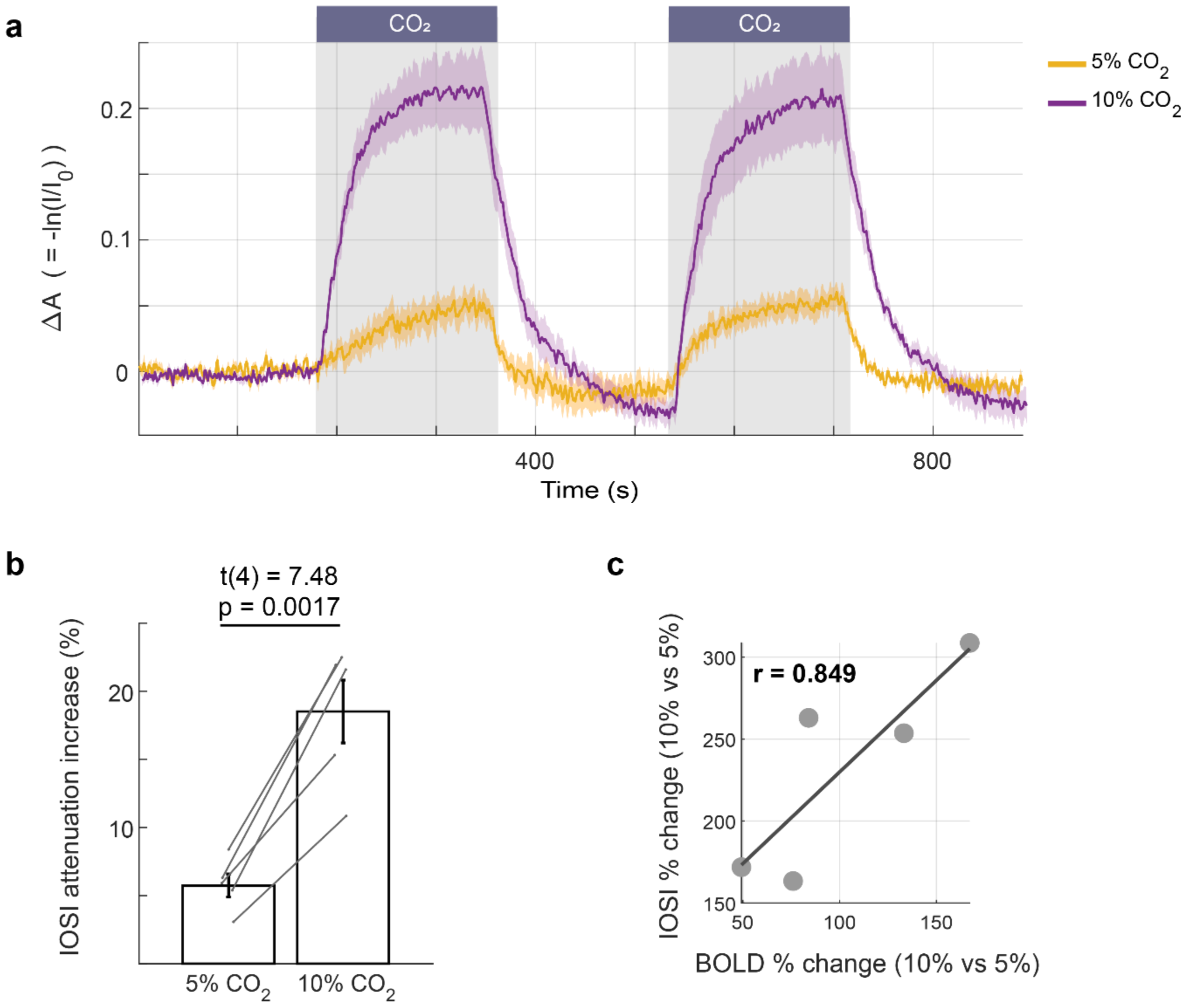


**Supplementary Fig. S12.** IOSI-derived absorbance and attenuation changes scale with increased hypercapnia and agree with BOLD. **a** Time-resolved absorbance changes $\Delta A(t)$ derived from the hemoglobin-weighted IOSI signal at 561 nm during 5% and 10% $CO_2$ inhalation (n = 5; mean ± SEM). Absorbance was computed from the Beer-Lambert relation using the baseline-normalized intensity $F(t) = I(t)/I_0$ as $\Delta A(t) = -\ln(I(t) / I_0) = -\ln(F(t))$. Because hemoglobin absorption dominates at 561 nm, a decrease in IOSI intensity corresponds to an increase in absorbance (consistent with increased hemoglobin content/attenuation). **b** The corresponding attenuation (percent-equivalent absorption increase) rose from 5.74 ± 0.85% at 5% $CO_2$ to 18.52 ± 2.31% at 10% $CO_2$ (paired t-test: $t(4) = 7.48$, $p = 0.0017$). Percent attenuation was computed from $\Delta A$ as % attenuation = $(1-e^{-\Delta A}) \times 100$, and per-animal values were obtained by averaging the two activation epochs within each $CO_2$ condition. **c** Across animals, the percent attenuation increase from IOSI (panel b) covaried with the corresponding BOLD percent increase when transitioning from 5% to 10% $CO_2$, yielding a Pearson correlation coefficient of $r = 0.849$. Source data are provided as a Source Data file.

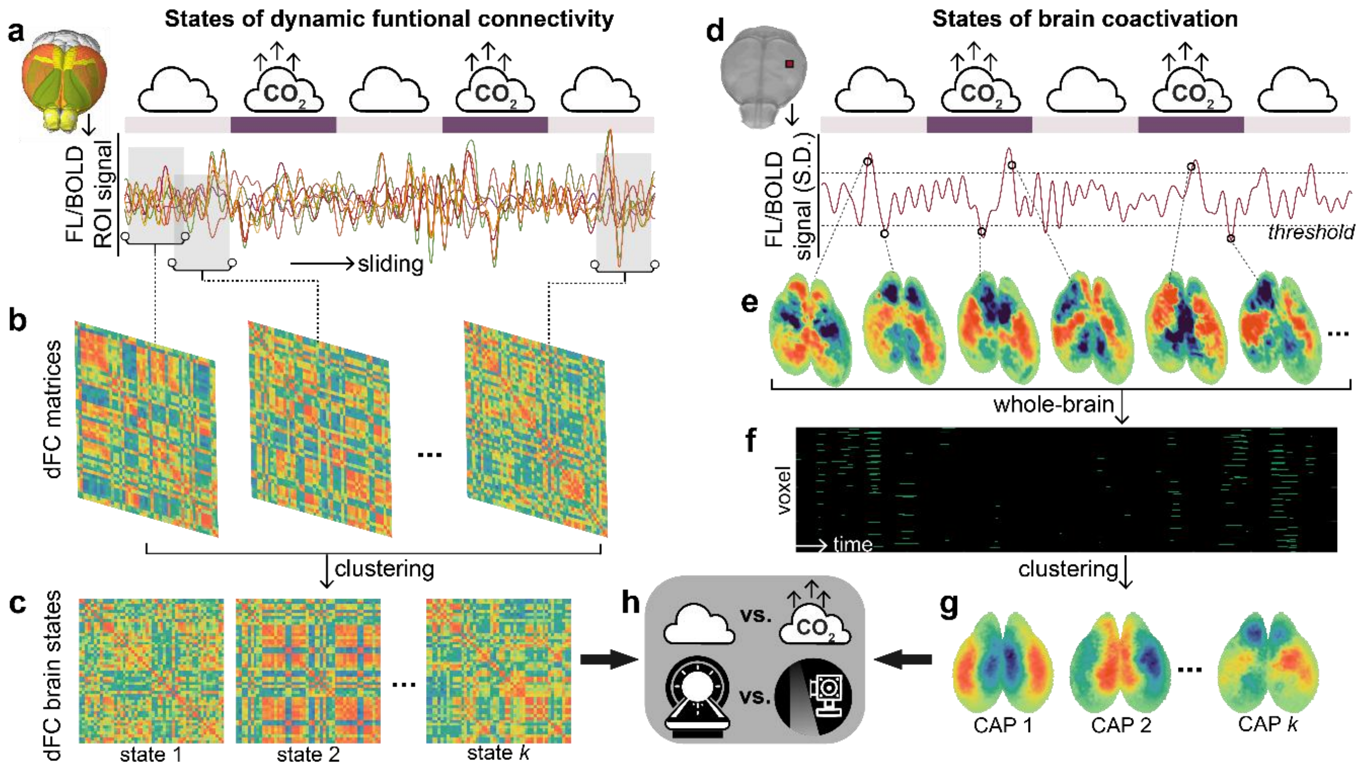


**Supplementary Figure S13. Workflow for multimodal brain state analysis using dFC and CAP analyses. a-c** The dFC analysis captures time-varying patterns of connectivity across functionally distinct brain regions. Mouse brain was parcellated according to the Allen mouse brain atlas, and the signals from the regions were used to compute the dFC matrices by calculating the correlation coefficient between each region pair (a). A sliding-window approach was applied (window length= 30s, window shift= 1s), resulting in 866 dFC matrices per animal (b). The dFC matrices were then clustered using a k-means algorithm to identify recurring dFC brain states (c). **d-g** States of brain coactivation were estimated using CAP analysis. For each subject, significant activations and deactivations at the voxel level were identified by comparing the time-point signal against subject-specific thresholds, defined as two standard deviations above or below the mean for both BOLD and FL data (d). Each time instant produced a spatial map indicating the intensity distribution of brain signals (e). A frame was classified as significant if at least 5% of the voxels exceeded the defined threshold at that instant. Green color indicates a suprathreshold signal at a time-instant from the corresponding voxel (f). Significant frames were then concatenated and clustered using a k-means algorithm, resulting in recurring spatial patterns of brain activity, or CAPs (g). The relationship between dFC brain states and CAPs were subsequently analyzed between FL and fMRI under normocapnia and hypercapnia conditions (h).

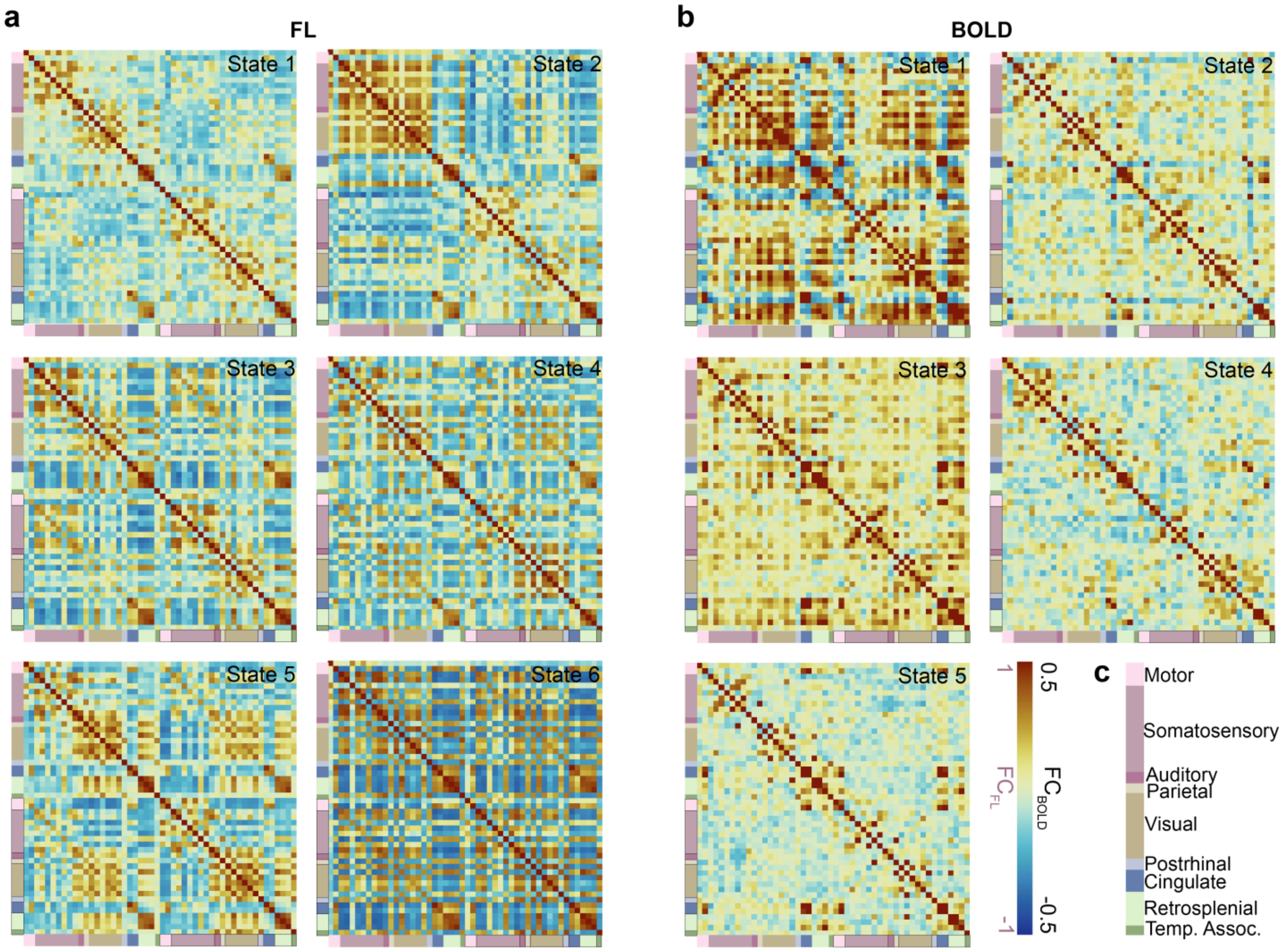


**Supplementary Figure S14.** Brain states identified of sliding-window dFC analysis. **a-b** Brain states identified by clustering the dFC matrices from FL (a) and BOLD (b) data. Each state reflects distinct patterns of functional connectivity across brain regions. **c** The brain networks represented in each row and column of the matrices are color-coded.

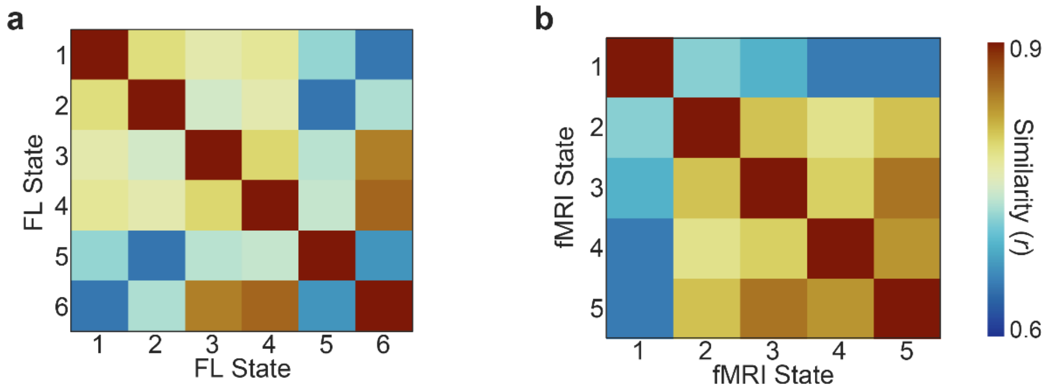


**Supplementary Figure S15.** Correlation across dFC state matrices for FL (a) and BOLD (b), illustrating the similarity between functional connectivity states in each modality. Source data are provided as a Source Data file.

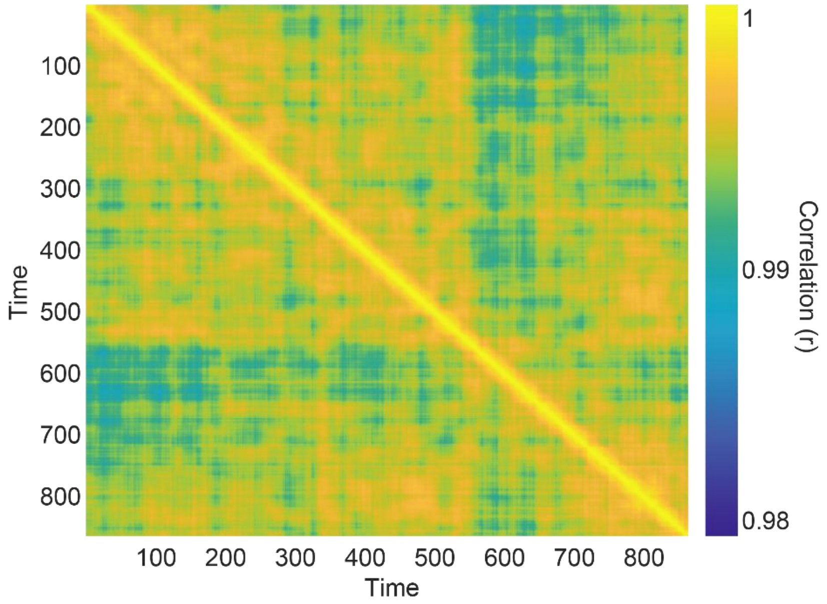


**Supplementary Figure S16.** The temporal consistency of the neural dFC matrices. The neural dFC matrices demonstrated high temporal consistency, with correlation coefficients calculated for each 30 s window (shifted by 1 s) consistently exceeding r > 0.99 across the entire time series.

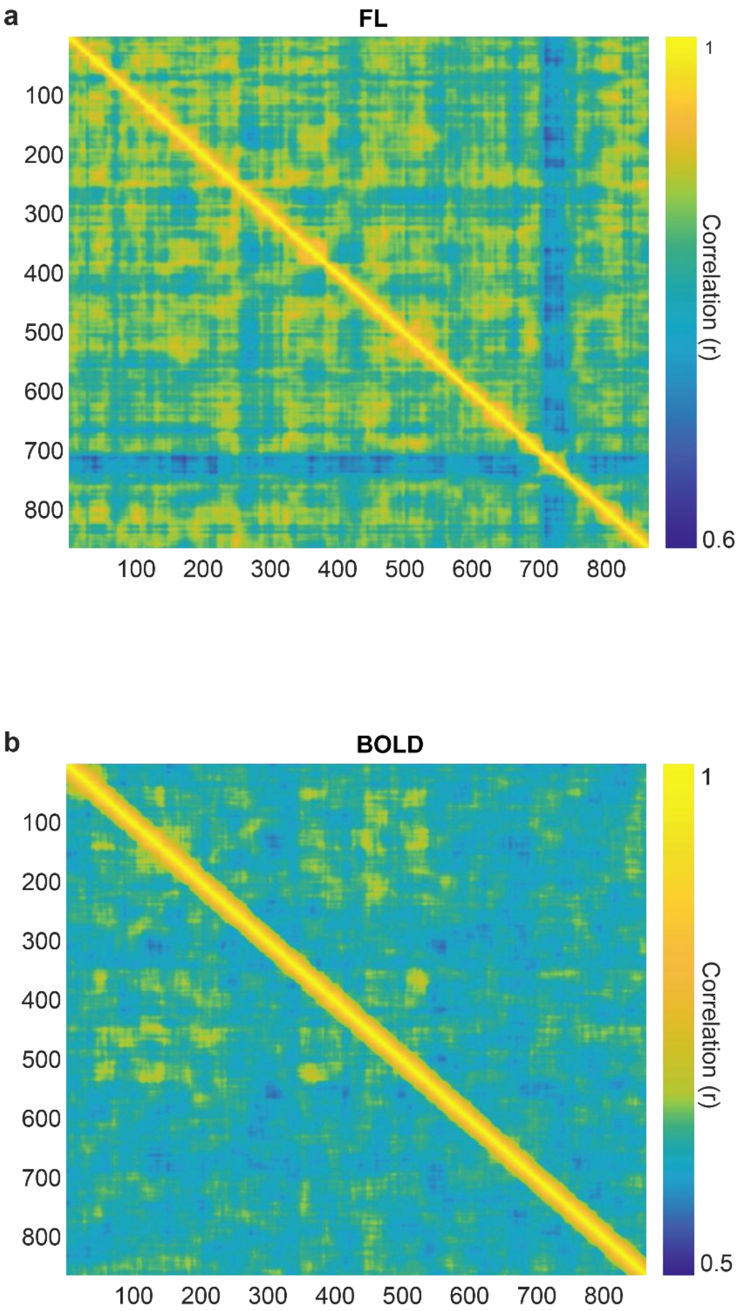


**Supplementary Figure S17.** Temporal consistency of FL (a) and BOLD (b) dFC matrices. Temporal consistency of dFC matrices for both FL (a) and BOLD (b) data, highlighting dynamic variability in functional connectivity across regions over time.

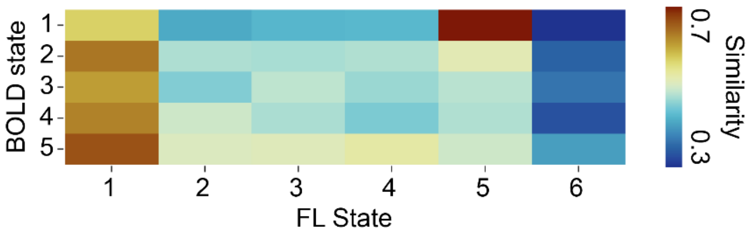


**Supplementary Figure S18.** The similarity between BOLD and FL dFC states. Source data are provided as a Source Data file.

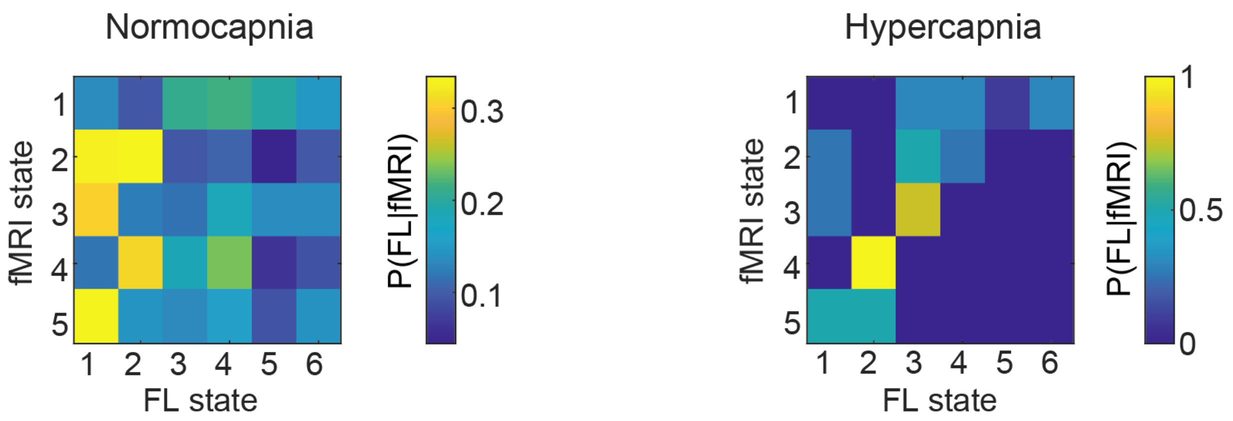


**Supplementary Figure S19**. Conditional co-occurrence probabilities of multimodal dynamic functional connectivity brain states under normocapnia and hypercapnia.

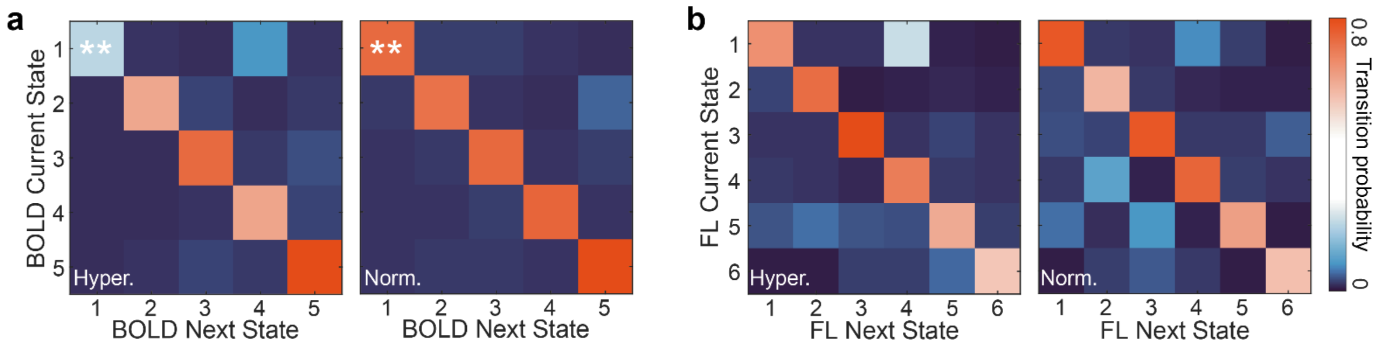


**Supplementary Figure S20.** Transition probabilities between BOLD (a) and FL (b) states. The self-transition probability of the neural-reflective BOLD state was reduced in hypercapnia, compared to normocapnia ($p = 0.01$, paired t-test).

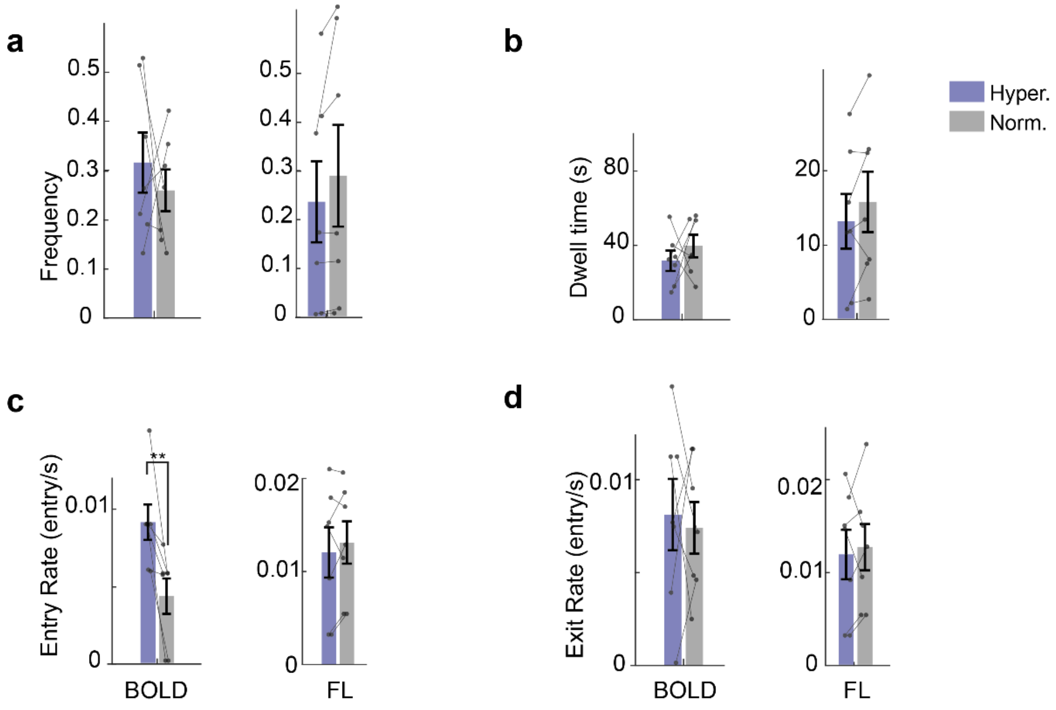


**Supplementary Figure S21.** Frequency (a), dwell time (b), entry rate (c), and exit rate (d) of BOLD and FL state representing the baseline brain activity during normocapnic and hypercapnic conditions. Source data are provided as a Source Data file.

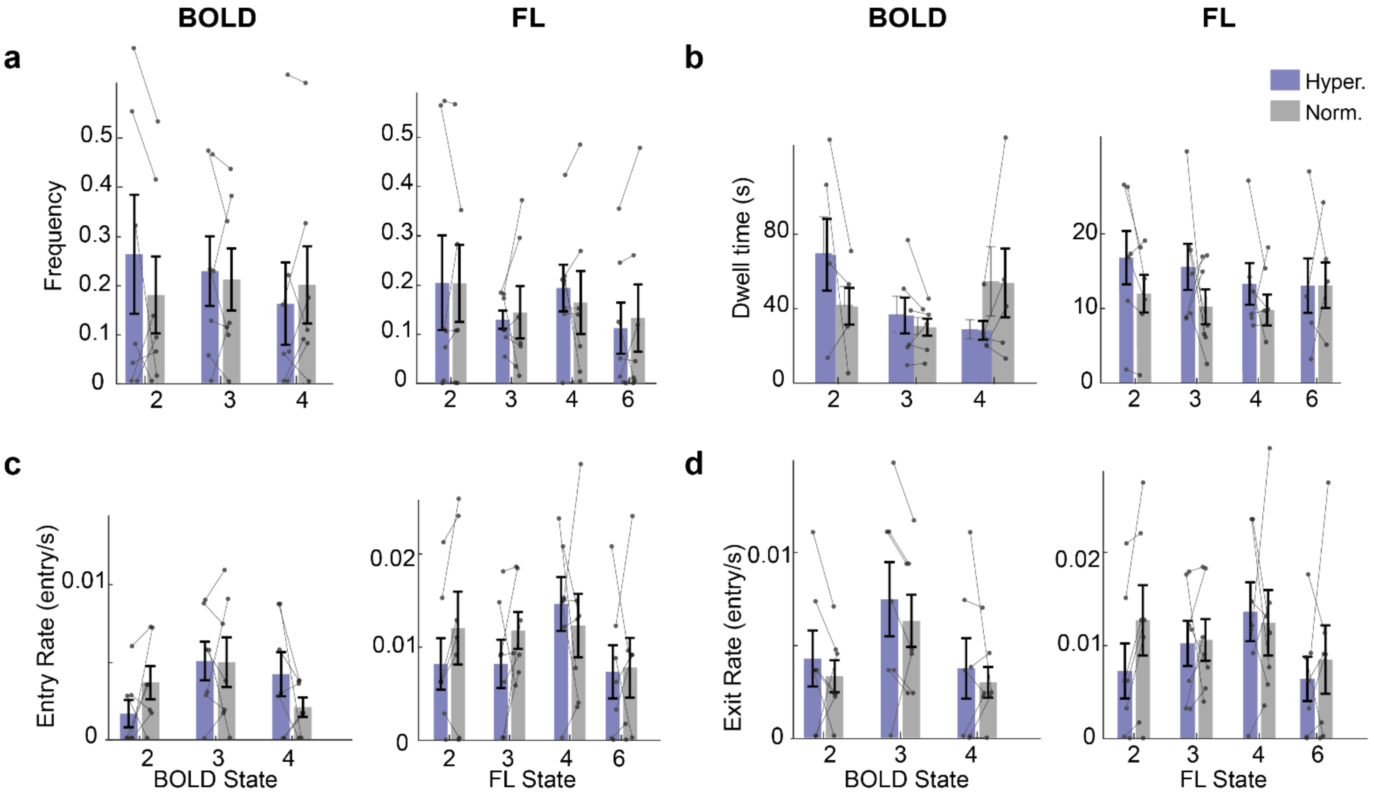


**Supplementary Figure S22.** Frequency (a), dwell time (b), entry rate (c), and exit rate (d) of the remaining BOLD and FL states during normocapnic and hypercapnic conditions. Source data are provided as a Source Data file.

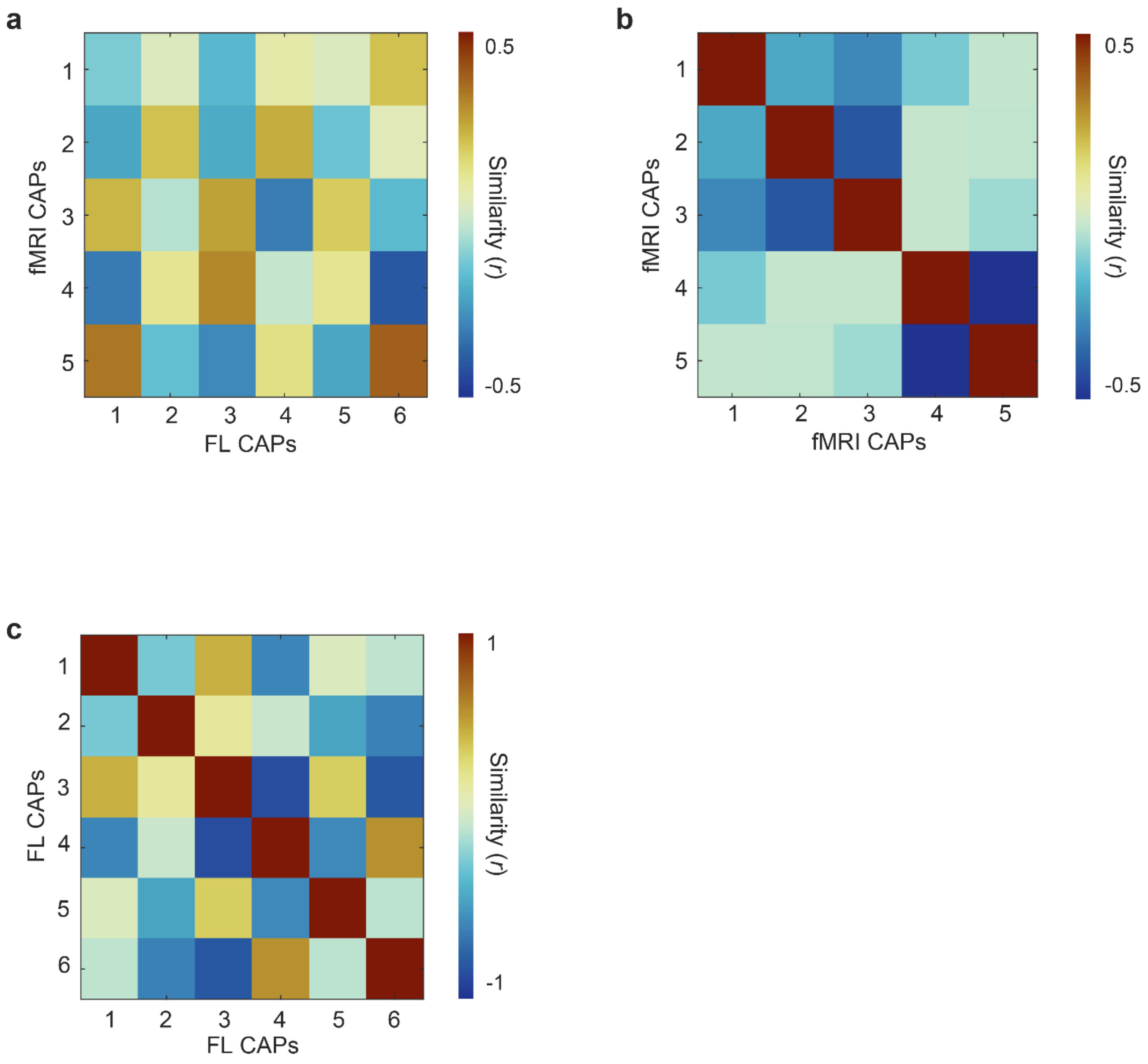


**Supplementary Figure S23. a-b** Correlation matrices for FL (a) and BOLD (b) CAPs, showing the similarity across spatial co-activation patterns within each modality. **c** Cross-modal similarity between FL and BOLD CAPs.

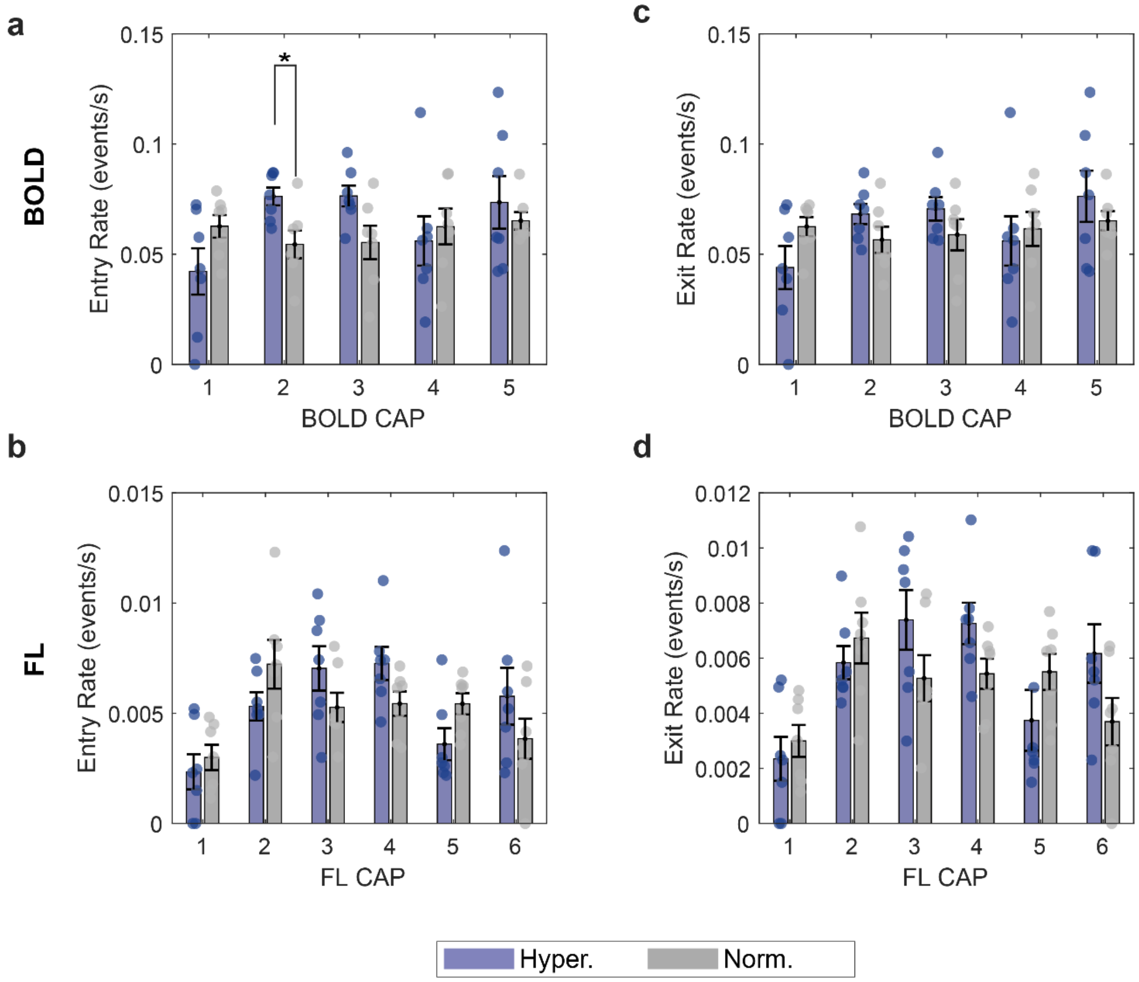


**Supplementary Figure S24. a-b** Entry rates for BOLD (a) and FL (b) CAPs, showing the frequency of state entry under normocapnic and hypercapnic conditions. **c-d** Exit rates for BOLD (c) and FL (d) CAPs. Source data are provided as a Source Data file.

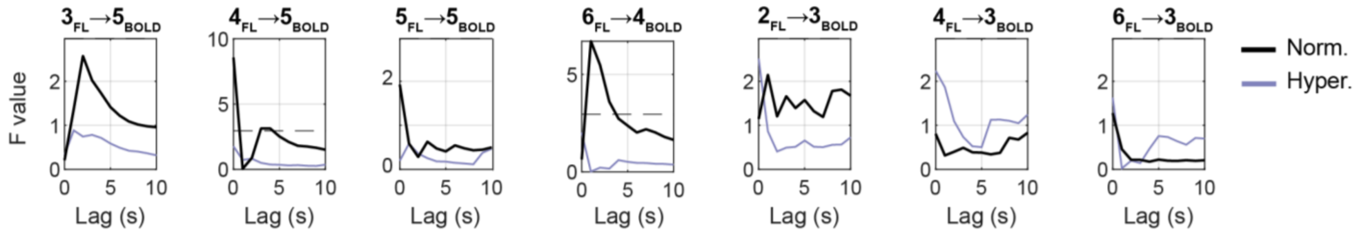


**Supplementary Figure S25.** Granger causality analysis of directional influences using downsampled FL CAPs. FL co-activation patterns were downsampled to the native BOLD sampling rate (1 s), and Granger causality were then computed at this temporal resolution.

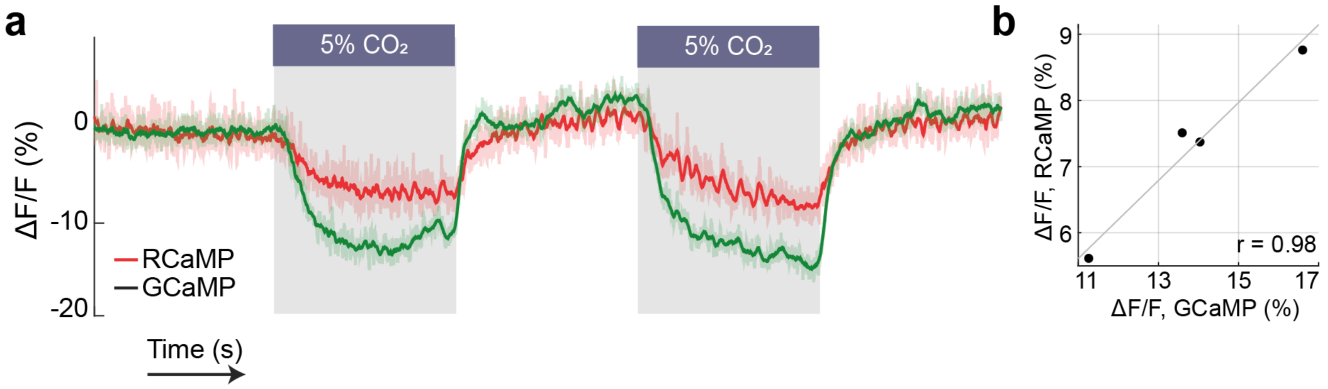


**Supplementary Figure S26.** **a** Raw RCaMP (neuronal) and GCaMP (astrocytic) responses to 5 % $CO_2$ hypercapnia challenge. **a** Representative time courses of neuronal (RCaMP1.07) and astrocytic (GCaMP6s) fluorescence during a 5 % $CO_2$ inhalation paradigm (n = 4). Traces are mean ± SEM. **b** Individual-animal percentage signal change for RCaMP1.07 and GCaMP6s during hypercapnia (n = 4), demonstrating a strong agreement between the two indicators (Pearson's r = 0.98). It is important to note that, in this dual-indicator cohort, no separate hemodynamic reference wavelength was recorded. Consequently, both channels are shown without hemoglobin regression and without pH normalization. During $CO_2$ blocks, the slow co-drifts largely reflect pH-dependent quenching of both indicators and hemoglobin absorption (stronger in the green band) and should not be interpreted as biological suppression of astrocytic or neuronal $Ca^{2+}$ activity. Similarly, the high correlation among the responses majorly indicates these shared optical influences.

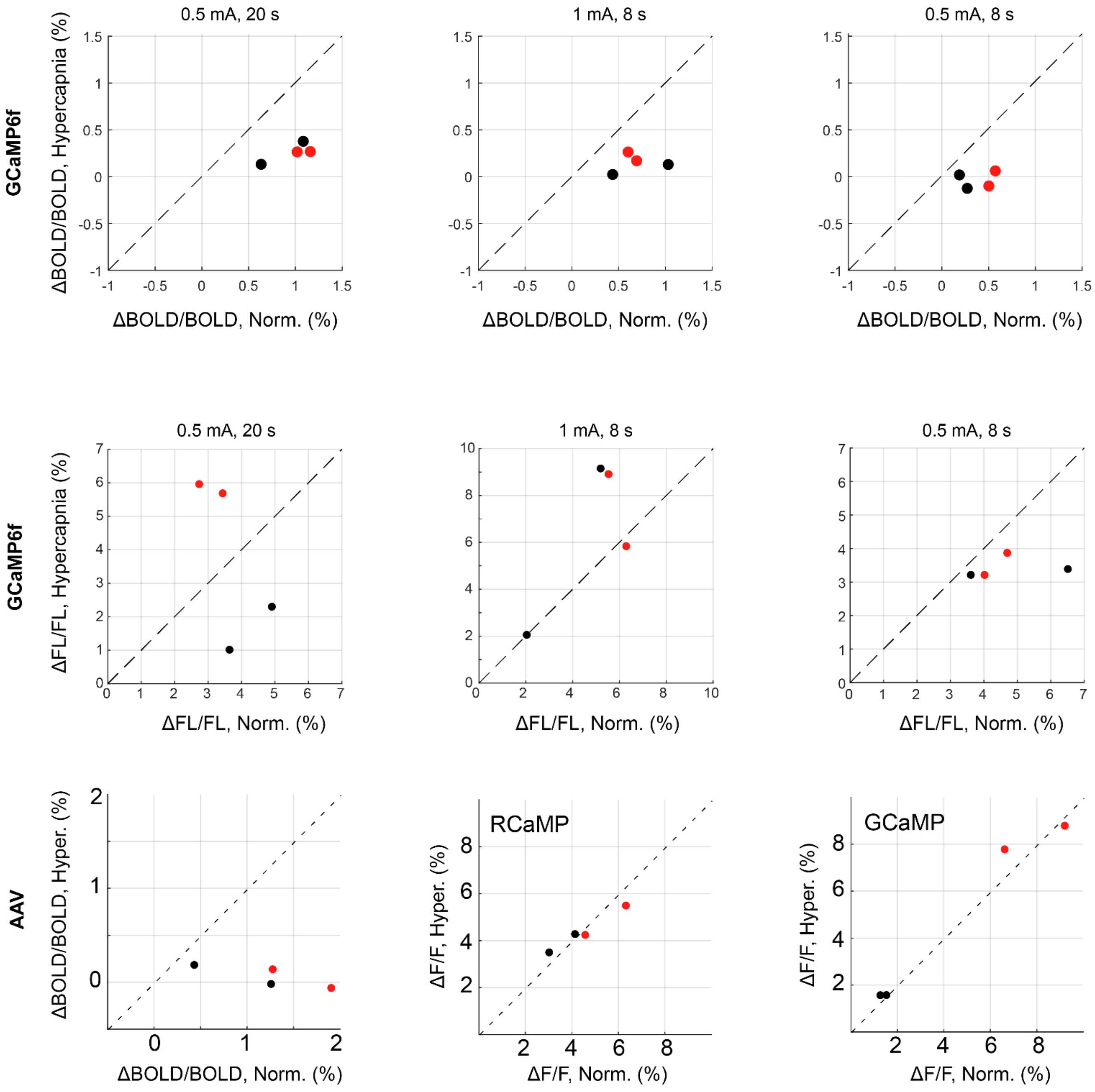


**Supplementary Fig. S27.** Anesthesia-independence of sensory-evoked FL and BOLD responses. Red and black symbols denote ketamine–xylazine and isoflurane–medetomidine regimens, respectively. Under both anesthesia protocols, forepaw stimulation evoked robust fluorescence signals in Thy1-GCaMP6f mice (top and middle rows) and in AAV-transfected mice (bottom row), during normocapnia and 5 % $CO_2$ hypercapnia. Conversely, hypercapnia consistently silenced BOLD responses in both mouse cohorts, regardless of anesthetic.

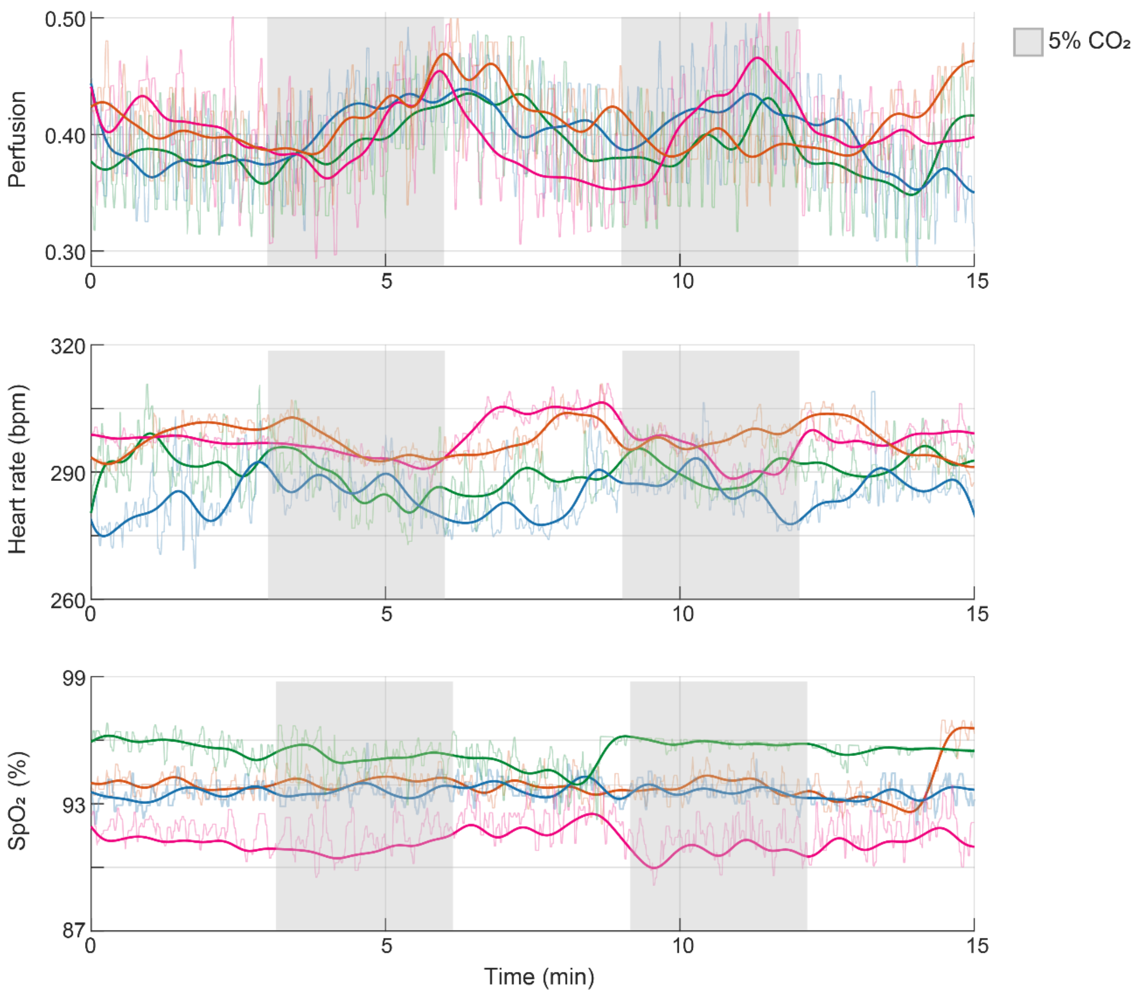


**Supplementary Fig. S28.** Physiological responses to an acute 5% $CO_2$ challenge in anesthetized GCaMP6f mice. Temporal profiles of perfusion index (top), heart rate (middle), and peripheral oxygen saturation ($SpO_2$; bottom) during a 3-minute 5% $CO_2$ challenge. Each trace corresponds to an individual animal. Hypercapnia produced a consistent increase in perfusion index, consistent with systemic vasodilation. Heart rate showed modest inter-animal variability, with small increases, decreases, or minimal change. $SpO_2$ remained largely stable throughout the challenge, consistent with compensatory increases in ventilation (hyperpnea) and maintained arterial oxygenation despite elevated inspired $CO_2$.

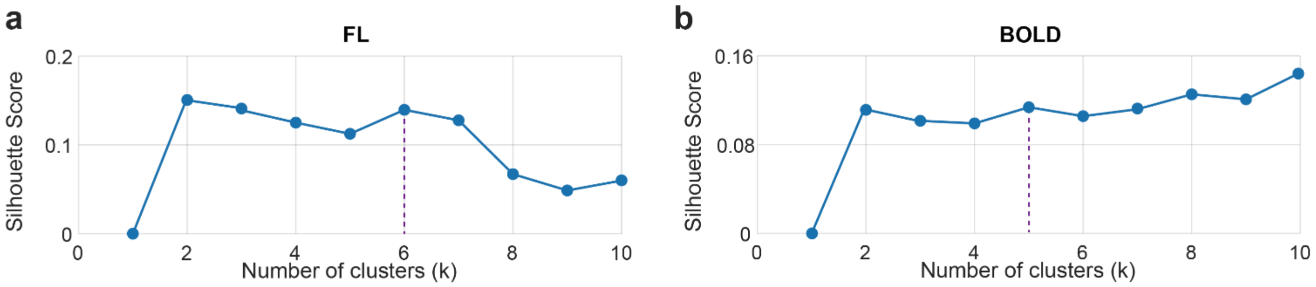


**Supplementary Fig. S29.** Silhouette scores for clustering dFC matrices of FL and BOLD data. **a** Silhouette scores for FL data, with the optimal number of clusters determined as $k$= 6 based on a local peak, ensuring sufficient detail over simpler clustering solutions (e.g., $k$= 2). **b** Silhouette scores for BOLD data, with $k$= 5 selected to balance local peak values and ensure that each state is consistently identifiable across at least 6 out of 7 subjects.

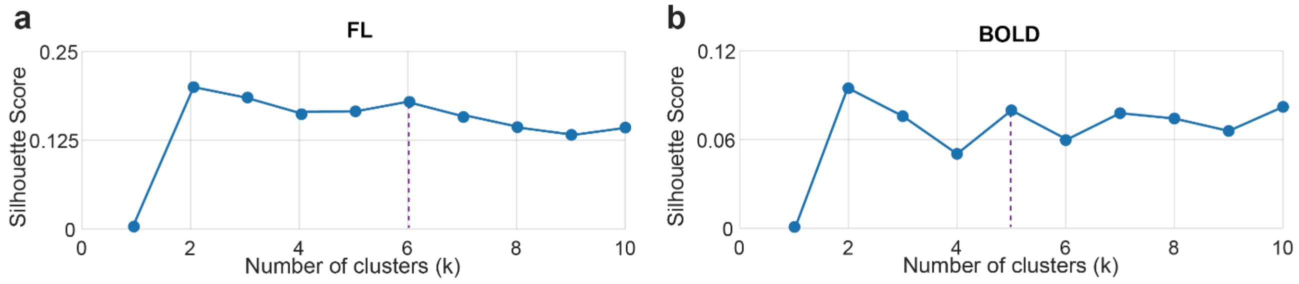


**Supplementary Fig. S30.** Silhouette scores for clustering CAP significant events of FL and BOLD data. **(a)** Silhouette scores for FL data, where the optimal clustering was determined as *k*= 6 based on a local peak, providing detailed structure over simpler models (e.g., *k*= 2). (b) Silhouette scores for BOLD data, with *k*= 5 chosen to optimize local peak scores and ensure each state is identifiable in at least 6 out of 7 subjects.

**Supplementary Table 1.** Full list of regions included in the FL and BOLD atlases employed in this study. Region definitions followed Allen mouse brain atlas.

| Cortical area | Region name |
|---|---|
| Motor areas | Primary motor area |
| | Secondary motor area |
| Primary somatosensory areas | Primary somatosensory area, nose |
| | Primary somatosensory area, barrel field |
| | Primary somatosensory area, lower limb |
| | Primary somatosensory area, mouth |
| | Primary somatosensory area, upper limb |
| | Primary somatosensory area, trunk |
| | Primary somatosensory area, unassigned |
| Supplementary somatosensory areas | Supplemental somatosensory area |
| Auditory areas | Primary auditory area |
| | Anterolateral visual area |
| | Anteromedial visual area |
| | Lateral visual area |
| Visual areas | Primary visual area |
| | Posteromedial visual area |
| | Rostrolateral area |
| Postrhinal areas | Postrhinal area |
| Anterior cingulate areas | Anterior cingulate area, dorsal part |
| Limbic areas | Prelimbic area |
| Retrosplenial areas | Retrosplenial area, lateral agranular part |
| | Retrosplenial area, dorsal part |
| | Retrosplenial area, ventral part |
| Anterior area | Anterior area |
| Association areas | Temporal association area |

**Supplementary Table 2.** Statistical measures characterizing the directional FL CAP-to-BOLD CAP relationship shown in Fig. 4f of the main manuscript. The table lists the peak time ($t_{peak}$), along with the corresponding F-statistic and p-value at that time, for both normocapnic and hypercapnic conditions. Directionality was assessed using a Granger causality analysis implemented as an F-test comparing nested autoregressive models. Reported p values are one-sided and uncorrected, derived from the F-distribution, with larger F values indicating stronger evidence for a directional influence.

| | Normocapnia | | | Hypercapnia | | |
|---|---|---|---|---|---|---|
| Direction | **$t_{peak}$ (s)** | **F value** | **p value** | **$t_{peak}$ (s)** | **F value** | **p value** |
| $3_{FL}$ to $5_{BOLD}$ | 0.3 | 6.76 | 0.001 | 0.3 | 2.86 | 0.058 |
| $4_{FL}$ to $5_{BOLD}$ | 0.3 | 13.32 | $p= 1.64 \times 10^{-6}$ | 0.6 | 1.20 | 0.920 |
| $5_{FL}$ to $5_{BOLD}$ | 0.3 | 5.34 | 0.005 | 0.4 | 3.95 | 0.008 |
| $2_{FL}$ to $3_{BOLD}$ | 0.8 | 1.59 | 0.130 | 0.3 | 3.75 | 0.024 |
| $4_{FL}$ to $3_{BOLD}$ | 1.4 | 1.64 | 0.070 | 0.6 | 3.23 | 0.006 |
| $6_{FL}$ to $3_{BOLD}$ | 0.6 | 4.84 | $p= 1.99 \times 10^{-4}$ | 0.5 | 3.78 | 0.011 |

**Supplementary Note 1.** pH normalisation of GCaMP6f signals under hypercapnia.

Inhaled $CO_2$ rapidly equilibrates with tissue bicarbonate, lowering both extracellular ($pH_o$) and intracellular pH ($pH_i$). Although acidification in either compartment may influence neuronal excitability, intracellular pH changes appear most critical [1, 2]. At the same time, the widely used GCaMP family of genetically encoded calcium indicators emits fluorescence exclusively from its deprotonated (anionic) chromophore when excited at ~488 nm. Thus, any drop in $pH_i$ will decrease the fraction of bright chromophores, quenching fluorescence and potentially mimicking a reduction in neural activity. In experiments employing 5% or 10% $CO_2$ challenges, it is therefore essential to distinguish true changes in calcium dynamics from pH-induced fluorescence artefacts. In this Note we first quantify the magnitude of this quench in GCaMP6f, while using GCaMP6m as a reference, and then derive a scale factor, S, that restores apparent $Ca^{2+}$ transients' amplitudes recorded under hypercapnia to their true values.

At 488 nm excitation, the per-molecule brightness of $Ca^{2+}$-bound GCaMP, $B(pH)$, is given by

$$B(pH) = \varepsilon\, \varphi\, f_{anion}(pH)$$

where ε is the molar extinction coefficient of the anionic chromophore, φ its fluorescence quantum yield (both pH-independent), and

$$f_{anion}(pH) = \frac{1}{1 + 10^{(pK_a - pH)}}$$

is the Henderson–Hasselbalch fraction of chromophores in the deprotonated (bright) state. The key photophysical parameters for $Ca^{2+}$-saturated GCaMP6m and GCaMP6f are summarized in Supplementary Table 1[3] .

Sustained exposure to 10 % $CO_2$ for 5 minutes has been reported to induce an intracellular acidification of ~0.18 pH units (from pH 7.29 to pH 7.12) [4]. Applying this ΔpH to the Henderson–Hasselbalch equation yields for GCaMP6f a 5.4 % decrease in the anionic fraction from 0.901 at pH 7.29 to 0.852 at pH 7.12. The corresponding values for GCaMP6m are from 0.715 to 0.613, yielding a 14.3 % reduction which also aligns with recently reported values[5]. Consequently, under an identical acidification, GCaMP6m fluorescence is quenched by approximately 2.6 times more than GCaMP6f.

**Supplementary Table 3.** Photophysical parameters of $Ca^{2+}$-bound GCaMP6 indicators[3].

| Sensor | $pK_{a,\,apo}$ | $pK_{a,\,sat}$ | $\varepsilon_{apo}$ (x1000) ($M^{-1}\,cm^{-1}$) | $\varepsilon_{sat}$ (x1000) ($M^{-1}\,cm^{-1}$) | $\Phi_{sat}$ |
|---|---|---|---|---|---|
| GCaMP6m | 8.68±0.09 | 6.90±0.04 | 2.07±0.04 | 38.2±0.9 | 0.61±0.01 |
| GCaMP6f | 8.77±0.16 | 6.34±0.01 | 2.80±0.03 | 61.9±0.4 | 0.590±0.002 |

We therefore define the scale factor for GCaMP6f under a 0.18-unit pH drop as

$$S_{6f} = \frac{0.901}{0.852} \approx 1.06,$$

such that multiplying recorded transient amplitudes during 10 % $CO_2$ by 1.06 restores them to their baseline values.

In this work, most resting-state protocols employ 5% $CO_2$, which induces a smaller intracellular acidification of <0.10 pH units [6]. Reapplying the same calculation for a $\Delta pH_i$ of 0.10, the anionic fraction of GCaMP6f falls from 0.901 at pH 7.30 to 0.879 at pH 7.20, a 2.4 % reduction. This yields to a correction factor $S_{6f}$ ≈1.025. Applying this factor to $Ca^{2+}$ transient amplitudes recorded under 5% $CO_2$ compensates for the pH-induced quench.

**Supplementary References**


1. Filosa JA, Dean JB, Putnam RW. Role of intracellular and extracellular pH in the chemosensitive response of rat locus coeruleus neurones. J Physiol. 2002;541(Pt 2):493-509.
2. Wang W, Bradley SR, Richerson GB. Quantification of the response of rat medullary raphe neurones to independent changes in pH(o) and P(CO2). J Physiol. 2002;540(Pt 3):951-70.
3. Chen T-W, Wardill TJ, Sun Y, Pulver SR, Renninger SL, Baohan A, et al. Ultrasensitive fluorescent proteins for imaging neuronal activity. Nature. 2013;499(7458):295-300.
4. Ritucci NA, Erlichman JS, Leiter JC, Putnam RW. Response of membrane potential and intracellular pH to hypercapnia in neurons and astrocytes from rat retrotrapezoid nucleus. Am J Physiol Regul Integr Comp Physiol. 2005;289(3):R851-61.
5. Barnett LM, Hughes TE, Drobizhev M. Deciphering the molecular mechanism responsible for GCaMP6m's Ca2+-dependent change in fluorescence. PLoS One. 2017;12(2):e0170934.
6. Nishimura M, Johnson DC, Hitzig BM, Okunieff P, Kazemi H. Effects of hypercapnia on brain pHi and phosphate metabolite regulation by 31P-NMR. J Appl Physiol (1985). 1989;66(5):2181-8.
