# Supplementary material for: Hypercapnia dissociates neuronal and hemodynamic responses impairing neurovascular coupling and functional brain connectivity
Source: Nat Commun. 2026 Apr 13;17:5158. doi: 10.1038/s41467-026-71742-z (PMC13249851; doi:10.1038/s41467-026-71742-z)
Supplement: Supplementary file 2 — Reporting Summary [file 41467_2026_71742_MOESM2_ESM.pdf]

nature portfolio

Corresponding author(s): Daniel Razansky

Last updated by author(s): Mar 18, 2026

Reporting Summary

Nature Portfolio wishes to improve the reproducibility of the work that we publish. This form provides structure for consistency and transparency in reporting. For further information on Nature Portfolio policies, see our Editorial Policies and the Editorial Policy Checklist.

Statistics

For all statistical analyses, confirm that the following items are present in the figure legend, table legend, main text, or Methods section.

n/a	Confirmed	
☐	☒	The exact sample size (*n*) for each experimental group/condition, given as a discrete number and unit of measurement
☐	☒	A statement on whether measurements were taken from distinct samples or whether the same sample was measured repeatedly
☐	☒	The statistical test(s) used AND whether they are one- or two-sided<br>*Only common tests should be described solely by name; describe more complex techniques in the Methods section.*
☒	☐	A description of all covariates tested
☐	☒	A description of any assumptions or corrections, such as tests of normality and adjustment for multiple comparisons
☐	☒	A full description of the statistical parameters including central tendency (e.g. means) or other basic estimates (e.g. regression coefficient) AND variation (e.g. standard deviation) or associated estimates of uncertainty (e.g. confidence intervals)
☐	☒	For null hypothesis testing, the test statistic (e.g. *F*, *t*, *r*) with confidence intervals, effect sizes, degrees of freedom and *P* value noted<br>*Give P values as exact values whenever suitable.*
☒	☐	For Bayesian analysis, information on the choice of priors and Markov chain Monte Carlo settings
☒	☐	For hierarchical and complex designs, identification of the appropriate level for tests and full reporting of outcomes
☐	☒	Estimates of effect sizes (e.g. Cohen's *d*, Pearson's *r*), indicating how they were calculated

Our web collection on statistics for biologists contains articles on many of the points above.

Software and code

Policy information about availability of computer code

Data collection	ParaVision 360 (6.0.1) for MRI acquisitions, Andor-SOLIS for fluorescence acquisitions.
Data analysis	MATLAB (R2023b), SPM12 for MRI and FL preprocessing and analysis. Examples of custom codes used in this manuscript are deposited and publicly available on Zenodo at https://doi.org/10.5281/zenodo.19099592.

For manuscripts utilizing custom algorithms or software that are central to the research but not yet described in published literature, software must be made available to editors and reviewers. We strongly encourage code deposition in a community repository (e.g. GitHub). See the Nature Portfolio guidelines for submitting code & software for further information.

Data

Policy information about availability of data

All manuscripts must include a data availability statement. This statement should provide the following information, where applicable:

- Accession codes, unique identifiers, or web links for publicly available datasets
- A description of any restrictions on data availability
- For clinical datasets or third party data, please ensure that the statement adheres to our policy

The main data supporting the finding of this study are available within the main text or Supplementary Information. Source data are provided with this paper. The raw multimodal datasets are too large to be publicly shared, yet they are available for research purposes from the corresponding author. Requests will be fulfilled within 4 weeks.

Research involving human participants, their data, or biological material

Policy information about studies with human participants or human data. See also policy information about sex, gender (identity/presentation), and sexual orientation and race, ethnicity and racism.

Reporting on sex and gender	The study did not involve human subjects.
Reporting on race, ethnicity, or other socially relevant groupings	The study did not involve human subjects.
Population characteristics	The study did not involve human subjects.
Recruitment	The study did not involve human subjects.
Ethics oversight	The study did not involve human subjects.

Note that full information on the approval of the study protocol must also be provided in the manuscript.

Field-specific reporting

Please select the one below that is the best fit for your research. If you are not sure, read the appropriate sections before making your selection.

- [x] Life sciences
- [ ] Behavioural & social sciences
- [ ] Ecological, evolutionary & environmental sciences

For a reference copy of the document with all sections, see nature.com/documents/nr-reporting-summary-flat.pdf

Life sciences study design

All studies must disclose on these points even when the disclosure is negative.

Sample size	No specific statistical methods or sample size estimations were used to define sample size. Sample sizes were determined based on available data that met the inclusion criteria.
Data exclusions	The assessment of resting-state dataset quality involved scrutinizing the simultaneous presence of robust inter-hemispheric connectivity in sensory cortices ($r > 0.1$), coupled with either weak connectivity or anti-correlation between sensory areas and anterior cingulate areas ($r < 0.1$). The datasets (one animal) not meeting this criterion were further excluded from the study.
Replication	All findings reported as conclusive were replicated in all animals investigated. The experiments were performed independently in each animal once
Randomization	This was an observational study without any intervention, and thus no randomization was applied.
Blinding	Data collection and analysis were not performed blinded. There were no experimental or control groups in the study. All animals underwent identical processing pipelines.

Reporting for specific materials, systems and methods

We require information from authors about some types of materials, experimental systems and methods used in many studies. Here, indicate whether each material, system or method listed is relevant to your study. If you are not sure if a list item applies to your research, read the appropriate section before selecting a response.

Materials & experimental systems

n/a	Involved in the study	
x		Antibodies
x		Eukaryotic cell lines
x		Palaeontology and archaeology
	x	Animals and other organisms
x		Clinical data
x		Dual use research of concern
x		Plants

Methods

n/a	Involved in the study	
x		ChIP-seq
x		Flow cytometry
	x	MRI-based neuroimaging

Animals and other research organisms

Policy information about studies involving animals; ARRIVE guidelines recommended for reporting animal research, and Sex and Gender in Research

Laboratory animals	In total 13 GCaMP6f mice (C57BL/6J-Tg (Thy1-GCaMP6f) GP5.17Dkim/J, Charles River Laboratories, Germany, 24–52 weeks old, n = 8, 7 female, 6 male) and and 4 C57BL/6 mice (Charles River Laboratories, 10 weeks-old, n = 4, female) were imaged in this study. Animals were housed in individually ventilated, temperature-controlled cages under a 12 h dark/light cycle. Pelleted food (3437PXL15, CARGILL) and water were provided ad-libitum.
Wild animals	The study did not involve wild animals.
Reporting on sex	7 female 4 male for GCaMP6f; 4 female for C57BL/6 mice.
Field-collected samples	The study did not involve samples collected from the field.
Ethics oversight	All animal experiments were performed in accordance with the Swiss Federal Act on Animal Protection and were approved by the Cantonal Veterinary Office Zurich (ZH060/22).

Note that full information on the approval of the study protocol must also be provided in the manuscript.

Plants

Seed stocks	*Report on the source of all seed stocks or other plant material used. If applicable, state the seed stock centre and catalogue number. If plant specimens were collected from the field, describe the collection location, date and sampling procedures.*
Novel plant genotypes	*Describe the methods by which all novel plant genotypes were produced. This includes those generated by transgenic approaches, gene editing, chemical/radiation-based mutagenesis and hybridization. For transgenic lines, describe the transformation method, the number of independent lines analyzed and the generation upon which experiments were performed. For gene-edited lines, describe the editor used, the endogenous sequence targeted for editing, the targeting guide RNA sequence (if applicable) and how the editor was applied.*
Authentication	*Describe any authentication procedures for each seed stock used or novel genotype generated. Describe any experiments used to assess the effect of a mutation and, where applicable, how potential secondary effects (e.g. second site T-DNA insertions, mosiacism, off-target gene editing) were examined.*

Magnetic resonance imaging

Experimental design

Design type	Block-design
Design specifications	3 min normocapnia + 2*(3 min hypercapnia + 3 min normocapnia). 900s in total.
Behavioral performance measures	No behavioral data was recorded during scanning.

Acquisition

Imaging type(s)	Functional, structural
Field strength	9.4T
Sequence & imaging parameters	T1-weighted: FLASH sequence, field of view (FOV) = 20×10 mm², matrix dimension (MD) = 160×80, 11 slices, slice thickness = 0.7 mm, repetition time (TR) = 500 ms, echo time (TE) = 2.1366 ms, and number of averages (NA) = 8.<br><br>Functional: gradient-echo echo-planar imaging (GE-EPI) sequence: FOV = 20×10 mm2, MD = 80×40, 11 slices, slice thickness = 0.7 mm, TR = 995 ms, TE = 12 ms, FA = 60°, temporal resolution = 1 s.
Area of acquisition	Whole-brain

Diffusion MRI ☐ Used ☒ Not used

Preprocessing

Preprocessing software	SPM12 (Wellcome Trust Centre for Neuroimaging, London, UK) were used for preprocessing. Datasets were corrected for motion (SPM realign function) and smoothened with a Gaussian kernel (FWHM 0.6×0.6×0.6 mm3).
Normalization	T1-weighted images were linearly normalized to the Allen mouse brain reference atlas. Following co-registration to structural scans, functional scans were normalized to the Allen mouse brain atlas by applying parameters from the T1-weighted images.
Normalization template	Allen Mouse Brain Common Coordinate Framework (CCFv3)

Noise and artifact removal	The six motion parameters (3 rotations, 3 translations), ventricle signals and vascular signals were removed from the detrended time-series with a general linear model. After nuisance regression, datasets underwent band-pass filtering (0.01-0.1 Hz).
Volume censoring	The first 5 volumes of the functional scans were discarded.

Statistical modeling & inference

Model type and settings	Group comparison for measures were performed as paired t-tests. Similarity between functional connectivity matrcies were examined by cross-correlation. Granger causality analysis was employed for directional models.
Effect(s) tested	We considered effects of hypercapnia on dynamic functional connectivity and brain coactivation patterns derived from multimodal fMRI and florescence recordings.

Specify type of analysis: ☐ Whole brain ☐ ROI-based ☒ Both

Anatomical location(s): ROIs were defined based on Allen mouse brain atlas.

Statistic type for inference (See Eklund et al. 2016)	Pearson correlation coefficients for ROI-based functional connectivity analysis.
Correction	Multiple comparisons corrected using the Benjamini-Hochberg method to control the false discovery rate (FDR) at a level of α=0.05.

Models & analysis

n/a	Involved in the study
☐	☒ Functional and/or effective connectivity
☒	☐ Graph analysis
☒	☐ Multivariate modeling or predictive analysis

Functional and/or effective connectivity	Pearson correlation (r)
