# Supplementary material for: Hypercapnia dissociates neuronal and hemodynamic responses impairing neurovascular coupling and functional brain connectivity
Source: Nat Commun. 2026 Apr 13;17:5158. doi: 10.1038/s41467-026-71742-z (PMC13249851; doi:10.1038/s41467-026-71742-z)
Supplement: Supplementary file 3 — Transparent Peer Review file [file 41467_2026_71742_MOESM3_ESM.pdf]

nature portfolio

Peer Review File

Hypercapnia dissociates neuronal and hemodynamic responses impairing neurovascular coupling and functional brain connectivity

Corresponding Author: Professor Daniel Razansky

This file contains all reviewer reports in order by version, followed by all author rebuttals in order by version.

Version 0:

Reviewer comments:

Reviewer #1

(Remarks to the Author)
Summary:
This study by Gezginer et al. employs a novel multimodal imaging approach combining fluorescence calcium imaging and functional MRI (fMRI) to investigate neurovascular coupling (NVC) under hypercapnic stress in mice. Using a knock-in mouse in which GCaMP6f is expressed in deep-layer pyramidal neurons, the authors simultaneously measured neuronal activity and hemodynamic responses to elevated inspired CO_2 levels. They observed a dissociation: while BOLD fMRI signals increased during hypercapnia (indicative of vasodilation), neuronal calcium signals concurrently decreased, suggesting suppressed excitability. Furthermore, both static and dynamic functional connectivity analyses revealed widespread desynchronization of brain networks. Co-activation pattern (CAP) analysis and Granger causality revealed that under hypercapnia, the typical temporal alignment between neural activity and hemodynamic responses was disrupted. Notably, while BOLD signals became less reflective of neural dynamics, calcium signals retained neural-specific patterns, highlighting hypercapnia's selective impact on vascular responsiveness. Thus, the work also shows that neural activity and BOLD fMRI signals do not always correspond with each other. Indeed, hypercapnia decouples neural and vascular signals, which is important for the interpretation of fMRI data.
However, the scope of the work here is quite limited for a Nature Communications paper. Essentially, the whole paper is only one experiment (though challenging and technically impressive) on 8 mice and a lot of analysis. There are no manipulations beyond CO2, such as sensory stimulation, pharmacology, or controls. Furthermore, the degree (%) or duration of CO2 is also not altered.
Nevertheless, the paper is timely considering the relatively recent Hosford and Tournissac papers on CO2 and neurovascular coupling. While the work as presented could potentially stand alone, there seems to be is a missed opportunity because the authors do not directly address the contradictory results between Hosford (primarily fMRI) and Tournissac (primarily fluorescence imaging). A key experiment in those previous papers is to determine weather or not functional and hypercapnic hyperemia occlude each other, with Hosford saying yes and Tournissac saying no. The authors here are in an ideal position to perform a similar experiment, expand the scope of their study and attempt to help settle this debate. I am hoping the authors will expand their study to increase my enthusiasm.

Major Comments:
Adding sensory stimulation of different intensities (and potentially durations) would be highly beneficial as the authors can more directly test the relationship between neural activity and hemodynamics with and without hypercapnia. Some of the authors conclusions regard this relationship but are based on resting state activity under anesthesia. It is still unclear if resting state NVC mechanisms are the same or different to sensory-based NVC, and the vast majority of NVC literature is sensory based. Nevertheless, I recognize there may be certain limitations with sensory stimulation in the authors unique experimental setup. While whisker or light stimulation would be ideal, if not, what else would be possible? This would be an excellent way to expand the study.

Is the loss of synchronization from hypercapnia and the decoupling of neural activity to the BOLD response due to a ceiling effect? i.e. once the vascular dilates to 3min of CO2, it cannot dilate much more to functional activation. This is critical to determine because all the network connectivity analysis is somewhat meaningless if the main cause is simply maximally dilated blood vessels. I suggest the authors try 10% CO2 as well and compare to the 5% data. Additionally, systemic

administration of a vasoconstrictor (such as indomethacin) and seeing the same relationships, would argue against a ceiling effect.

Anesthetic used was ketamine/xylazine which is not preferred for NVC studies. Ketamine blocks NMDA receptors, and NMDARs are a major regulator of NO and prostaglandin production in NVC. Xylazine is an alpha2 agonist which causes vascular smooth muscle contraction. While no anesthetic is ideal, it would be important to see if the main effects can be replicated with a different anesthetic.

The temporal resolution of fMRI is ~1s, whereas fluorescence calcium imaging ~100 ms, and this limits the direct alignment between neural and hemodynamic events. How was this dealt with regarding the CAP and Granger analysis? Loss of alignment is a major finding in the paper.

Most of the authors conclusions rest on correlation, clustering, and causality metrics (e.g., dFC, CAPs, Granger). These methods can be misleading if the underlying assumptions (stationarity, linearity, Gaussian distributions) are violated in health or in the hypercapnic brain. How was this controlled for? If no steps were performed, limitations need to be stated/discussed.

Was there any attention to higher frequency signals in the FL data that could be noise? The authors look at greater than 3Hz data. Are there non-neural signals much higher than this that should be filtered out? Spontaneous GCaMP signals beyond 15Hz cannot be faithfully detected due to slow off kinetics; thus, a bandpass filter in this range might be best.

Why do the authors speculate that data analysis finds 6 unique states in the FL data but only 5 in the BOLD data?

Line 117: The authors say "FL state 5 and BOLD state 1 showed the strongest correspondence to neural FC patterns". My simple question is: did these two states co-occur? Always? Sometimes?

Minor Comments:
The authors used Thy-1 mice which predominately express GCaMP6 in layer 5 pyramidal neurons, thus the authors are looking at dendritic signals primarily. This could be briefly discussed.

Fig2 appears to be purely an explanation of the dFC and CAP analysis. If there is no real data/result in Fig 2, the authors should consider moving this to the supplement.

Did the authors observed any sex differences? Both M and F mice were used (4 each) but don't recall that being mentioned in the text, though perhaps I missed it.

Line 34 should read: "…arachidonic acid metabolites…"

Line 42-43: should be revised for clarity, such as: However, opposite findings were reported recently, showing that hypercapnic hyperemia and functional hyperemia do not activate the same vasodilatory signaling pathways that mediate cerebrovascular responses

In the results upfront the authors should list the GCaMP mouse model they are using to help the reader understand the cell-type, location, depth of the measurements. Without this, the reader cannot understand or interpret the calcium signal measurement. It would also increase clarity if the authors could briefly mention the fluorescence recording technique too.

Line 86. Past tense is probably better. "First, we explored.." I believe there are other place too in the results like this the authors could correct.

Reviewer #2

(Remarks to the Author)
Gezginer et al. use state-of-the-art imaging techniques to revisit the interpretation of cerebrovascular reactivity to CO_2 in the field of BOLD fMRI. Under anaesthesia, they combine measurements of BOLD contrast and bulk GCaMP6 fluorescence signals in response to 5% CO_2. They report that CO_2 triggers a global drop of brain fluorescence and conclude that CO_2 generates a general decrease in cortical activity concomitant with a BOLD signal increase. While this observation appears to be irrefutable, I suspect that the interpretation is erroneous. It is well-known that CO_2 increases CBF and that haemoglobin absorbs light. Therefore, in the absence of correction for the fluorescence contamination by heamoglobin absorption (Ma et al. Philos. Trans. R. Soc. Lond. B Biol. Sci (2016), Scott et al. Neuron (2018)), the observed fluorescence decrease most probably reports a CBF increase. Furthermore, it is well-established that fluorescent reporters, organic dyes and fluorescent proteins, are sensitive to pH. By decreasing brain pH, CO_2 most certainly decreases GCaMP6 fluorescence, irrespective of the cell type expressing GCaMP6 (Tournissac et al. 2024). Therefore, it is likely that Gezginer et al. did not monitor a change in neuronal activity, but rather the combined side effects of heamoglobin absorption and GCaMP pH sensitivity. The study cannot be interpreted unless the authors demonstrate that GCaMP6 truly reports neuronal inhibition and not experimental side effects.

Reviewer #3

(Remarks to the Author)
This study by Gezginer and colleagues investigates neurovascular coupling by simultaneously measuring neuronal activity and hemodynamic responses using fluorescence calcium imaging and functional MRI (fMRI) in mice exposed to hypercapnia. The authors assess the relationship between neural calcium signals and blood oxygenation level-dependent (BOLD) responses, revealing distinct spatial and temporal patterns. The data reported show that hypercapnia induces a strong hemodynamic response, while neuronal activity is inhibited. This suggests a dissociation between vascular and neural activity, an observation which is important to consider when discussing the relationship between neuronal firing and fMRI signals. By integrating optical imaging with fMRI, the study provides valuable insight into cerebrovascular regulation and its implications for interpreting fMRI data. The study also contributes to an ongoing debate on the role of CO2 as a neuronal signal to the vasculature published in recent years in Nature Communications (PMID: 35440557; PMID: 39223128; PMID: 39223137). The findings support earlier observations (first observed by John Scott Haldane more than 100 years ago) that CO2 inhibits brain neuronal activity and can override NVC relationship. I have the following comments for authors to consider in their revision:

1. While the study clearly demonstrates a neurovascular dissociation, it does not delve into the underlying cellular or molecular mechanisms. Are astrocytes, endothelial cells, or pericytes mediating this effect? Further investigations using pharmacological or genetic tools would be needed to determine the mechanistic basis of these findings.

2. Calcium imaging, though precise, captures activity only in superficial cortical layers, whereas fMRI measures whole-brain hemodynamics, leading to potential spatial mismatches in the data. This needs to be discussed.

3. The authors should consider the pH sensitivity of the GCaMP sensor as fluorescence is reversibly quenching by acidic pH and may not accurately reflect the neuronal activity during hypercapnia.

Version 1:

Reviewer comments:

Reviewer #1

(Remarks to the Author)
The authors have done a great job addressing my concerns and expanding the manuscript. They study is more timely and impactful with this new data.

Major

I have one more major point regarding the new data: the authors need to ensure that the lack of NVC BOLD responses they measured in hypercapnia is not due to run down from successive forepaw stimulations with little time (90sec) between each, in which the system does not have adequate time to fully recover. As the authors are always starting with normocapnia, and then switching to hypercapnia, and giving two stimulations in each condition, run down could be an issue, and the authors do not show washout and recovery back in normocapnia. The exact order of all stimulations and conditions is not provide I believe. Note, the neural and astrocyte calcium response might not run down, but the vessel still could; thus, looking at the calcium data is not an adequate control per se. The authors have this data already (Fig S5) and they just need to look at the two responses given 90 sec apart and see if they are the same amplitude in normocapnia, or compare across different stimulations to determine if there is run down. If there is no run down, great, but if there is run down to the point that the current interpretation is invalid, they could repeat the experiment with a lot more time in between each forepaw stimulation (I recommend around 5 min), or show recovery of NVC by going back to normocapnia and repeating again.

Minor
I do not love the title of this manuscript. It is not very informative and it focuses more on the method than the finding. The authors could consider focusing on their outstanding method in the abstract and have title read something like this: Hypercapnia uncouples neuronal and hemodynamic responses impairing functional brain connectivity and neurovascular coupling. This is just a suggestion.

Line 39, the authors say “These vasoactive molecules engage various signaling pathways in vascular smooth muscle cells to induce vasodilation and increase cerebral blood flow (CBF) in response to neuronal activity.” Please add “and pericytes” after vascular smooth muscle cells.

Line 100 the authors say “Conversely, neuronal spike amplitudes decreased by 12.18 ± 2.04% at 5% CO_2 and 24.59 ± 3.70% at 10% CO_2 (Fig. 1I), confirming that hypercapnia directly attenuates neuronal excitability despite unaltered vascular responsiveness.” I do not understand the statement “unaltered vascular responsiveness”. I think the author’s are referring to the global increase in the BOLD signal to CO2 and not the impaired NVC which is discussed in this section. Perhaps the authors want to say: “despite unaltered global vascular responsiveness to inhaled CO2.”

Supplemental Fig 1 could also show the new 561 laser line. Or make the laser box more general.

Reviewer #2

(Remarks to the Author)
Gezginer et al. present new experiments that add useful information to the manuscript; however, the interpretation of several major observations remains questionable, if not invalid. Previous BOLD fMRI studies (e.g., Stefanovic et al., 2006; Gauthier et al., 2011; and others) have demonstrated the importance of quantifying both resting and evoked CBF to correctly interpret BOLD responses to hypercapnia. These studies should be cited and discussed, as they suggest other explanations for the disappearance of BOLD responses upon CO2. Can Gezginer's work contribute to resolving the ongoing controversy over the effects of hypercapnia on neuronal activity and neurovascular coupling (NVC)? Yes, but only if the authors, which possess all the necessary tools to address this question, perform the key controls which are still missing. First, the effect of CO2 on neuronal activity: bulk GCaMP6 fluorescence is insufficient to draw definitive conclusions, as GCaMP6 is pH-sensitive and thus a poor stand-alone reporter of activity. Corrections for pH sensitivity and hemoglobin absorption are only approximate and should be recalculated (see below). Direct measurements of neuronal activity—via multielectrode recordings or cellular-resolution calcium imaging—are required. A consistent reduction in firing rate or single-cell calcium transient frequency during CO_2 exposure would provide unambiguous evidence of reduced neuronal activity. The second important issue concerns the effect of CO_2 on NVC: Supplementary Figure S2 shows that the authors can measure hemodynamic responses independently of BOLD fMRI. CBV responses to hypercapnia should be recorded, and CBV traces added to Figure 1i, to directly assess NVC. Without these controls, the conclusions remain speculative.

Additional detailed comments:
1) In the manuscript, the definition of “spikes” is unclear. With the limited spatial and temporal resolution of the fluorescence acquisition system, the detected events cannot represent true neuronal action potentials. They are more likely spontaneous calcium fluctuations from neuron or astrocyte populations, depending on the expression patterns of GCaMP6 and RCaMP7.01. The term “spikes” should be avoided, as in neurons these signals could reflect synaptic potentials, action potentials, or second messenger–mediated calcium transients; in astrocytes, their meaning is even less certain. Moreover, the presentation of fluorescence data is inconsistent and prone to misinterpretation. In some cases, the authors appear to show the amplitude or envelope of Ca^{2+} “spikes,” while in others they display changes in baseline GCaMP6 fluorescence. Without a clear and consistent definition of what is being measured, most neuroscientists will find the results difficult to interpret.

2) The authors introduced a theoretical correction for the pH-dependent reduction of GCaMP6 fluorescence under hypercapnia. The pKa used is that of GCaMP6 in a saturated Ca^{2+} condition (Chen et al.), which corresponds to a situation with ~400 times more Ca^{2+} (39µM) than resting physiological condition (~100 nM). The 'apo' pKa (Ca^{2+}-free) - while not fully physiological—is closer to resting neuronal conditions. Using the pKa_apo of 8.77 would yield a scale factor S6f = 0.032/0.022 = 1.45 (instead of 1.06), dramatically increasing the correction required and highlighting the significant impact of acidosis on GCaMP6 fluorescence. Given the high uncertainty of the correction factor, neuronal activity should be corroborated using complementary techniques.

3) The authors report robust BOLD signals in subthalamic regions under hypercapnia, but Figure 1a suggests no cortical response—an unlikely scenario. Given that FL signals are acquired in superficial cortical layers, the authors must show CO_2 and NVC data from the same cortical regions where FL and BOLD signals were acquired, to ensure consistency.

4) Figure S22 (bottom left) shows conflicting results: the AAV-injected group (n=4) appears to show a 2% BOLD increase under hypercapnia following electrical stimulation—higher than their normocapnia response (0.4-1.9%). How to conciliate this with the other experiments?
5) Among the newly presented data, the authors report a marked decrease in astrocytic Ca^{2+} signal (or “spike” amplitude?) during hypercapnia, closely matching in magnitude and dynamic the decrease observed in neurons. In fact, Figure S21 shows a remarkably high correlation coefficient (r = 0.98) between neuronal (RCaMP1.07) and astrocytic (GCaMP6) signals in response to CO_2. While the authors attribute the neuronal GCaMP6 (or “spike amplitude”) decrease to inhibition, no explanation is provided for astrocytes. If not a pH-related artifact, what accounts for such a decrease in astrocytic Ca^{2+}? Has such a phenomenon been previously reported?

Reviewer #3

(Remarks to the Author)
In their revision, the authors substantially expanded the study with new experiments addressing the main critiques. They (1) performed sensory stimulation at multiple intensities and durations during both normocapnia and hypercapnia, showing that hypercapnia suppresses the BOLD response without reducing neuronal calcium transients, thus demonstrating that functional hyperemia is occluded downstream of neuronal activation; (2) added a 10 % CO_2 condition to test for vascular “ceiling effects,” finding further BOLD signals beyond the 5 % level and ruling out maximal dilation as the cause of neurovascular uncoupling; (3) repeated key tests under a different anesthetic regimen (isoflurane/medetomidine) and found consistent results; (4) implemented dual-wavelength optical recordings with hemoglobin signal regression and pH calibration to correct for optical artefacts and sensor quenching, confirming that hypercapnia genuinely reduces neuronal activity (well described in the earlier literature); and (5) incorporated cell-type–specific imaging (neurons and astrocytes). These additions address prior methodological concerns and strengthen the conclusions. Overall, their arguments appear well-supported: they convincingly show that the observed decoupling is not an artefact of hemodynamic contamination, pH effects, anesthesia choice, or vascular saturation.

Reviewer #4

(Remarks to the Author)
I co-reviewed this manuscript with one of the reviewers who provided the listed reports. This is part of the Nature Communications initiative to facilitate training in peer review and to provide appropriate recognition for Early Career Researchers who co-review manuscripts.

Version 2:

Reviewer comments:

Reviewer #1

(Remarks to the Author)
The authors have satisfactorily addressed all my comments.

Reviewer #2

(Remarks to the Author)
Gezginer et al. present some new data to answer our comments. Haemoglobin is known to have an oxygenation-dependent absorption spectrum in the visible and near-infrared ranges, a property that underlies intrinsic optical imaging. Typically, to monitor total blood flow with IOSI, one classically uses a high-contrast isosbestic point of the haemoglobin absorption spectra - a wavelength at which both HbO and HbR have the same absorption values. This makes the measurement independent of oxygenation and therefore sensitive only to changes in [HbT] (see Ma et al., Phil.Trans.R.Soc.B, 2016). We therefore do not understand why the authors used the single wavelength of 561 nm, which is precisely a wavelength at which both HbO and HbR vary, in opposite directions, thereby reducing any detectable changes in HbT and thus providing a poor measure of CBV. The authors now show, in Fig. 1k, small CBV responses to hind paw stimulation that decrease during hypercapnia. The point is that the CBV signal is not optimized (due to the wrong wavelength used), and since CO_2 modifies O_2 binding to haemoglobin (Bohr effect) and thus alters the HbO/HbR ratio, no reliable conclusion can be drawn from measurements at only 561 nm. Consequently, the conclusion that “the loss of the BOLD NVC response is accompanied by a loss of the CBV/HbT response” is incorrect. The issue of whether CBV responses accompany or not the “absence” of BOLD responses remains unresolved and crucial for the validity of the paper. The authors must use an appropriate laser wavelength to measure CBV changes during NVC as well as in response to the CO_2 challenge, which is still missing. This later straightforward experiment is essential because the authors regressed calcium transients using the "hemoglobin" signal (Fig. 1e,f).
Regarding the calcium spikes, which are now referred to as calcium transients, we remain in the dark about their nature. Could the authors at least show, in a supplementary figure, what they are referring to? We suggest simply expanding portions of the traces shown in Fig. 1e and f. This would help the reader understand the shape of these fluctuations. In their current presentation, they appear to represent some form of background noise (which amplitude could be modulated by pH). Are these fluctuations symmetrical? Is it possible to analyze their frequency? These transients seem to contaminate the data. For example, in the upper black trace in Fig. 1j, if one draws a line through the resting period (the first 5 seconds before stimulation), it appears that there is no fluorescence increase and possibly even a decrease after 10 seconds. Could the authors comment on this point? Clearly, more information is needed about these calcium “transients.”
Minor points:
- Please describe the IOSI methodology in the methods section.
- Please clearly indicate in Figure 1 and/or its legend the regions of interest (ROIs) from which the traces in panels i–m were extracted, in relation to panels a–b and g–h. We assume that panels i, j, and k correspond to the same ROI in the somatosensory cortex (as suggested by the heat maps in g–h), but this should be explicitly confirmed.
- The ketamine/xylazine anesthesia (100 mg/kg and 10 mg/kg, respectively) cannot be described as “light anesthesia” (lines 67 and 73), as these doses correspond to a surgical level of anesthesia, i.e. a deep anesthesia.
- References 11, 13, and 14 are not correctly cited in the introduction.
- Error bars are not consistently defined across figures; the only mention of SEM appears in line 418.
- Several axes are difficult to read due to missing or misaligned tick marks. Please check carefully (e.g. Fig.1m).
- Line 354: the phrase “8/20 s onset time, and 90 s stimulus repetition cycle” is unclear. Based on Figure 1, this likely refers to an 8 or 20 s stimulus duration rather than onset time.
- The Methods section states that respiration, heart rate, and SpO_2 were monitored, but no data are shown. These physiological parameters are important to interpret both spontaneous neural activity and baseline vascular tone and examples should be reported in a supplementary figure.
- In Figure 5, legends e and f appear inverted.

Reviewer #4

(Remarks to the Author)
I co-reviewed this manuscript with one of the reviewers who provided the listed reports. This is part of the Nature Communications initiative to facilitate training in peer review and to provide appropriate recognition for Early Career Researchers who co-review manuscripts.

Version 3:

Reviewer comments:

Reviewer #2

(Remarks to the Author)
Gezinger et al. have appropriately tempered the conclusions of some of their experiments. We understand that they do not wish to repeat their IOSI experiments using an appropriate laser wavelength, i.e., one at an isosbestic point of the hemoglobin absorption spectrum. Still, we have repeatedly asked the authors to present the IOSI data acquired at 561 nm during CO_2 stimulation. The authors possess the relevant data, and since the "pseudo CBV" signal is important to estimate the effect of hemoglobin on fluorescence signals, they should present the IOSI responses to CO_2 in a supplementary figure. If these responses mirror the dynamics of the BOLD responses shown in Supplementary Figure S10, then the use of the "CBV" signal could be considered acceptable. Otherwise and also in light of the effects of CO_2 on the BOLD signal (Kennerley et al., 2012), we continue to find the data difficult to interpret.

Reviewer #4

(Remarks to the Author)
I co-reviewed this manuscript with one of the reviewers who provided the listed reports. This is part of the Nature Communications initiative to facilitate training in peer review and to provide appropriate recognition for Early Career Researchers who co-review manuscripts.

Version 4:

Reviewer comments:

Reviewer #2

(Remarks to the Author)
The authors have responded to our final question.

Reviewer #4

(Remarks to the Author)
I co-reviewed this manuscript with one of the reviewers who provided the listed reports. This is part of the Nature Communications initiative to facilitate training in peer review and to provide appropriate recognition for Early Career Researchers who co-review manuscripts.

Open Access This Peer Review File is licensed under a Creative Commons Attribution 4.0 International License, which permits use, sharing, adaptation, distribution and reproduction in any medium or format, as long as you give appropriate

credit to the original author(s) and the source, provide a link to the Creative Commons license, and indicate if changes were made.
In cases where reviewers are anonymous, credit should be given to 'Anonymous Referee' and the source.
The images or other third party material in this Peer Review File are included in the article's Creative Commons license, unless indicated otherwise in a credit line to the material. If material is not included in the article's Creative Commons license and your intended use is not permitted by statutory regulation or exceeds the permitted use, you will need to obtain permission directly from the copyright holder.
To view a copy of this license, visit https://creativecommons.org/licenses/by/4.0/

Point by point responses to reviewers' comments

Reviewer #1:

This study by Gezginer et al. employs a novel multimodal imaging approach combining fluorescence calcium imaging and functional MRI (fMRI) to investigate neurovascular coupling (NVC) under hypercapnic stress in mice. Using a knock-in mouse in which GCaMP6f is expressed in deep-layer pyramidal neurons, the authors simultaneously measured neuronal activity and hemodynamic responses to elevated inspired CO_2 levels. They observed a dissociation: while BOLD fMRI signals increased during hypercapnia (indicative of vasodilation), neuronal calcium signals concurrently decreased, suggesting suppressed excitability. Furthermore, both static and dynamic functional connectivity analyses revealed widespread desynchronization of brain networks. Co-activation pattern (CAP) analysis and Granger causality revealed that under hypercapnia, the typical temporal alignment between neural activity and hemodynamic responses was disrupted. Notably, while BOLD signals became less reflective of neural dynamics, calcium signals retained neural-specific patterns, highlighting hypercapnia's selective impact on vascular responsiveness. Thus, the work also shows that neural activity and BOLD fMRI signals do not always correspond with each other. Indeed, hypercapnia decouples neural and vascular signals, which is important for the interpretation of fMRI data.

However, the scope of the work here is quite limited for a Nature Communications paper. Essentially, the whole paper is only one experiment (though challenging and technically impressive) on 8 mice and a lot of analysis. There are no manipulations beyond CO2, such as sensory stimulation, pharmacology, or controls. Furthermore, the degree (%) or duration of CO2 is also not altered. Nevertheless, the paper is timely considering the relatively recent Hosford and Tournissac papers on CO2 and neurovascular coupling. While the work as presented could potentially stand alone, there seems to be is a missed opportunity because the authors do not directly address the contradictory results between Hosford (primarily fMRI) and Tournissac (primarily fluorescence imaging). A key experiment in those previous papers is to determine weather or not functional and hypercapnic hyperemia occlude each other, with Hosford saying yes and Tournissac saying no. The authors here are in an ideal position to perform a similar experiment, expand the scope of their study and attempt to help settle this debate. I am hoping the authors will expand their study to increase my enthusiasm.

Reply: We thank the reviewer for highlighting the opportunity to extend the study. In our revised manuscript, we have broadened the experimental scope in three complementary ways. First, to directly test the open question of whether hypercapnia occludes functional hyperemia—a critical point of contention between prior fMRI and calcium imaging studies—we now present sensory stimulation experiments with three combinations of intensity and duration, delivered both during normocapnia and hypercapnia. This allows us to assess how hypercapnia affects stimulus-evoked neural and hemodynamic responses across a range of conditions, thus directly addressing the functional interplay between hypercapnia and sensory hyperemia. Second, to exclude a vascular-ceiling artefact we added a 10 % CO_2 condition alongside the original 5 % challenge. This new experiment was designed to systematically determine if increasing hypercapnic load results in a true upper limit of vascular response, or if neuronal and vascular signals become further decoupled with escalating CO_2. This approach provides a dose-dependent perspective on neurovascular coupling under stress. Third, given the ongoing debate about the cellular contributors to BOLD-calcium dissociation, we established a new mouse cohort in which RCaMP1.07 is expressed in neurons and GCaMP6f in astrocytes. This enables us to directly compare cell-type-specific calcium dynamics from both neurons and astrocytes under identical gas and sensory conditions, addressing the hypothesis that astrocytic activity may differentially contribute to neurovascular responses during hypercapnia.

We believe these additions extend the work beyond a single resting-state paradigm and hope to address the reviewer's points. Please find the point-by-point responses to the comments below.

Major Comments:

1) Adding sensory stimulation of different intensities (and potentially durations) would be highly beneficial as the authors can more directly test the relationship between neural activity and hemodynamics with and without hypercapnia. Some of the authors conclusions regard this relationship but are based on resting state activity under anesthesia. It is still unclear if resting state NVC mechanisms are the same or different to sensory-based NVC, and the vast majority of NVC literature is sensory based. Nevertheless, I recognize there may be certain limitations with sensory stimulation in

the authors unique experimental setup. While whisker or light stimulation would be ideal, if not, what else would be possible? This would be an excellent way to expand the study.

Reply: We agree that an evoked-response paradigm is the most direct way to probe neurovascular coupling under hypercapnia. Accordingly, in a new cohort of GCaMP6f mice ($n = 4$) we delivered contralateral electrical forepaw stimulation at three intensity/duration settings (0.5 mA × 8 s, 0.5 mA × 20 s, 1 mA × 8 s) during alternating normocapnia and 5 % CO_2 blocks. Robust calcium transients were observed in all animals under both gas conditions, whereas BOLD responses that were readily detectable in normocapnia were suppressed during hypercapnia. This demonstrates that the resting-state decoupling generalises to stimulus-evoked coupling and that hypercapnia functionally occludes the normal hyperemic response. The new data are shown in Fig. 1g-j, Supplementary Fig. S5, and detailed in the Results (lines 94-97).

We then repeated the 0.5 mA × 20 s protocol in a separate cohort ($n = 4$) co-expressing RCaMP1.07 in neurons and GCaMP6s in astrocytes within the upper-limb region of primary somatosensory cortex. Neuronal and astrocytic calcium responses were indistinguishable between normocapnia and hypercapnia, yet the BOLD response again vanished under 5 % CO_2 (Fig. 5). These results corroborate the GCaMP6f findings and indicate that astrocytic recruitment alone is insufficient to restore functional hyperemia once CO_2 is elevated. The new data are shown in Fig. 5 and detailed in the Results (lines 188–200) and discussed on lines 215–221.

2) Is the loss of synchronization from hypercapnia and the decoupling of neural activity to the BOLD response due to a ceiling effect? i.e. once the vascular dilates to 3min of CO2, it cannot dilate much more to functional activation. This is critical to determine because all the network connectivity analysis is somewhat meaningless if the main cause is simply maximally dilated blood vessels. I suggest the authors try 10% CO2 as well and compare to the 5% data. Additionally, systemic administration of a vasoconstrictor (such as indomethacin) and seeing the same relationships, would argue against a ceiling effect.

Reply: We thank the reviewer for this suggestion. We agree that it is essential to exclude the possibility that the neurovascular uncoupling we report merely reflects vessels that are already maximally dilated by 5 % CO_2. We therefore repeated the hypercapnia paradigm in an independent cohort of GCaMP6f mice ($n = 5$) in which each animal experienced both 5 % and 10 % CO_2. In the primary somatosensory cortex (upper-limb sub-region, the site used for all stimulus trials) the BOLD signal rose from 4.16 ± 0.62 % at 5 % CO_2 to 6.63 ± 0.66 % at 10 % CO_2 (paired $t = 9.77$, $p = 6 \times 10^{-4}$). These data demonstrate a substantial remaining vascular reserve at 5 % CO_2 and thus rule out a dilation ceiling as the cause of the observed loss of functional connectivity and calcium–BOLD synchrony. The new results are presented in Fig. 1k-l, Supplementary Fig. S6, and are described in the main text (lines 97–102).

3) Anesthetic used was ketamine/xylazine which is not preferred for NVC studies. Ketamine blocks NMDA receptors, and NMDARs are a major regulator of NO and prostaglandin production in NVC. Xylazine is an alpha2 agonist which causes vascular smooth muscle contraction. While no anesthetic is ideal, it would be important to see if the main effects can be replicated with a different anesthetic.

Reply: We appreciate the concern that ketamine/xylazine might bias neurovascular-coupling measurements. To verify that our key results are not regimen-specific we repeated the hypercapnia and sensory-stimulation paradigms in mice anaesthetised with isoflurane/medetomidine (Methods line 321-327). Across outcome measures, the two anaesthetics agreed closely. The reduction in calcium activity was similarly observed under both anesthesia protocols (Supplementary Fig. S7). Additionally, forepaw stimulation elicited robust calcium transients in both regimens, yet the BOLD response disappeared during hypercapnia in each case (new Supplementary Fig. S22). These results demonstrate that the neurovascular decoupling we report is robust to fundamentally different anaesthetic mechanisms. We have added the anesthesia comparison data in Supplementary Fig. S7 and S22 and note this cross-regimen consistency, as well as the remaining caveat that awake animals may behave differently, in the Discussion (lines 254–257).

4) The temporal resolution of fMRI is ~1s, whereas fluorescence calcium imaging ~100 ms, and this limits the direct alignment between neural and hemodynamic events. How was this dealt with regarding the CAP and Granger analysis? Loss of alignment is a major finding in the paper.

Reply: To align our CAP and Granger analyses, we up-sampled the BOLD event vector tenfold by replicating each 1 s sample across ten 100 ms bins to match the 100-ms resolution of the fluorescence data. This approach preserves the

high temporal fidelity of the calcium signals while ensuring valid frame-by-frame comparison. To verify that our key findings do not depend on this choice, in our revised manuscript, we downsampled the fluorescence event vector to 1 s bins and repeated the Granger causality tests (Supplementary Fig. S20). The same patterns of FL-BOLD directional influence under normocapnia and its loss under hypercapnia persisted, albeit with reduced F-values due to lower statistical power. The upsampling procedure is now detailed in the Methods for clarification (lines 467–468) and discussed (lines 229-231).

5) Most of the authors conclusions rest on correlation, clustering, and causality metrics (e.g., dFC, CAPs, Granger). These methods can be misleading if the underlying assumptions (stationarity, linearity, Gaussian distributions) are violated in health or in the hypercapnic brain. How was this controlled for? If no steps were performed, limitations need to be stated/discussed.

Reply: Although we detrended and band-pass filtered all time series to remove slow drifts and high-frequency noise (helping to approximate quasi-stationary, linear signals), we did not control for the assumptions. dFC and Granger-causality inherently assume that the underlying processes are at least locally stationary, linear, and approximately Gaussian, yet hypercapnia can indeed impose slow, non-linear vascular dynamics that violate these assumptions. Consequently, our connectivity and causality metrics primarily capture the dominant linear components of neurovascular coupling under CO_2 challenge. To acknowledge these caveats, we have revised our discussion (lines 231-233).

6) Was there any attention to higher frequency signals in the FL data that could be noise? The authors look at greater than 3Hz data. Are there non-neural signals much higher than this that should be filtered out? Spontaneous GCaMP signals beyond 15Hz cannot be faithfully detected due to slow off kinetics; thus, a bandpass filter in this range might be best.

Reply: Previously, we were relying on conservative filtering to ensure the least amount of contamination from hemoglobin-related dynamics. To eliminate hemoglobin-related confounds at the source, we now perform simultaneous dual-wavelength recordings (488 nm for GCaMP; 561 nm for hemoglobin contrast; Supplementary Fig. S2). The 561 nm channel is regressed from the 488 nm trace, removing first-order vascular absorption artifacts prior to any filtering. On these hemoglobin-corrected traces, we apply a 0.2–4 Hz band-pass filter (Supplementary Fig. S3). The 0.2 Hz high-pass cutoff removes slow drifts (e.g. photobleaching, laser-power fluctuations), while the 4 Hz low-pass cutoff excludes high-frequency noise (photon shot noise, electronic noise, mechanical vibrations) yet remains well below GCaMP6f's off-kinetics limit (~10–15 Hz) to preserve all physiologically relevant calcium transients. These enhancements are detailed in the revised manuscript (lines 86–93) and reflected in updated panels for Fig. 1e–f.

7) Why do the authors speculate that data analysis finds 6 unique states in the FL data but only 5 in the BOLD data?

Reply: We attribute the different state counts to fundamental contrasts in both the spatial and temporal information each modality preserves. Calcium imaging maintains localized neural fluctuations with high temporal fidelity, resolving rapid, subtle shifts in connectivity driven by dendritic events in GCaMP5.17 mice. In contrast, BOLD time-courses carry a dominant global or vascular component that is shared across regions and inherently low-pass–filtered by the haemodynamic response, smoothing over fast transitions. This shared variance and temporal blurring effectively lowers the dimensionality of the BOLD connectivity data, which may cause two finely distinct FL states to merge into a broader BOLD state during clustering.

8) Line 117: The authors say "FL state 5 and BOLD state 1 showed the strongest correspondence to neural FC patterns". My simple question is: did these two states co-occur? Always? Sometimes?

Reply: Both FL 5 and BOLD 1 are capturing the same core neural connectivity structure, but under very different constraints (fast vs. slow signals) and noise characteristics. The FL signal, being closer to the true neural dynamics, might reflect brief excursions into that connectivity state, whereas the BOLD signal (with its slower, smoothed nature) might gloss over or delay those excursions. Consequently, the two modalities only partially overlap in this state. We quantified this overlap as roughly 20% co-occurrence probability, a clear indication that FL 5 and BOLD 1 coincide occasionally rather than invariably. We have now reported these conditional co-occurrence probabilities (new Supplementary Fig. S14) and clarified this in the Results (lines 139–140).

Minor Comments:

1) The authors used Thy-1 mice which predominately express GCaMP6 in layer 5 pyramidal neurons, thus the authors are looking at dendritic signals primarily. This could be briefly discussed.

Reply: We thank the reviewer for this important point. For clarification, we have mentioned this in Results (line 76-79). We also acknowledged this in the Discussion (lines 257–259).

2) Fig2 appears to be purely an explanation of the dFC and CAP analysis. If there is no real data/result in Fig 2, the authors should consider moving this to the supplement.

Reply: The figure was moved to the supplementary file in the revised manuscript.

3) Did the authors observed any sex differences? Both M and F mice were used (4 each) but don't recall that being mentioned in the text, though perhaps I missed it.

Reply: We did not observe any sex differences. We have clearly indicated this in our revised manuscript (lines 254-255).

4) Line 34 should read: "…arachidonic acid metabolites…"

Reply: The sentence was corrected in the revised manuscript.

5) Line 42-43: should be revised for clarity, such as: However, opposite findings were reported recently, showing that hypercapnic hyperemia and functional hyperemia do not activate the same vasodilatory signaling pathways that mediate cerebrovascular responses

Reply: We thank the reviewer for pointing out this issue and the suggestion. The sentence was corrected in the revised manuscript (line 46-48).

6) In the results upfront the authors should list the GCaMP mouse model they are using to help the reader understand the cell-type, location, depth of the measurements. Without this, the reader cannot understand or interpret the calcium signal measurement. It would also increase clarity if the authors could briefly mention the fluorescence recording technique too.

Reply: We appreciate the suggestion and have added a description of the mouse model and optical read-out at the beginning of the Results section to orient the reader (now lines 76–79).

7) Line 86. Past tense is probably better. "First, we explored.." I believe there are other place too in the results like this the authors could correct.

Reply: The sentences with this issue were corrected in the revised manuscript.

Reviewer #2:

Gezginer et al. use state-of-the-art imaging techniques to revisit the interpretation of cerebrovascular reactivity to CO2 in the field of BOLD fMRI. Under anaesthesia, they combine measurements of BOLD contrast and bulk GCaMP6 fluorescence signals in response to 5% CO2. They report that CO2 triggers a global drop of brain fluorescence and conclude that CO2 generates a general decrease in cortical activity concomitant with a BOLD signal increase. While this observation appears to be irrefutable, I suspect that the interpretation is erroneous. It is well-known that CO2 increases CBF and that haemoglobin absorbs light. Therefore, in the absence of correction for the fluorescence contamination by heamoglobin absorption (Ma et al. Philos. Trans. R. Soc. Lond. B Biol. Sci (2016), Scott et al. Neuron (2018)), the observed fluorescence decrease most probably reports a CBF increase. Furthermore, it is well-established that fluorescent reporters, organic dyes and fluorescent proteins, are sensitive to pH. By decreasing brain pH, CO2 most certainly decreases GCaMP6 fluorescence, irrespective of the cell type expressing GCaMP6 (Tournissac et al. 2024). Therefore, it is likely that Gezginer et al. did not monitor a change in neuronal activity, but rather the combined side effects of heamoglobin absorption and GCaMP pH sensitivity. The study cannot be interpreted unless the authors demonstrate that GCaMP6 truly reports neuronal inhibition and not experimental side effects.

Reply: We appreciate the reviewer's insightful concerns regarding the pH sensitivity of GCaMP and potential contamination of our signals by hemoglobin absorption under hypercapnia. To ensure that our fluorescence

measurements faithfully report neuronal calcium dynamics rather than artifactual quenching or hemodynamic effects, we have implemented a suite of experimental and analytical refinements.

First, recognizing that CO_2-driven increases in cerebral blood flow alter fluorescence via hemoglobin absorption, we upgraded our imaging platform to a dual-wavelength approach (Supplementary Fig. S2). By simultaneous excitation at 488 nm (GCaMP) and 561 nm (hemoglobin contrast), we capture simultaneous calcium- and blood-volume–sensitive signals. Following coregistration, we regress the 561 nm–derived hemodynamic component from the 488 nm trace and apply a 0.2–4 Hz band-pass filter to remove slow drifts and high-frequency noise. This pipeline produces an Hb-corrected GCaMP6f signal in which residual fluctuations predominantly reflect intracellular calcium spikes (Fig. 1e-f, Supplementary Fig. S3).

Second, we quantified the degree of GCaMP6f fluorescence quenching induced by intracellular acidification in response to 5 % and 10 % CO_2 exposure, leveraging GCaMP6f pH-sensitivity curves (Supplementary Note 1). From these, we derived correction factors of 1.025 for 5 % CO_2 and 1.06 for 10 % CO_2. When applied to the measured spike amplitudes during hypercapnic periods, these factors restore the true underlying calcium transient magnitudes.

In line with these improvements, we performed new experiments in GCaMP6f mice (n = 5) under both 5 % and 10 % CO_2 challenges. Uncorrected spike amplitudes declined by approximately 13.9 % during 5 % CO_2 and 27.6 % during 10 % CO_2; after applying the appropriate scale factors, these reductions attenuated to 12.1 % and 24.6 %, respectively (Fig. 1k–l, Supplementary Fig. S4). Importantly, even when conservatively applying the larger 10 % CO_2 correction factor to the 5 % data, a significant 9.6 % decrease in amplitude persisted, demonstrating that mild hypercapnia genuinely suppresses neuronal calcium activity (Supplementary Fig. S4).

We also note that the extreme acidification required to explain the observed 27 % fluorescence drop (intracellular pH ~6.6) or the 14 % drop (pH ~6.9) far exceeds the pH shifts measured with up to 20 % inspired CO_2 for 10 min [1]. Thus, quenching cannot account for our observed residual amplitude reductions, which we confidently attribute to true decreases in neuronal calcium signaling. Taken together, these quantitative calibrations, artifact-regression methods, and new in vivo data assure us that our fluorescence measurements accurately reflect hypercapnia-induced neuronal inhibition rather than sensor artifacts.

The new results and improvements are given in Fig. 1e-f, Fig. 1l, Supplementary Fig. S2-S4, Supplementary Note 1, and detailed in lines 86-93 and 204-206.

[1] Pospelov AS, Puskarjov M, Kaila K, Voipio J. Endogenous brain-sparing responses in brain pH and PO2 in a rodent model of birth asphyxia. Acta Physiol (Oxf). 2020;229(3):e13467. doi:10.1111/apha.13467

Reviewer #3:

This study by Gezginer and colleagues investigates neurovascular coupling by simultaneously measuring neuronal activity and hemodynamic responses using fluorescence calcium imaging and functional MRI (fMRI) in mice exposed to hypercapnia. The authors assess the relationship between neural calcium signals and blood oxygenation level-dependent (BOLD) responses, revealing distinct spatial and temporal patterns. The data reported show that hypercapnia induces a strong hemodynamic response, while neuronal activity is inhibited. This suggests a dissociation between vascular and neural activity, an observation which is important to consider when discussing the relationship between neuronal firing and fMRI signals. By integrating optical imaging with fMRI, the study provides valuable insight into cerebrovascular regulation and its implications for interpreting fMRI data. The study also contributes to an ongoing debate on the role of CO2 as a neuronal signal to the vasculature published in recent years in Nature Communications (PMID: 35440557; PMID: 39223128; PMID: 39223137). The findings support earlier observations (first observed by John Scott Haldane more than 100 years ago) that CO2 inhibits brain neuronal activity and can override NVC relationship. I have the following comments for authors to consider in their revision:

Reply: We thank the reviewer for recognising both the technical advances of our dual-modal platform and the relevance of its application to the long-standing debate on CO_2-driven neurovascular uncoupling. In the revised manuscript we have further strengthened the study along the very lines the reviewer highlighted. Please find the point-by-point responses to the comments below.

1. While the study clearly demonstrates a neurovascular dissociation, it does not delve into the underlying cellular or molecular mechanisms. Are astrocytes, endothelial cells, or pericytes mediating this effect? Further investigations using pharmacological or genetic tools would be needed to determine the mechanistic basis of these findings.

Reply: We agree that identifying the cellular players in the hypercapnia-induced neurovascular dissociation is a critical next step. To obtain first evidence, we carried out cell-type–specific calcium imaging in a new cohort of mice. Using a dual-AAV approach, we expressed GCaMP6s under the astrocytic GFAP promoter and RCaMP1.07 under the neuronal hSyn1 promoter in the upper-limb sub-region of primary somatosensory cortex. Cell-type expression was verified by confocal microscopy. During alternating normocapnic and 5 % CO_2 blocks, the animals (n = 4) received fore-paw stimulation (0.5 mA, 20 s) while we simultaneously recorded BOLD, neuronal RCaMP, and astrocytic GCaMP signals with our dual-wavelength enabled hybrid set-up (new Supplementary Figure S2).

Neuronal and astrocytic responses were unchanged by hypercapnia: peak ΔF/F, onset latency, and time-to-peak did not differ between gas conditions. In contrast, the stimulus-evoked BOLD responses were suppressed during hypercapnia in every animal. These data show that neither impaired neuronal firing nor diminished astrocytic calcium rises can account for the loss of functional hyperemia. This shows that the decoupling must arise downstream, most plausibly at the level of vasoactive signalling from astrocytes/endothelium to vascular smooth-muscle cells or pericytes. The new experiments are documented in Fig. 5 and described in the Results (lines 188–200) and discussion (lines 215-221).

2. Calcium imaging, though precise, captures activity only in superficial cortical layers, whereas fMRI measures whole-brain hemodynamics, leading to potential spatial mismatches in the data. This needs to be discussed.

Reply: We concur that the distinct imaging depths of our two modalities raise the possibility of spatial mismatch. We have addressed this on two levels. We tried to ensure an optimal spatial matching by extracting a three-dimensional mask comprising only layers I–IV from the Allen CCF of the 25 bilateral cortical areas, then orthogonally projected that mask into 2-D to build the fluorescence template shown in Fig. 1c. Every metric that links calcium to BOLD was computed from time-courses averaged over these matched voxels. All deeper-layer and sub-cortical BOLD voxels were excluded. Even with this cortical masking, BOLD signals in layers I–IV inevitably contain contributions from deeper areas, and fluorescence from dendrites may propagate laterally by scattering. Thus, the two read-outs are not in perfect one-to-one correspondence. In the revised manuscript, we acknowledged this potential issue in the Discussion (lines 260-263).

3. The authors should consider the pH sensitivity of the GCaMP sensor as fluorescence is reversibly quenching by acidic pH and may not accurately reflect the neuronal activity during hypercapnia.

Reply: We thank the reviewer for drawing attention to the reversible pH-sensitivity of GCaMP and its potential to confound our interpretation of neural activity under hypercapnia. To ensure that our fluorescence signals truly reflect intracellular calcium transients and not artifactual quenching caused by acidification, we have undertaken a comprehensive set of experimental and analytical improvements, and implemented in the revised manuscript when characterizing the changes in neuronal activity. First, we directly quantify the magnitude of fluorescence quenching in GCaMP6f upon intracellular acidification induced by both 5 % and 10 % CO_2, using the pH shifts and pH-sensitivity curves of GCaMP6f (Supplementary Note 1). We then derive correction factors, S_{6f} = 1.06 for 10 % CO_2 and S_{6f} = 1.025 for 5 % CO_2, when applied to measured spike amplitudes, restore the true underlying calcium dynamics (updated Fig. 1e-f, Supplementary Fig. S3).

Second, to further isolate genuine calcium signals from hemodynamic and optical artifacts, we have upgraded our imaging setup to a hybrid dual-wavelength approach. By complementing 488 nm excitation (GCaMP) with 561 nm excitation (hemoglobin absorption contrast), we capture simultaneous calcium- and blood-volume–sensitive signals (Supplementary Fig. S2). We then regress out the hemoglobin-derived component which accounts for CO_2-driven cerebral blood flow changes and apply a bandpass filter to remove slow drifts and high-frequency noise. This preprocessing pipeline yields an Hb-corrected GCaMP6f trace in which residual fluctuations are dominated by true calcium transients (Fig. 1e-f, Supplementary Fig. S3).

Third, we have collected data from a new cohort of GCaMP6f animals (n = 5) under both 5 % and 10 % CO_2 challenges and applied our full correction strategy: hemoglobin regression, bandpass filtering, and pH-scale-factor normalization. In these data, we observe that unscaled fluorescence spike amplitudes decrease by ~13.9 % during 5 % CO_2 and ~27.6

% during 10 % CO_2 (Fig. 1k-l, Supplementary Fig. S4). After applying the respective scale factors (1.025 or 1.06), the apparent amplitude drops are reduced to ~12.1 % and ~24.6 %, respectively, confirming that a significant component of the signal change reflects true modulation of neuronal activity rather than purely pH-driven quenching.

Finally, to guard against under-correction, we also applied the larger 10 % CO_2 scale factor (S_6f = 1.06) to the 5 % CO_2 data. Even with this conservative adjustment, spike amplitudes remained reduced by ~9.6 %, demonstrating that the observed decrease in neural activity under mild hypercapnia cannot be attributed solely to pH-dependent quenching (Supplementary Fig. S4).

Moreover, explaining the 27 % drop in spike amplitude at 10 % CO_2 would require a scale factor of ~1.37, equivalent to an intracellular pH decrease from 7.30 to 6.62 (Supplementary Note 1). Similarly, the 13.9 % loss at 5 % CO_2 would demand a pH drop to 6.88. Yet even a 10 min inhalation of 20 % CO_2 lowers intracellular pH only to ~6.85 [1]. Because such extreme acidification never occurs under our conditions, we can confidently conclude that the residual reductions in spike amplitude reflect genuine suppression of neuronal calcium dynamics rather than sensor artifact.

Together, these quantitative calibrations, improved optical recording methods, and controls leave us confident that our fluorescence measurements accurately track neuronal calcium dynamics during hypercapnic challenges and the activity changes we report reflect genuine neurophysiological modulation, not sensor artefact. We report the data in updated Fig. 1e-f, Fig. 1k-l and Supplementary Figs. S2-S4. Text is correspondingly updated (lines 86-93 and 204-206).

[1] Pospelov AS, Puskarjov M, Kaila K, Voipio J. Endogenous brain-sparing responses in brain pH and PO2 in a rodent model of birth asphyxia. Acta Physiol (Oxf). 2020;229(3):e13467. doi:10.1111/apha.13467

Point by point responses to reviewers' comments

Reviewer #1:

The authors have done a great job addressing my concerns and expanding the manuscript. They study is more timely and impactful with this new data.

Reply: We thank the reviewer for the encouraging words. Please find our point-by-point responses below.

Major

1) I have one more major point regarding the new data: the authors need to ensure that the lack of NVC BOLD responses they measured in hypercapnia is not due to run down from successive forepaw stimulations with little time (90sec) between each, in which the system does not have adequate time to fully recover. As the authors are always starting with normocapnia, and then switching to hypercapnia, and giving two stimulations in each condition, run down could be an issue, and the authors do not show washout and recovery back in normocapnia. The exact order of all stimulations and conditions is not provide I believe. Note, the neural and astrocyte calcium response might not run down, but the vessel still could; thus, looking at the calcium data is not an adequate control per se. The authors have this data already (Fig S5) and they just need to look at the two responses given 90 sec apart and see if they are the same amplitude in normocapnia, or compare across different stimulations to determine if there is run down. If there is no run down, great, but if there is run down to the point that the current interpretation is invalid, they could repeat the experiment with a lot more time in between each forepaw stimulation (I recommend around 5 min), or show recovery of NVC by going back to normocapnia and repeating again.

Reply: We thank the reviewer for raising this point. We actually designed the paradigm precisely to guard against it. As stated in the Methods (lines 356–357), two stimulation cycles were delivered within each gas block and no stimulation occurred during the initial normocapnic baseline. Hence, critically, the first evoked epoch in each run was under hypercapnia, so any putative rundown from prior stimulation in normocapnia cannot account for the loss of the BOLD NVC response under hypercapnia.

To directly test for any potential rundown, we additionally re-analysed the 20 s stimulation condition (i.e., the paradigm with the shortest recovery between successive stimulations) by comparing the forepaw-evoked BOLD responses during the second vs. third normocapnia periods (i.e., the first and second normocapnia periods in which stimulation was applied). We observed no decline in BOLD peak amplitude between these epochs across animals (new Supplementary Fig. S7, lines 100-102). Taken together, the block order and these quantitative checks rule out stimulus rundown as the cause of the absent BOLD response under hypercapnia. We have also clarified the exact order of gas blocks and stimulations by visualizing the stimulation scheme in Supplementary Fig. S7.

Minor

2) I do not love the title of this manuscript. It is not very informative and it focuses more on the method than the finding. The authors could consider focusing on their outstanding method in the abstract and have title read something like this: Hypercapnia uncouples neuronal and hemodynamic responses impairing functional brain connectivity and neurovascular coupling. This is just a suggestion.

Reply: We thank the reviewer for the great suggestion. We updated the title accordingly to focus on the main finding: "Hypercapnia dissociates neuronal and hemodynamic responses impairing neurovascular coupling and functional brain connectivity."

3) Line 39, the authors say "These vasoactive molecules engage various signaling pathways in vascular smooth muscle cells to induce vasodilation and increase cerebral blood flow (CBF) in response to neuronal activity." Please add "and pericytes" after vascular smooth muscle cells.

Reply: Manuscript is revised accordingly (line 39).

4) Line 100 the authors say "Conversely, neuronal spike amplitudes decreased by 12.18 ± 2.04% at 5% CO_2 and 24.59 ± 3.70% at 10% CO_2 (Fig. 1l), confirming that hypercapnia directly attenuates neuronal excitability despite unaltered vascular responsiveness." I do not understand the statement "unaltered vascular responsiveness". I think the author's

are referring to the global increase in the BOLD signal to CO2 and not the impaired NVC which is discussed in this section. Perhaps the authors want to say: "despite unaltered global vascular responsiveness to inhaled CO2."

Reply: We thank the reviewer for bringing this up. Indeed, we are referring to the global increase in the BOLD signal under hypercapnia. We have revised the sentence accordingly (line 107).

5) Supplemental Fig 1 could also show the new 561 laser line. Or make the laser box more general.

Reply: Supplemental Fig. S1 is revised accordingly.

Reviewer #2:

1) Gezginer et al. present new experiments that add useful information to the manuscript; however, the interpretation of several major observations remains questionable, if not invalid. Previous BOLD fMRI studies (e.g., Stefanovic et al., 2006; Gauthier et al., 2011; and others) have demonstrated the importance of quantifying both resting and evoked CBF to correctly interpret BOLD responses to hypercapnia. These studies should be cited and discussed, as they suggest other explanations for the disappearance of BOLD responses upon CO2. Can Gezginer's work contribute to resolving the ongoing controversy over the effects of hypercapnia on neuronal activity and neurovascular coupling (NVC)? Yes, but only if the authors, which possess all the necessary tools to address this question, perform the key controls which are still missing. First, the effect of CO2 on neuronal activity: bulk GCaMP6 fluorescence is insufficient to draw definitive conclusions, as GCaMP6 is pH-sensitive and thus a poor stand-alone reporter of activity. Corrections for pH sensitivity and hemoglobin absorption are only approximate and should be recalculated (see below). Direct measurements of neuronal activity—via multielectrode recordings or cellular-resolution calcium imaging—are required. A consistent reduction in firing rate or single-cell calcium transient frequency during CO_2 exposure would provide unambiguous evidence of reduced neuronal activity. The second important issue concerns the effect of CO_2 on NVC: Supplementary Figure S2 shows that the authors can measure hemodynamic responses independently of BOLD fMRI. CBV responses to hypercapnia should be recorded, and CBV traces added to Figure 1i, to directly assess NVC. Without these controls, the conclusions remain speculative.

Reply: We agree that evoked hemodynamics should be assessed independently of BOLD under hypercapnia and thank the reviewer for the suggestion. Accordingly, we analyzed the concurrently acquired intrinsic hemoglobin channel (561-nm ISOI, HbT/CBV-weighted) and we now plot these CBV/HbT time courses alongside BOLD and FL (new Fig. 1k, updated Supplementary Fig. S6, line 98-100). The stimulus-evoked HbT/CBV responses were either markedly attenuated or absent at 5% CO_2, while neuronal Ca^{2+} transients were preserved. Thus, the loss of the BOLD NVC response is accompanied by a loss of the CBV/HbT response, directly demonstrating impaired neurovascular coupling under hypercapnia rather than a BOLD-specific dynamic-range artifact. We have added a discussion of prior calibrated-fMRI work that proposed baseline CBF/CBV–driven compression of BOLD (Stefanovic 2006, Gauthier 2011) and explain how the concurrent attenuation of evoked CBV/HbT in our data contradicts interpretations based solely on baseline shifts (see revised Discussion, lines 227–232).

We appreciate the reviewer's concerns about CO_2 effects on neuronal activity and the pH-sensitivity of bulk GcaMP. We address these in detail in our response to point (3).

Additional detailed comments:

2) In the manuscript, the definition of "spikes" is unclear. With the limited spatial and temporal resolution of the fluorescence acquisition system, the detected events cannot represent true neuronal action potentials. They are more likely spontaneous calcium fluctuations from neuron or astrocyte populations, depending on the expression patterns of GCaMP6 and RCaMP7.01. The term "spikes" should be avoided, as in neurons these signals could reflect synaptic potentials, action potentials, or second messenger–mediated calcium transients; in astrocytes, their meaning is even less certain. Moreover, the presentation of fluorescence data is inconsistent and prone to misinterpretation. In some cases, the authors appear to show the amplitude or envelope of Ca^{2+} "spikes," while in others they display changes in baseline GCaMP6 fluorescence. Without a clear and consistent definition of what is being measured, most neuroscientists will find the results difficult to interpret.

Reply: We agree that "spikes" is an imprecise term in the context of wide-field calcium imaging. We therefore replaced it with terminology better reflecting what has actually been measured throughout the manuscript and supplementary

material. Specifically, we now refer to "(population) Ca^{2+} transients" (neuronal channel). Additionally, we revised the manuscript to explicitly define what is displayed for the envelope of Ca^{2+} transients and event-related responses (line 391-395).

3) The authors introduced a theoretical correction for the pH-dependent reduction of GCaMP6 fluorescence under hypercapnia. The pKa used is that of GCaMP6 in a saturated Ca^{2+} condition (Chen et al.), which corresponds to a situation with ~400 times more Ca^{2+} (39µM) than resting physiological condition (~100 nM). The 'apo' pKa (Ca^{2+}-free) - while not fully physiological—is closer to resting neuronal conditions. Using the pKa_apo of 8.77 would yield a scale factor S6f = 0.032/0.022 = 1.45 (instead of 1.06), dramatically increasing the correction required and highlighting the significant impact of acidosis on GCaMP6 fluorescence. Given the high uncertainty of the correction factor, neuronal activity should be corroborated using complementary techniques.

Reply: We believe there is a misunderstanding where the pH factor has been applied and which biochemical state governs the signal we quantify. We apply pH scaling only to the amplitude of resting-state Ca^{2+} transient peaks after simultaneous hemoglobin regression and band-pass filtering, steps that remove hemoglobin absorption confounds and slow baseline drifts. The residual fast fluctuations (whose peaks we measure) are dominated by the Ca^{2+}-bound ensemble, whereas the apo fraction contributes primarily to baseline and slow components that are suppressed by our preprocessing. For peak scaling, the relevant pH sensitivity is therefore the Ca^{2+}-saturated pK_a, not the apo pK_a that characterizes the Ca^{2+}-free chromophore at baseline. This stems directly from the photophysics of GCaMP6 variants, in which Ca^{2+} binding to calmodulin-M13 shifts the chromophore to a brighter, more anionic state with a lower effective pK_a. Hence, during transient peaks, the photon budget is dominated by the Ca^{2+}-bound chromophore. Using the apo pK_a to rescale peak amplitudes would mis-specify the emitting ensemble.

Independent, pH-insensitive measurements consistently show suppression of neuronal activity under moderate hypercapnia in the same regimes we test. In anesthetized macaques, intracortical recordings show reduced multiunit activity and reduced local field potential power [1]; in rodents, hypercapnia reduces multiunit activity and desynchronizes ongoing activity [2,3]; in humans, EEG and MEG show reduced oscillatory power and frequency during mild hypercapnia [4–9]; mechanistically, CO_2-induced acidosis lowers cortical excitability via adenosine and ATP signalling [10]. These observations corroborate reduced neural drive without reliance on fluorescence sensors.

Note also that our primary conclusions do not hinge on the exact magnitude of resting state Ca^{2+} attenuation. The abolition of stimulus-evoked hemodynamic responses under hypercapnia despite preserved neuronal and astrocytic Ca^{2+} transients, as well as the loss of cross-modal alignment between fluorescence and BOLD in dynamic functional connectivity and co-activation analyses, are both preserved irrespective of pH-scaling choice. This is because the corresponding inferences are derived from correlation-based and state-based metrics that are insensitive to multiplicative rescaling. We acknowledge that absolute Ca^{2+} transient peak amplitudes carry bounded uncertainty, since hemoglobin regression and pH scaling approximate removal of vascular and acid–base influences, and we now note this limitation in the manuscript (line 270-274).

Regarding the request for additional corroboration, it could potentially be done with invasive methods such as two photon calcium imaging or intracranial electrophysiology. Such experiments cannot be done with our current experimental system and preparation, thus would require a new experimental platform that would necessarily sacrifice the simultaneous cortex-wide coverage offered by our current system. Importantly, such corroboration would not affect our central conclusions, which do not depend on the absolute calibration of resting-state Ca^{2+} amplitudes. Note that the literature already provides robust, pH-independent evidence of hypercapnia-related neural suppression [1–10], so there deems to be limited value in repeating this result in a different apparatus as per our stated aims. Accordingly, adding highly invasive stand-alone measurements would not alter any primary conclusions nor materially increase the amount of new information, while substantially increasing invasiveness and animal use. This would therefore amount to a separate study with a different scope, aim, system, and methodology.

[1] Zappe AC, Uludağ K, Oeltermann A, Uğurbil K, Logothetis NK. The influence of moderate hypercapnia on neural activity in the anesthetized nonhuman primate. Cereb Cortex. 2008;18(11):2666-2673. doi:10.1093/cercor/bhn023

[2] Jones M, Berwick J, Hewson-Stoate N, Gias C, Mayhew J. The effect of hypercapnia on the neural and hemodynamic responses to somatosensory stimulation. Neuroimage. 2005;27(3):609-623. doi:10.1016/j.neuroimage.2005.04.036

[3] Martin C, Jones M, Martindale J, Mayhew J. Haemodynamic and neural responses to hypercapnia in the awake rat. Eur J Neurosci. 2006;24(9):2601-2610. doi:10.1111/j.1460-9568.2006.05135.x

[4] Wang D, Yee BJ, Wong KK, et al. Comparing the effect of hypercapnia and hypoxia on the electroencephalogram during wakefulness. Clin Neurophysiol. 2015;126(1):103-109. doi:10.1016/j.clinph.2014.04.012

[5] Hall EL, Driver ID, Croal PL, et al. The effect of hypercapnia on resting and stimulus induced MEG signals. Neuroimage. 2011;58(4):1034-1043. doi:10.1016/j.neuroimage.2011.06.073

[6] Thesen T, Leontiev O, Song T, et al. Depression of cortical activity in humans by mild hypercapnia. Hum Brain Mapp. 2012;33(3):715-726. doi:10.1002/hbm.21242

[7] Li, S., Zhao, Y., Wang, Q. et al. Electroencephalographic depression after abruptly increasing partial pressure of end-tidal carbon dioxide: a case series. BMC Anesthesiol 24, 373 (2024). https://doi.org/10.1186/s12871-024-02764-7

[8] Babiloni C, Vecchio F, Altavilla R, et al. Hypercapnia affects the functional coupling of resting state electroencephalographic rhythms and cerebral haemodynamics in healthy elderly subjects and in patients with amnestic mild cognitive impairment. Clin Neurophysiol. 2014;125(4):685-693. doi:10.1016/j.clinph.2013.10.002

[9] Driver ID, Whittaker JR, Bright MG, Muthukumaraswamy SD, Murphy K. Arterial CO2 Fluctuations Modulate Neuronal Rhythmicity: Implications for MEG and fMRI Studies of Resting-State Networks. J Neurosci. 2016;36(33):8541-8550. doi:10.1523/JNEUROSCI.4263-15.2016

[10] Dulla CG, Dobelis P, Pearson T, Frenguelli BG, Staley KJ, Masino SA. Adenosine and ATP link PCO2 to cortical excitability via pH. Neuron. 2005;48(6):1011-1023. doi:10.1016/j.neuron.2005.11.009

4) The authors report robust BOLD signals in subthalamic regions under hypercapnia, but Figure 1a suggests no cortical response—an unlikely scenario. Given that FL signals are acquired in superficial cortical layers, the authors must show CO_2 and NVC data from the same cortical regions where FL and BOLD signals were acquired, to ensure consistency.

Reply: We appreciate the opportunity to clarify. In Fig. 1a, we apply a strict threshold (t = 6) to highlight the strongest activations, which prominently showcases deep and subcortical structures but inevitably underrepresents widespread cortical responses. Hypercapnia produced robust, positive BOLD changes across cortex in every animal. To make this explicit and to ensure spatial consistency with the fluorescence readout, we now provide (i) an unthresholded group activation map and (ii) the group-level BOLD time course extracted from the same cortical ROI used for FL, showing clear CO_2-evoked increases (new Supplementary Fig. S3, main text line 82).

5) Figure S22 (bottom left) shows conflicting results: the AAV-injected group (n=4) appears to show a 2% BOLD increase under hypercapnia following electrical stimulation—higher than their normocapnia response (0.4-1.9%). How to conciliate this with the other experiments?

Reply: We apologize for the confusion and thank the reviewer for catching this error. The purpose of Supplementary Fig. S24 (formerly S22) is to investigate the anaesthesia-dependence of key results and the visualized data on the AAV-injected cohort is a duplicate of Fig. 5e. However, the BOLD panel (bottom left) was inadvertently plotted with incorrect (FL) y-axis values due to a formatting error. We have corrected the axis in the updated Supplementary Fig. S24.

6) Among the newly presented data, the authors report a marked decrease in astrocytic Ca^{2+} signal (or “spike” amplitude?) during hypercapnia, closely matching in magnitude and dynamic the decrease observed in neurons. In fact, Figure S21 shows a remarkably high correlation coefficient (r = 0.98) between neuronal (RCaMP1.07) and astrocytic (GCaMP6) signals in response to CO_2. While the authors attribute the neuronal GCaMP6 (or “spike amplitude”) decrease to inhibition, no explanation is provided for astrocytes. If not a pH-related artifact, what accounts for such a decrease in astrocytic Ca^{2+}? Has such a phenomenon been previously reported?

Reply: In the dual-indicator cohort (Supplementary Fig. S23; formerly S21), both the neuronal (RCaMP1.07) and astrocytic (GCaMP6s) traces are shown as raw ΔF/F. In the dual-indicator experiments, the 561-nm path was allocated to RCaMP so no separate simultaneous hemoglobin reference was recorded for regression, and no pH normalization was applied in this cohort. During hypercapnia, pH-dependent quenching of both indicators and hemoglobin absorption

(stronger in the green band) produce slow downward drifts. These shared optical influences explain the highly correlated neuron–astrocyte co-drifts during CO_2 and are not interpretable as biological suppression of astrocytic Ca^{2+} activity.

Accordingly, we do not report or interpret any reduction in astrocytic Ca^{2+} from these baseline drifts. Our inferences for this cohort are drawn from event-locked (GLM) responses, where neuronal and astrocytic Ca^{2+} transients evoked by forepaw stimulation are preserved under 5% CO_2, while the stimulus-evoked BOLD response is abolished (Fig. 5). To avoid any misinterpretation, we revised the caption to Supplementary Fig. S23.

Reviewer #3:

In their revision, the authors substantially expanded the study with new experiments addressing the main critiques. They (1) performed sensory stimulation at multiple intensities and durations during both normocapnia and hypercapnia, showing that hypercapnia suppresses the BOLD response without reducing neuronal calcium transients, thus demonstrating that functional hyperemia is occluded downstream of neuronal activation; (2) added a 10 % CO_2 condition to test for vascular "ceiling effects," finding further BOLD signals beyond the 5 % level and ruling out maximal dilation as the cause of neurovascular uncoupling; (3) repeated key tests under a different anesthetic regimen (isoflurane/medetomidine) and found consistent results; (4) implemented dual-wavelength optical recordings with hemoglobin signal regression and pH calibration to correct for optical artefacts and sensor quenching, confirming that hypercapnia genuinely reduces neuronal activity (well described in the earlier literature); and (5) incorporated cell-type–specific imaging (neurons and astrocytes). These additions address prior methodological concerns and strengthen the conclusions. Overall, their arguments appear well-supported: they convincingly show that the observed decoupling is not an artefact of hemodynamic contamination, pH effects, anesthesia choice, or vascular saturation.

Reply: We thank the reviewer for the encouraging comments and the thoughtful assessment!

Point by point responses to reviewers' comments

Reviewer #1:

The authors have satisfactorily addressed all my comments.

Reply: We thank the reviewer for the constructive and positive feedback!

Reviewer #2:

1) Gezginer et al. present some new data to answer our comments. Haemoglobin is known to have an oxygenation dependent absorption spectrum in the visible and near-infrared ranges, a property that underlies intrinsic optical imaging. Typically, to monitor total blood flow with IOSI, one classically uses a high-contrast isosbestic point of the haemoglobin absorption spectra - a wavelength at which both HbO and HbR have the same absorption values. This makes the measurement independent of oxygenation and therefore sensitive only to changes in [HbT] (see Ma et al., Phil.Trans.R.Soc.B, 2016). We therefore do not understand why the authors used the single wavelength of 561 nm, which is precisely a wavelength at which both HbO and HbR vary, in opposite directions, thereby reducing any detectable changes in HbT and thus providing a poor measure of CBV. The authors now show, in Fig. 1k, small CBV responses to hind paw stimulation that decrease during hypercapnia. The point is that the CBV signal is not optimized (due to the wrong wavelength used), and since CO_2 modifies O_2 binding to haemoglobin (Bohr effect) and thus alters the HbO/HbR ratio, no reliable conclusion can be drawn from measurements at only 561 nm. Consequently, the conclusion that "the loss of the BOLD NVC response is accompanied by a loss of the CBV/HbT response" is incorrect. The issue of whether CBV responses accompany or not the "absence" of BOLD responses remains unresolved and crucial for the validity of the paper. The authors must use an appropriate laser wavelength to measure CBV changes during NVC as well as in response to the CO_2 challenge, which is still missing. This later straightforward experiment is essential because the authors regressed calcium transients using the "hemoglobin" signal (Fig. 1e,f).

Reply: We apologize if some of our statements made the reviewer believe that our study was aimed at providing quantitative, oxygenation-independent HbT or CBV measurements. We fully agree that 561-nm is not strictly an isosbestic wavelength with the nearest high-contrast hemoglobin isosbestic region being in the ~570-nm band. Hence, the ISOI readings can be influenced by the relative balance of ΔHbO and ΔHbR. Consequently, hypercapnia-related changes in baseline oxygenation physiology, including shifts in blood gas chemistry and oxygen binding, can alter the composition of a single-wavelength intrinsic signal. As a result, single-wavelength measurement at 561 nm should not be treated as a quantitative, oxygenation-independent HbT or CBV metric. We have revised the manuscript to (1) explicitly refer to this channel as a "hemoglobin-weighted intrinsic reflectance signal" (ΔR/R at 561 nm) rather than "HbT/CBV", and (2) add a concise description of the spectral weighting and its implications under hypercapnia (lines 100–103, 318–322).

At the same time, it is also important to clarify that the 561 nm measurements had a different purpose, which is not affected by the fact that this wavelength cannot be used for retrieving calibrated or oxygenation-independent HbT/CBV traces. Rather, it provides a simultaneously measured intrinsic reflectance signal that tracks hemoglobin-driven optical fluctuations that contaminate wide-field fluorescence through time-varying absorption (and, to a lesser extent, scattering). In wide-field GCaMP imaging, this crosstalk is governed by hemoglobin-dependent optical density changes along the excitation and emission paths and therefore reflects both blood-volume-related and oxygenation-related contributions through ΔHbO and ΔHbR. Accordingly, for regression-based correction, the reference channel need not be HbT-specific; instead, it must capture the dominant absorption-driven variance that couples into the fluorescence channel. In this context, regression is a statistical nuisance-removal step: we remove the component of the GCaMP time series that covaries with the hemoglobin-weighted reflectance signal, without interpreting the reflectance trace itself as HbT/CBV. Because oxygenation-related absorption changes contribute to the very confound we aim to remove from fluorescence, a non-isosbestic hemoglobin-sensitive wavelength remains suitable for regression, whereas strict isosbesticity is primarily required when the goal is to interpret the reflectance signal itself as an oxygenation-independent HbT/CBV metric.

We also acknowledge that hypercapnia-induced oxygenation shifts could alter the relative weighting of HbO versus HbR contributions at 561 nm and thus modulate the apparent ΔR/R amplitude. However, this does not undermine the central conclusion of evoked neurovascular uncoupling for two reasons. (i) We quantify ΔR/R relative to the immediate pre-stimulus baseline within each block, which reduces sensitivity to slow baseline offsets between gas conditions and focuses the analysis on stimulus-locked reactivity. (ii) While 561 nm is not HbT-specific and partial ΔHbO/ΔHbR cancellation is possible at a single wavelength, 561 nm remains strongly hemoglobin-weighted; therefore, a robust stimulus-evoked vascular response would be expected to produce a measurable intrinsic reflectance modulation. The near-complete suppression of stimulus-locked intrinsic reflectance transients during hypercapnia, together with preserved neuronal/astrocytic Ca^{2+} responses, is therefore unlikely to be explained solely by spectral weighting and instead supports attenuated stimulus-evoked hemodynamic reactivity under hypercapnic conditions. Importantly, we now describe this optical readout conservatively as hemoglobin-weighted (not quantitative CBV/HbT).

2) Regarding the calcium spikes, which are now referred to as calcium transients, we remain in the dark about their nature. Could the authors at least show, in a supplementary figure, what they are referring to? We suggest simply expanding portions of the traces shown in Fig. 1e and f. This would help the reader understand the shape of these fluctuations. In their current presentation, they appear to represent some form of background noise (which amplitude could be modulated by pH). Are these fluctuations symmetrical? Is it possible to analyze their frequency? These transients seem to contaminate the data. For example, in the upper black trace in Fig. 1j, if one draws a line through the resting period (the first 5 seconds before stimulation), it appears that there is no fluorescence increase and possibly even a decrease after 10 seconds. Could the authors comment on this point? Clearly, more information is needed about these calcium "transients."

Reply: We thank the reviewer for raising this important point. We agree that, in the original submission, the fast fluctuations in the wide-field GCaMP signal were insufficiently visualized and could therefore be misconstrued as background noise. In the revised manuscript, we have expanded both the presentation and analysis to explicitly characterize these events in the time domain and frequency domain, and we now include zoomed-in trace segments as suggested.

Wide-field GCaMP imaging does not report single-unit spiking; rather, it measures mesoscale population activity with strong contributions from neuropil/dendritic signals (particularly in Thy1-GCaMP lines). Importantly, cortex-wide GCaMP signals robustly contain a fast component on second time scales (delta-range), which has been repeatedly shown to reflect physiologically meaningful neural dynamics (not camera noise) and to yield reproducible functional connectivity (FC) structure across cortical areas. For example, Wright et al. demonstrated that band-limited wide-field calcium activity in the delta range produces stable FC maps with clear network organization and strong state dependence (awake vs anesthetized) [1]. Likewise, simultaneous wide-field Ca^{2+}–fMRI work has shown that separating calcium into a slow component aligned to BOLD and a faster component reveals meaningful and interpretable network structure that is partially shared with, but also distinct from, BOLD-derived organization [2]. These prior results provide a clear framework for interpreting the fast transients in our recordings as an expected component of mesoscale calcium dynamics rather than background noise.

Following the reviewer's suggestion, we now include expanded trace segments focusing on the fast calcium transients (new Supplementary Fig. S6). The expanded view demonstrates that these fluctuations are structured events with stereotyped kinetics (typically a rapid rise followed by a slower decay), rather than isolated outliers or camera noise. Importantly, these events are present under both normocapnia and hypercapnia; in our data, hypercapnia primarily modulates their amplitude envelope, rather than generating an entirely new fluctuation class. This event-like morphology and recurrence on second time scales closely match previously described mesoscale calcium transients in the wide-field literature (e.g., Figs. 6–7 in [1]; Fig. 2 in [3]; Fig. 5 in [4]). Because the traces in Fig. 1f/S6 reflect hemoglobin-regressed and band-pass–filtered signals (0.2–4 Hz), they are effectively zero-mean; hence the signal naturally oscillates around baseline and takes both positive and negative values. This does not indicate artifactual sign flipping; it reflects fluctuations around baseline after removal of slow drifts and vascular confounds. At the event level, perfect symmetry is not expected: GCaMP indicator kinetics and population dynamics typically yield faster rising phases and slower decays, producing the asymmetric waveform shape that is clearly visible in the expanded traces.

We now provide a frequency-domain analysis of the fast component (new Supplementary Fig. S7). The power spectral density (PSD) shows broadband power within the passband together with reproducible spectral structure across animals, rather than the flat spectrum expected for white camera noise, consistent with prior mesoscale calcium imaging reports [1].

Based on both prior literature and our data, these fast transients are not a contaminant to be removed; rather, they are a core feature of wide-field calcium dynamics and are widely leveraged to compute biologically meaningful FC. In our study, we compute an FC matrix from the fast calcium component (Fig. 3a) and use it as an empirical neural reference to assess which low-frequency FL/BOLD states best align with neural dynamics (Fig. 3b–c). Critically, we show that the fast-transient FC is highly stable across time windows in our recordings (Supplementary Fig. S15), which mirrors prior findings that calcium-derived FC maps are robust, interpretable, and state dependent [1–3].

Regarding the apparent attenuation and occasional undershoot of the corrected fluorescence trace during the 0.5 mA, 20 s stimulus, we emphasize that this behavior is well within the expected regime for prolonged sensory/electrical drive and does not imply that the response is noise. Sustained stimulation commonly evokes a strong onset response followed by adaptation during the ongoing train, attributable to well-established circuit mechanisms including thalamocortical short-term synaptic depression and activity-dependent recruitment of inhibition/excitation rebalancing [7]. Such adaptation produces a decay in calcium responses during a maintained stimulus and is expected to be more pronounced for long trains (20 s) than for short trains (8 s), consistent with our observation that the late decrease is not prominent in the 1 mA, 8 s and 0.5 mA, 8 s protocols. Finally, post-stimulation suppression (i.e., reduced activity after the train) has been directly observed in calcium imaging studies of prolonged electrical stimulation (including intracortical microstimulation), with suppression detectable in calcium and electrophysiological measures [8–10]; thus these studies demonstrate that prolonged electrical drive can elicit duration-dependent suppression and offset aftereffects consistent with the directionality of the late-phase decrease observed in our longest stimulation condition.

[1] Wright PW, Brier LM, Bauer AQ, et al. Functional connectivity structure of cortical calcium dynamics in anesthetized and awake mice. *PLoS One*. 2017;12(10):e0185759. Published 2017 Oct 19. doi:10.1371/journal.pone.0185759

[2] Vafaii H, Mandino F, Desrosiers-Grégoire G, et al. Multimodal measures of spontaneous brain activity reveal both common and divergent patterns of cortical functional organization. *Nat Commun*. 2024;15(1):229. Published 2024 Jan 3. doi:10.1038/s41467-023-44363-z

[3] Grienberger C, Adelsberger H, Stroh A, et al. Sound-evoked network calcium transients in mouse auditory cortex in vivo. *J Physiol*. 2012;590(4):899-918. doi:10.1113/jphysiol.2011.222513

[4] Vanni MP, Murphy TH. Mesoscale transcranial spontaneous activity mapping in GCaMP3 transgenic mice reveals extensive reciprocal connections between areas of somatomotor cortex. *J Neurosci*. 2014;34(48):15931-15946. doi:10.1523/JNEUROSCI.1818-14.2014

[5] Musall S, Kaufman MT, Juavinett AL, Gluf S, Churchland AK. Single-trial neural dynamics are dominated by richly varied movements. *Nat Neurosci*. 2019;22(10):1677-1686. doi:10.1038/s41593-019-0502-4

[6] Chen Z, Zhou Q, Deán-Ben XL, Gezginer I, Ni R, Reiss M, Shoham S, Razansky D. Multimodal Noninvasive Functional Neurophotonic Imaging of Murine Brain-Wide Sensory Responses. Adv Sci (Weinh). 2022 Aug;9(24):e2105588. doi: 10.1002/advs.202105588. Epub 2022 Jul 7. PMID: 35798308; PMCID: PMC9404388.

[7] Chung S, Li X, Nelson SB. Short-term depression at thalamocortical synapses contributes to rapid adaptation of cortical sensory responses in vivo. Neuron. 2002 Apr 25;34(3):437-46. doi: 10.1016/s0896-6273(02)00659-1. PMID: 11988174.

[8] Wu GK, Ardeshirpour Y, Mastracchio C, Kent J, Caiola M, Ye M. Amplitude- and frequency-dependent activation of layer II/III neurons by intracortical microstimulation. iScience. 2023 Oct 6;26(11):108140. doi: 10.1016/j.isci.2023.108140. PMID: 37915592; PMCID: PMC10616374.

[9] Hughes, C. L., Stieger, K. C., Chen, K., Vazquez, A. L., & Kozai, T. D. (2025). Spatiotemporal properties of cortical excitatory and inhibitory neuron activation by sustained and bursting electrical microstimulation. iScience, 28(6).

[10] Stieger KC, Eles JR, Ludwig KA, Kozai TDY. Intracortical microstimulation pulse waveform and frequency recruits distinct spatiotemporal patterns of cortical neuron and neuropil activation. J Neural Eng. 2022 Mar 31;19(2):10.1088/1741-2552/ac5bf5. doi: 10.1088/1741-2552/ac5bf5. PMID: 35263736; PMCID: PMC9171725.

Minor points:

3) Please describe the IOSI methodology in the methods section.

Reply: We have revised the Methods to clarify the IOSI methodology (line 308-317).

4) Please clearly indicate in Figure 1 and/or its legend the regions of interest (ROIs) from which the traces in panels i–m were extracted, in relation to panels a–b and g–h. We assume that panels i, j, and k correspond to the same ROI in the somatosensory cortex (as suggested by the heat maps in g–h), but this should be explicitly confirmed.

Reply: Indeed, the panels i-k correspond to the same ROI from somatosensory cortex. We now denote the ROI in Figs. 1g, h and explicitly mention it in the Fig. 1 legend.

5) The ketamine/xylazine anesthesia (100 mg/kg and 10 mg/kg, respectively) cannot be described as "light anesthesia" (lines 67 and 73), as these doses correspond to a surgical level of anesthesia, i.e. a deep anesthesia.

Reply: This is a valid point. Indeed, the induction dose of ketamine/xylazine (100/10 mg/kg) produces a deep surgical level of anesthesia. Importantly, all imaging was performed under a substantially lower maintenance dose (25/1.25 mg/kg), which corresponds to a lighter and more stable anesthesia plane commonly used in functional imaging studies. We have revised the text to avoid referring to the induction stage as "light anesthesia" and now explicitly state that fMRI acquisition was carried out under the reduced maintenance regimen, where the anesthetic depth is lighter (lines 346–348).

6) References 11, 13, and 14 are not correctly cited in the introduction.

Reply: We thank the reviewer for pointing out this issue. After re-examining the cited literature, we confirmed that the placement of Ref. 11 (Hosford et al., 2022) in the first sentence was appropriate, as this study reports the abolition of stimulus-evoked BOLD responses under hypercapnia. However, we agree that its inclusion in the second sentence was not accurate, as the mechanistic dissociation between hypercapnic and functional hyperemia is specifically addressed by Refs. 13 (Tournissac et al., 2024) and 14 (Gordon, 2024).

For clarity, the original sentences read: "Exogeneous CO_2 has been shown to occlude functional hyperemia, the increase in blood flow associated with sensory stimulation [11, 12]. Conversely, it has recently been reported that hypercapnic hyperemia and functional hyperemia do not activate the same vasodilatory signaling pathway mediating cerebrovascular responses [11, 13, 14]." To correct this mismatch, we have removed Ref. 11 from the second sentence so that only the studies directly demonstrating mechanistic divergence (Refs. 13 and 14) are cited.

7) Error bars are not consistently defined across figures; the only mention of SEM appears in line 418.

Reply: We have now ensured that error bars are explicitly defined in all relevant figures and figure panels, and we confirm that they consistently represent the SEM.

8) Several axes are difficult to read due to missing or misaligned tick marks. Please check carefully (e.g. Fig.1m).

Reply: We carefully reviewed all figures and corrected missing or misaligned tick marks. We also adjusted the tick mark placement so that any previously occluded ticks now appear clearly (e.g., by placing them outside the axis when necessary).

9) Line 354: the phrase "8/20 s onset time, and 90 s stimulus repetition cycle" is unclear. Based on Figure 1, this likely refers to an 8 or 20 s stimulus duration rather than onset time.

Reply: The phrase "8/20 s onset time" was indeed unclear. We have now revised the text to explicitly state that these values refer to stimulus train durations (line 365-367).

10) The Methods section states that respiration, heart rate, and SpO_2 were monitored, but no data are shown. These physiological parameters are important to interpret both spontaneous neural activity and baseline vascular tone and examples should be reported in a supplementary figure.

Reply: The physiological parameters are reported in the new Supplementary Fig. S27.

11) In Figure 5, legends e and f appear inverted.

Reply: We thank the reviewer for catching this error – the legends are now corrected in the revised manuscript.

Point by point responses to reviewers' comments

Reviewer #2:

Gezinger et al. have appropriately tempered the conclusions of some of their experiments. We understand that they do not wish to repeat their IOSI experiments using an appropriate laser wavelength, i.e., one at an isosbestic point of the hemoglobin absorption spectrum. Still, we have repeatedly asked the authors to present the IOSI data acquired at 561 nm during CO_2 stimulation. The authors possess the relevant data, and since the "pseudo CBV" signal is important to estimate the effect of hemoglobin on fluorescence signals, they should present the IOSI responses to CO_2 in a supplementary figure. If these responses mirror the dynamics of the BOLD responses shown in Supplementary Figure S10, then the use of the "CBV" signal could be considered acceptable. Otherwise and also in light of the effects of CO_2 on the BOLD signal (Kennerley et al., 2012), we continue to find the data difficult to interpret.

Reply: We apologize for the initial misunderstanding; we had interpreted the previous request for "CBV responses" as specifically referring to the electrical stimulation experiments. To address the reviewer's concern regarding CO_2 stimulation, we now include the IOSI data acquired at 561 nm during hypercapnia in a new Supplementary Fig. S12.

This figure illustrates the time-resolved IOSI response during 5% and 10% CO_2 inhalation alongside summary quantification. Critically, the IOSI responses closely mirror the BOLD dynamics previously shown in Supplementary Fig. S10. Furthermore, the across-animal scaling of the IOSI signal from 5% to 10% CO_2 correlates strongly with the corresponding BOLD amplitude increases (new Supplementary Fig. S12, line 108-110). These results validate our use of the 561 nm IOSI-derived signal as a reliable proxy for capturing the hemodynamic changes necessary to interpret and account for hemoglobin contributions to our fluorescence readouts.
